# Supplementary material for: Protocol for a randomised, multicentre, four-arm, double-blinded, placebo-controlled trial to assess the benefits and safety of iron supplementation with malaria chemoprevention to children in Malawi: IRMA trial
Source: BMJ Open. 2023 Oct 13;13(10):e069011. doi: 10.1136/bmjopen-2022-069011 (PMC10583080; doi:10.1136/bmjopen-2022-069011)
Supplement: Supplementary data [file bmjopen-2022-069011supp001.pdf]

Supplementary material 1: Multiple micronutrient powders (MNPs) contents

	**Micronutrient name**	**Quantity**
1.	Niacinamide	7.02 - 8.58 mg/g
2.	Riboflavin	0.653 - 0.798 mg/g
3.	Pyridoxine	0.585 – 0.715 mg/g
4.	Thiamine	0.675 – 0.825 mg/g
5.	Vitamin C	32.4 – 39.6 mg/g
6.	Vitamin A	522 – 667 µgRE/g
7.	Vitamin E (as Tocopherol Eq.)	5.04 – 6.16 mgTE/g
8.	Vitamin D3	6.75 – 8.63 µgD/g
9.	Vitamin B12	1.09 – 1.40 µg/g
10.	Folic Acid	109 – 134 µg/g
11.	Selenium	17.0 – 24.5 µg/g
12.	Iodine	9.00 -129.6 µg/g
13.	Iron	10.00 -12.10 mg/g
14.	Zinc	4.10 – 0.739 mg/g
15.	Copper	0.605 – 0.739 mg/g
