# Supplementary material for: Protocol for a randomised, multicentre, four-arm, double-blinded, placebo-controlled trial to assess the benefits and safety of iron supplementation with malaria chemoprevention to children in Malawi: IRMA trial
Source: BMJ Open. 2023 Oct 13;13(10):e069011. doi: 10.1136/bmjopen-2022-069011 (PMC10583080; doi:10.1136/bmjopen-2022-069011)
Supplement: Supplementary data [file bmjopen-2022-069011supp002.pdf]

Supplementary material 2: Main study activities per visit

		Intervention period (Active follow-up)													Post intervention period (Passive follow up)					
	**Month**	**M1**			**M2**		**M3**		**M4**		**M5**		**M6**		**M7**	**M8**	**M9**	**M10**	**M11**	**M12[‡]**
**Planned activity**	**Day** 0	D 1[#]	D 14	D 28	D 42	D 56	D 70	D 84	D 98	D 112	D 126	D 140	D 154	D 168[§]						
**Location**	**H/HF**	**HF**	**H**	**H**	**H**	**H**	**H**	**H**	**H**	**H**	**H**	**H**	**H**	**HF**	**H**	**H**	**H**	**H**	**H**	**HF**
Pre-screening/ screening, & informed consent	**X**	**X**																		
Randomization		**X**																		
Demographic, clinical, CREDI and SES assessments		**X**																		
Family Care Index (FCI)		**X**												**X**						**X**
Bayley-III Cognitive Composite Score (CogCS)														**X**						**X**
Bayley-III Motor (MotCS) and Language (LangCS) Composite Scores														**X**						**X**
Behaviour: Wolke's Behaviour Ratings (WBR)														**X**						**X**
Food security & dietary diversity questionnaires		**X**												**X**						**X**
Anthropometry[1] - (mother and child)		**X**												**X**						**X**
**Laboratory procedures**																				
Haemoglobin		**X**												**X**						**X**
Malaria microscopy		**X**												**X**						**X**

Serum iron marker tests[2]		**X**												**X**						**X**
Serum for inflammatory marker tests (CRP/AGP)[3]		**X**												**X**						**X**
**Active follow-up:**																				
Compliance assessment		**X**	**X**	**X**	**X**	**X**	**X**	**X**	**X**	**X**	**X**	**X**	**X**	**X**						
MNPs/Placebo MNPs[4](distribute, instruct)		**X**		**X**		**X**		**X**		**X**		**X**								
DP/Placebo DP[5] (distribute, instruct & observe use/intake)		**X**		**X**		**X**		**X**		**X**		**X**								
Iron syrup/Placebo iron syrup[6]		**X**		**X**		**X**		**X**		**X**		**X**								
Provide educational information		**X**	**X**	**X**	**X**	**X**	**X**	**X**	**X**	**X**	**X**	**X**	**X**	**X**						
Assess infections[7]			**X**	**X**	**X**	**X**	**X**	**X**	**X**	**X**	**X**	**X**	**X**	**X**	**X**	**X**	**X**	**X**	**X**	**X**
Adverse Events monitoring			**X**	**X**	**X**	**X**	**X**	**X**	**X**	**X**	**X**	**X**	**X**	**X**	**X**	**X**	**X**	**X**	**X**	**X**
Health care utilization monitoring		**X**	**X**	**X**	**X**	**X**	**X**	**X**	**X**	**X**	**X**	**X**	**X**	**X**	**X**	**X**	**X**	**X**	**X**	**X**

# - Baseline; § - Midline visit at day 168. Primary outcome was assessed at day 168 ± 14 days as per protocol)**; ‡ - Endline; M –** Month; **D –** Day; **H – Home** (visit at the Participant's Home)**; HF –** Visit at the Health Facility; **X** – scheduled activity. **CREDI -** Caregiver Reported Early Development Instrument**; SES -** Social Economic Status; [1]**Anthropometric assessments** (mother and child): height/length, weight; [2]**Serum iron marker tests**: Serum ferritin; [3]**Inflammation markers**: CRP (C-Reactive Protein) or AGP (alpha 1 acid glycoprotein); [4]**MNPs** containing 10.0mg iron and 14 other micronutrients, daily for 6 months; **Placebo MNPs**: (maltodextrin), daily for 6 months; [5]**DP/Placebo DP** (20mg dihydroartemisinin/ 160mg piperaquine in suspension) dosed by body weight, three consecutive days every 4 weeks for 6 months; [6]**Iron syrup/Placebo iron syrup:** 10mg daily for six months; [7]**Infections**(assessment): (rate and number of days affected) fever/respiratory/diarrhoea.
NB: Families were encouraged to permit collection of venous blood for venous haemoglobin and iron and inflammation biomarkers at all timepoints; however, if a family refused venous blood collection or if collection was unsuccessful, data for the primary outcome and other non-laboratory secondary outcomes was still collected.
