# Supplementary material for: Protocol for a randomised, multicentre, four-arm, double-blinded, placebo-controlled trial to assess the benefits and safety of iron supplementation with malaria chemoprevention to children in Malawi: IRMA trial
Source: BMJ Open. 2023 Oct 13;13(10):e069011. doi: 10.1136/bmjopen-2022-069011 (PMC10583080; doi:10.1136/bmjopen-2022-069011)
Supplement: Supplementary data [file bmjopen-2022-069011supp003.pdf]

IRMA Study

Training and Research Unit of Excellence (TRUE), Department of Public Health, College of Medicine, Blantyre, Malawi

University of Melbourne, Victoria, Australia

1. INFORMED CONSENT – SCREENING

Protocol No. 2213	Version No 4.0	Date: July 25, 2022

Protocol Title: Benefits and safety of IRon supplementation with MAlaria chemoprevention to children in Malawi (IRMA) - A randomised controlled trial

Investigator's name: Prof Kamija Phiri and Dr Sant-Rayn Pasricha

Organization: Training and Research Unit of Excellence (TRUE), Department of Public Health, College of Medicine, Blantyre, Malawi in collaboration with The Walter and Eliza Hall Institute, Melbourne, Australia

Purpose of the research: We intend to assess if a program of iron supplementation or Multiple Micronutrient Powder (MNP), with antimalarials, have an impact on child development, both immediately following intervention and after 6 months further follow up.

Background:
As you may know, anaemia and malaria are a major public health problem among pre-school children in Malawi. However, we do not yet know whether preventing or treating anaemia by giving iron supplements or Micronutrients to all children benefits child health. In this study we want to see the effect of malaria prevention and iron interventions on children's cognitive capacity both in short and long run. Our main objective of this consent form is to check if your child is eligible to participate in the study.

Why are you invited to participate in the study?
We are inviting your child to participate in this study, because your child is within the age of 6 months +/-14 days and you are living in the study area in Chikwawa.

Methods and procedures
If you agree, we will measure your child's haemoglobin level by taking one or two drops of blood from her/his finger tip. If your child Hb level is ≥7.0g/dL, and there are no other reasons for ineligibility, we will administer our screening questionnaire. We will then invite your child to participate in our study. You will be requested to come to a local test centre in a scheduled date to enrol in the study and sign a separate consent form for study's procedure. If your child's Hb level found to be <7.0g/dl, we will refer him/her to the local health centre for appropriate management.

Risk and benefits
There is no risk involved participating in this screening procedure. Blood sample will be collected from fingertip following all aseptic precautions by an expert technician. Only minimum momentary pricking pain might be experienced by your child. However,

Mwangi MN, *et al. BMJ Open* 2023; 13:e069011. doi: 10.1136/bmjopen-2022-069011

our expert technician will do the job with much care and precautions. We will inform you of your child's haemoglobin level and provide necessary advice if your child is found severely anaemic.

Privacy, anonymity and confidentiality
All information collected will remain strictly confidential

Future use of information
The information collected from you will help us decide whether your child is eligible to participate in the study. It will not be used in future.

Right not to participate and withdraw
Your participation is completely voluntary. You have the right to refuse to participate in this screening process or to refuse to participate in the main trial or to withdraw from the study at any time. Your decision will not affect getting any services from the health facilities.

Principle of compensation
There is no monetary compensation for your participation.

Answering your questions/ Contact persons
We would be obliged to provide you information related to the condition(s) of your child and would be happy to answer your questions about the study. You would be able to freely communicate with any investigator of this study (contact address provided below).

You can ask any questions you have about the study or about your rights. If you have other questions later, you can contact our study teams on the following numbers:

Chikwawa Boma: 0995 835 215 or 0883 536 917

Mfera: 0990 676 012 or 0881 861 069

Mapelere: 0990 676 014 or 0881 861 169

Makwhira: 0994 952 510 or 0883 536 925

If you want to know more about your rights as a research participant you can contact the NHSRC Director, at the Ministry of Health on the following details, Phone: 0999 397 913. Email: cmitambo@gmail.com or mohdoccentre@gmail.com

If you agree to our proposal of enrolling you and your child in our study, please indicate that by putting your signature or your left thumb impression at the specified space below.

Thank you for your cooperation

IRMA Study

Name of Parent/ Guardian

____ ______________________
Signature or right thumb impression of Parent/ Guardian/ Attendant — Date

____
Name of witness (*If Parent/ Guardian cannot read and write*)

____ ______________________
Signature of witness — Date

____
Name of Investigator or study staff

____ ______________________
Signature of Investigator or study staff — Date

Mwangi MN, *et al. BMJ Open* 2023; 13:e069011. doi: 10.1136/bmjopen-2022-069011

IRMA Study

Training and Research Unit of Excellence (TRUE), Department of Public Health, College of Medicine, Blantyre, Malawi

University of Melbourne, Victoria, Australia

1. Kalata Yopempha Chilolezo

Protocol No. 2213	Version No. 4.0	Date: July 25, 2022

Mutu wa kafukufuku: kufufuza ubwino ndi chitetezo cha mankhwala okhala ndi ironi komanso mankhwala oteteza ku malungo kwa ana muno m'Malawi

Oyang'anira kafukufuku: Prof Kamija Phiri ndi Dr Sant-Rayn Pasricha

Bungwe: Training and Research Unit of Excellence (TRUE), Department of Public Health, College of Medicine, Blantyre, Malawi in collaboration with University of Melbourne, Victoria, Australia

Cholinga cha kafukufuku
Tikuchita kafukufuku ndi cholinga chokuti tifufuze ngati mankhwala okhala ndi ayironi angathandize pa kakulidwe ka mwana potsatira kupereka mankhwalawa kwa miyezi isanu ndi umodzi motsatizana ndi kulondoloza kwa miyezi ina isanu ndi umodzi yowonjezera.

Zokhudzana ndi kafukufukuyi
Monga mukudziwa kuchepa kapena kusowa kwa magazi ndi vuto lalikulu makamaka kwa ana achichepere amene sanayambe sukulu kuno ku Malawi. Ngakhale zili chonchi, sitikudziwa ngati kuteteza mwana ku vuto limenelo losowa magazi popereka mankhwala okhala ndi ayironi kungathandize umoyo wa mwanayu. Mu kafukufukuyi tikufuna tiwone zotsatira zopereka mankhwalawa kwa ana makamaka pa kakulidwe kawo mu kaganizidwe ndi makhalidwe. Pakadali pano cholinga chathu pakucheza kwathu ndi inu chenicheni ndi chakuti tione ngati mwana wanu angakhale oyenera kutenga nawo mbali mu kafukufukuyu.

Kodi nchifukwa chiyani tasankha mwana wanu kuti atenge nawo mbali mukafukufuku?
Takuitanani kuti mutenge nawo mbali mukafukufukuyi makamaka polingana ndi kuti mwana wanu ali ndi miyezi isanu ndi theka kapena sanaposere miyezi isanu ndi umodzi kuonjezerapo theka, komanso mukukhala dera lino la Chikwawa lomwe tikufuna tichitiremo kafukufuku ameneyu.

Zochitika zokhudzana ndi kafukufukuyi.
Ngati mwagwirizana nazo kuti mwana wanu atenge nawo mbali, tiyeza kuchuluka kwa magazi a mwana wanu potenga dontho la magazi kuchokera pa chala chake. Ngati taona kuti magazi ake ndiokwanira ndipo kuti palibenso choletsa chilichonse, tikupemphani kuti tikufunseni mafunso angapo. Kenako tidzakupemphani kuti mwana wanu atenge nawo mbali mukafukufukuyi. Mudzafunsidwa kuti mudzabwere ku

chipatala pa tsiku lomwe tidzakuwuzani kuti mwana wanu adzalowe nawo mukafukufukuyi ndipo kuti mudzasayinanso pepala lolowera mukafukufukuyi. Ngati magazi a mwana wanu ali ochepa tidzakutumizani kuchipatala kuti akamuthandize moyenelera.

Ubwino ndi zoopsya za kafukufuku
Palibe zoopsya zilizonse pa kufufuza ngati mwana wanu angakhale oyenera kutenga nawo mbali mukafukufukuyi. Dontho la magazi lidzatengedwa pa chala cha mwana wanu ndipo adzatenge dontho la magazili ndi adokotala odziwa bwino ntchito yawo. Mwana wanu adzamva kupweteka pa kanthawi komwe adzatengedwa magaziwo basi. Koma adokotalawo adzawonetsetsa kuti agwira ntchito yawo mwaukadaulo komanso mopanda kupweteka mwana wanu. Tidzakuwuzani zotsatira za kuchuluka kwa magazi a mwana wanu ndipo tikuwuzaninso zomwe mungachite ngati titamupeza kuti mwana wanu ali ndi magazi osakwanira.

Chitetezo cha zokhuza mwana wanu
Zonse zimene mutiuze komanso zotsatira za zimene tiyeze kwa mwana wanu palibe uyo adzadziwe ndipo ofufuza adzaonetsetsa kuti zatetezedwa koposa moti palibenso amene angazitenge komanso angadzadziwe.

Tsogolo la zinthu zomwe tiyese komanso zomwe mutiuze
Zomwe mutiuze apa zitithandiza kuti tichite chiganizo ngati mwana wanu ali oyenera kutenga nawo mbali mukafukufukuyi kapena ayi. Ndipo izi sizidzagwiritsidwanso ntchito ina mtsogolomu kupatula imeneyi basi.

Ufulu otenga nawo mbali kapena kusiya kafukufukuyi
Dziwani kuti kutenga mbali mukafukufukuyi ndi kosakakamiza ayi. Muli ndi ufulu kukana kuti mwana wanu asatenge nawo mbali. Pamene mwasankha kuti mwana wanu akhale m'modzi mwa ana amene angatenge nawo mbali mukafukufukuyi, mulinso ndi ufulu kutuluka mukafukufuku pamene mwafuna. Chiganizo chanu sichisokoneza chisamaliro chomwe mwana wanu kapena inu mungafune kulandira kuchipatala china chilichonse.

Zolipira
Palibe ndalama ina iliyonse yomwe mulandire potenga nawo mbali pamene tikufufuza ngati mwana wanu ali woyenera kutenga nawo mbali mukafukufukuyi.

Ngati muli ndi mafunso komanso amene angayankhe
Ife tikuyenera kukuwuzani chilichonse chokhudzana ndi umoyo wa mwana wanu ndipo tidzakhala okondwa kuyankha funso lina lililonse lomwe mungakhale nalo lokhudzana ndi kafukufukuyi. Muli ololedwanso kuyankhula ndi omwe akuchita kafukufukuyi ngati mungakondwe kutero, manambala awo alembedwa m'musimu.

Mukhonza kufunsa funso lililonse lokhudzana ndi kafukufukuyi komanso ufulu wanu. Ngati mungakhale ndi mafunso ena mwina nthawi ina mukhonza kufunsa pa manambala awa:

Chikwawa Boma: 0995 835 215 or 0883 536 917

IRMA Study

Mfera: 0990 676 012 or 0881 861 069

Mapelera: 0990 676 014 or 0881 861 169

Makwhira: 0994 952 510 or 0883 536 925

Ngati mukufuna kudziwa zambiri zokhudza ufulu wanu otenga nawo mbali mukafukufukuyi, mukhonza kuyimba foni ku bungwe lowona za kafukufuku ku unduna wa za umoyo la National Health Science Research Commitee (NHSRC) polemba kalata ku: cmitambo@gmail.com kapena mohdoccentre@gmail.com. Komanaso mukhonza kuyimba foni ku 0999 397 913.

Ngati mwagwirizana ndi kupempha kwathu kuti inu ndi mwana wanu achite nawo kafukufukuyi chonde onetsani ichi pakusayinira kapena kudinda chala chanu chachikulu chakumanzere m'musimu.

Zikomo chifukwa cha nthawi yanu

____
Dzina la kholo kapena womuyang'anira mwana

______________________________ ________________
Saini la kholo kapena womuyang'anira mwana Tsiku

____
Dzina la mboni (*ngati kholo kapena womuyang'anira mwana sadziwa kulemba kapena kuwerenga*)

______________________________ ________________
Saini la mboni Tsiku

____
Dzina la woyang'anira kafukufuku

______________________________ ________________
Saini ya woyang'anira kafukufuku Tsiku

Mwangi MN, *et al. BMJ Open* 2023; 13:e069011. doi: 10.1136/bmjopen-2022-069011

Training and Research Unit of Excellence (TRUE), Department of Public Health, College of Medicine, Malawi

Walter and Eliza Hall Institute (WEHI), University of Melbourne, Victoria, Australia

2. INFORMED CONSENT FORM – ENROLMENT

Protocol No. 2213	Version No. 4.0	Date: July 25, 2022

Project title: Benefits and safety of IRon supplementation with MAlaria chemoprevention to children in Malawi (IRMA) - A randomised controlled trial

Principal Investigators: Prof Kamija Phiri and Dr Sant-Rayn Pasricha
Organization: Training and Research Unit of Excellence (TRUE), Department of Public Health, College of Medicine, Blantyre, Malawi in collaboration with Walter and Eliza Hall Institute of Medical Research, Australia

This is an invitation for the child in your care to take part in this research project. Ask questions about anything that you don't understand or want to know more about. Before deciding whether or not the child can take part, you might want to talk about it with a relative, friend or other person.

Purpose of the research
As you may know many children in Malawi and elsewhere in the world are anaemic. Many organisations including the Government of Malawi recommend giving all children iron supplementation or multiple micronutrients to prevent anaemia, and these are both currently made and available in Malawi. However, we do not yet know whether giving iron supplements or Multiple Micronutrient Powders are a good idea for improving overall child health, and which one of these is better. In this project, we are planning to see the effect of these treatments on development, growth and health of young children. We also will find out whether giving routine antimalarials to children helps their growth and development. The results of this study will help governments and health policymakers decide if all children should be given these interventions in the future.

Why you are invited to participate in the study?
We have selected Chikwawa district for this study based on available national data on anaemia. Your child lives in the selected area, and has fulfilled all the study eligibility criteria. Hence, we are inviting you to allow your child to take part in this study.

Methods and procedures
What does participation in this research involve?
You will bring your child to our local clinic 3 times; 1) at beginning (today), 2) after 6 months i.e. at the end of receiving the treatment and 3) after 12 months from the beginning of the study i.e. for final tests. At the 1st occasion, a trained tester will take details about socio-economic status of your family, type of toys and stimulation activities your child receives at home, your child's temperament and your feelings towards your life and she will also test your child's brain development by playing with

him/her with some toys. She will also measure yours and your child's height/length, weight, Mid Upper Arm Circumference and child's head circumference. At the end of the interview, a trained technician will collect 3 ml of venous blood (a little less than 1 teaspoon) from your child. On the following 2 clinic visits, all the measurements will be repeated except the demographic and socio-economic status questions. Together, these procedures will take about 2 to 3 hours. We will collect a stool sample from all enrolled children at the at the end of the treatments, and at the final visit.

We would also like to invite you for a long-term follow-up beyond 12 months in future, which depends on availability of funds and would like to seek your consent for preserving and using the left-over samples for future research, and in contacting you for further information in future.

Home visit
When your child is enrolled, one of our research assistants will visit your child every 2 weeks for six months (when s/he is receiving the medicines) and every month for the next 6 months. She/he will record previous weeks' dose taken, bring back the unused quantities and supply new doses for the following 2 weeks. She/he will also collect information on child's morbidity (fever/infection/any other illness), visit to a doctor or hospitalization occurred in previous 2 weeks during active intervention period.

What does the child have to do?
Your child will be participating in a double-blind randomised controlled research project. We wish to compare antimalarial with Dihydroartemisinin-Piperaquine (DP), with or without iron supplements or micronutrient powders, and placebo which is an inert substance. Your child has a one in four chance of receiving either DP, DP with iron supplements (as iron syrup), DP with multiple micronutrient powders, or placebo. However double-blind means that neither you nor the study doctors and investigators will know which of the treatments the child is receiving. After the initial measurements, we will assign your child to one of the four treatments. Your child will be provided with two formulations: a bottle of syrup, and a box of sachets containing powders. During the study, your child will be asked to take 1.3 ml of syrup and one sachet of powders every day for six months. The drops can be given with a graduated dropper or syringe. The powders should be sprinkled on your child's food once it has cooled down a little. Syrup may contain iron or placebo, and sachet may contain multiple micronutrient powder or placebo powder. 1.3 ml of Iron syrup will contain 10mg elemental iron and each MNP sachet will contain 10mg iron, as well as a complement of other nutrients. Once a month, we will also provide Dihydroartemisinin-piperaquine (DP), (or its placebo) which is given over three days. Dihydroartemisinin-piperaquine (DP), is one of the artemisinin containing combination therapies (ACTs). ACTs are now the standard for treatment of *P. falciparum* malaria in both adults and children. A placebo syrup or sachet is a medication with no active ingredients. It looks like the real thing but is not. Once your child is assigned to a group, s/he will be receiving this agent for 6 months.

If your child ever becomes sick or unwell, you can either take them to the clinic, doctor or hospital, as convenient. We will ask about any illness your child may have during our weekly visits.

Right not to participate and withdraw

Does the child have to take part in this research project?
Participation in this research project is voluntary. If you do not wish for the child to take part, they do not have to. If you decide that they can take part and later change your mind, you are free to withdraw the child from the project at any stage. If you do decide that the child can take part, you will be given this Participant Information and Consent Form to sign and you will be given a copy to keep. However, your child's participation in this study would be greatly valuable.

What if I withdraw the child from this research project?
If you do withdraw the child during the research project, the study doctor and relevant study staff will not collect additional personal information, although personal information already collected will be retained to ensure that the results of the research project can be measured properly. You should be aware that data collected by the sponsor up to the time of withdrawal will form part of the research project results. However, withdrawing from the study will involve no penalty and your service from the health facilities will not be withheld, nor will it hamper relationship with those treating him/her, or their relationship with the institutions (College of Medicine and Walter and Eliza Hall Institute).

What are the alternatives to participation?
If you wish, you may decline involvement in the research project and seek attention for anaemia and nutritional status from elsewhere.

Risk and benefits
What are the possible risks and disadvantages of taking part?
Iron supplements and micronutrient powders have been already given to many thousands or millions of children, and are considered safe. However, we will be looking for possible side effects. Many side effects go away shortly after treatment ends. If your child has any of these side effects, or you are worried about them, you can easily talk with us. However, sometimes side effects can be serious, long lasting or permanent. If a severe side effect or reaction occurs, we may need to stop the child's treatment. The child's researcher will discuss the best way of managing any side effects with you. Usually there is no need to stop the supplements.

Side Effect	How often is it likely to occur?	How severe might it be?	How long might it last?
Stomach aches, nausea, constipation or diarrhoea	Perhaps up to 1 in 4 children.	Mild	A few days or the duration of the treatment; but it is usually tolerable
Infection (e.g., diarrhoea)	As often as in children not receiving iron, or perhaps slightly more commonly	About as often as in children not receiving iron, or perhaps slightly more severe.	A few days.
Darkening of stools – this is not a harmful effect at all, and will not harm the child	Common	This is not severe.	For the duration of treatment.

During collection of blood samples slight pricking pain, swelling or bluish colour on the pricking point might be experienced but these resolve quickly. Our experienced technician will ensure complete hygienic precautions and minimum distress.

What are the possible benefits of taking part?
We cannot guarantee or promise that the child will receive any benefits from this research; however, possible benefits may include treatment of anaemia if your child received iron, with improved energy levels, strength and wellbeing in your child, improved mental development, and improved growth. We will share your child's Hb level with you. If your child is severely anaemic or suffers from significant developmental disability diagnosed during brain development tests, we will refer your child to the appropriate health care centres during the study period. Your child's involvement will help us understand how anaemia should best be treated, and so it will have an impact on how we treat children all over the world. If you are invited to come to the research facility, you will be compensated for cost of travel.

Privacy, anonymity and confidentiality
What will happen to information about the child?
By signing the consent form you consent to the study doctor and relevant research staff collecting and using personal information about the child for the research project. Any information obtained in connection with this research project or in any publication and/or presentation, information will be provided in such a way that the child cannot be identified. The data will be kept securely at Training and Research Unit of Excellence and will be shared with The University of Melbourne in Australia through a password protected file. Only researchers on this project will have access to the data. The data will be used for this project and for related projects in the future. In line with data storage requirements for studies involving children, we will retain the data for 25 years and then erase it. The child's information will only be used for the purpose of this research project and it will only be disclosed with your permission, except as required by law.

We will seek to find out the diagnosis of any visit your child makes to a health centre or hospital during the study. By signing the consent form, you agree to the study team accessing health records if they are relevant to the child's participation in this research project.

Information obtained during the research project including your child's health records if accessed as above, are subject to inspection (for the purpose of verifying the procedures and the data) authorised representatives of the Sponsor, Walter and Eliza Hall Institute of Medical Research, the College of Medicine, or as required by law. By signing the Consent Form, you authorise release of, or access to, this confidential information to the relevant study personnel and regulatory authorities as noted above.

Future use of information
What will happen to the child's test samples?
You will be asked to provide consent for the collection of the child's blood during the research project. Collection of blood is the most essential part of involvement in this project.

Samples will be analysed for iron levels in the blood, so we can see if the interventions have shown an effect. We will also store leftover samples in the College of Medicine,

Blantyre Campus, for 5 years from the end date of the study. Samples will be stored in such a way that identity of your child is kept confidential by use of a code. We may measure other markers in the blood samples that relate to the current project (your child's health, nutrition, growth and development) in your child's sample in the future. If samples cannot be tested in Malawi, we may send them overseas for testing, after which they will be destroyed.

Genetic testing:
We will collect samples for future testing of DNA. We will not be testing for genetic diseases, and so the results will not have implications for your child's or the rest of your family's health or diagnosing a genetic disease in the future. Instead, we anticipate testing for genetic differences which may be linked with anaemia, nutrition, development, health and growth, or infection status. Samples and results of genetic testing will not be released to anyone other than the researchers or their colleagues helping with analysis of the samples. If samples cannot be tested in Malawi, we may send them overseas for testing, after which they will be destroyed.

What if new information arises during this research project?
Sometimes during the course of a research project, new information becomes available about the treatment that is being studied. If this happens, the study doctor will tell you about it and discuss with you whether you want your child to continue in the research project. If you decide to withdraw your child from the study, the study doctor will make arrangements for their regular health care to continue. If you decide that the participant can continue in the research project, you will be asked to sign an updated consent form.

Can the child have other treatments during this research project?
Whilst the child is participating in this research project, we discourage you from giving your child other iron formulations because it may mean your child is getting a double dose, and it will make the results of the study difficult to interpret. Please tell us if your child takes or is going to take iron.

Could this research project be stopped unexpectedly?
Yes. This research project may be stopped unexpectedly for a variety of reasons such as lack of funds or unacceptable side effects.

What happens when the research project ends?
At the end of the study, we will test your child for anaemia. If your child is anaemic, we will refer your child to the local health centre for treatment. Once we complete the study and have prepared the final reports, we will provide information on the results of the project to local health workers and facilities.

Principle of compensation
Complaints and Compensation
If you have any complaints or you want to know more about your rights as a research participant you can contact the NHSRC Director at the Ministry of Health in Lilongwe, Phone: +265 999 397 913 Email: cmitambo@gmail.com or mohdoccentre@gmail.com.

If your child suffers any injuries or complications as a result of this research project, you should contact the study team as soon as possible and you will be assisted with arranging appropriate medical treatment.

Compensation
You will be reimbursed, in Malawi Kwacha, the equivalent of 10 USD for each scheduled health facility visit for the time taken to participate in the study, inclusive of any travel costs.

Answering your questions/ Contact persons
Who is organising and funding the research?
This research project is being conducted by Professor Kamija Phiri of the College of Medicine and Dr Sant-Rayn Pasricha of Walter and Eliza Hall Institute of Medical Research.

Who has reviewed the research project?
The ethical aspects of this research project have been approved by the National Health Sciences Research Committee (NHSRC) at the Ministry of Health and Health and Research Ethic Committee (HREC) of the Walter and Eliza Hall Institute of Medical Research, Melbourne.

Further information and who to contact
If you want any further information concerning this project or if the participant has any medical problems which may be related to their involvement in the project (for example, any side effects), you can ask us today or during our regular visits or subsequent visits. If you have other questions later, you can contact: Prof Kamija Phiri, Principal Investigator, College of Medicine, Blantyre, 0999 957 048 or Dr Sant-Rayn Pasricha, Principal Investigator from Walter and Eliza Hall Institute of Medical Research Tel: +61393452618

For matters relating to research at the site at which the child is participating, the details of the local site complaints persons are:

Complaints contact numbers

Chikwawa Boma: 0995 835 215 or 0883 536 917

Mfera: 0990 676 012 or 0881 861 069

Mapelera: 0990 676 014 or 0881 861 169

Makwhira: 0994 952 510 or 0883 536 925

If you have any complaints about any aspect of the project, the way it is being conducted or any questions about being a research participant in general, then you may contact:

Reviewing HREC name	**National Health Sciences Research Committee**
HREC Executive Officer	**Dr Collins Mitambo**
Telephone	*0999 397 913*
Email	**mohdoccentre@gmail.com or- cmitambo@gmail.com**

Declaration by Parent/Guardian

By signing the consent form you are agreeing that:

- You have read the consent or someone has read it to you in Chichewa.
- You understand the purposes, procedures and risks of the research described in the project.
- You give permission for the child's doctors, other health professionals, hospitals or laboratories outside this hospital to release information to the College of Medicine and The Walter and Eliza Hall Institute of Medical Research concerning the child's disease and treatment for the purposes of this project. You understand that such information will remain confidential.
- You have had an opportunity to ask questions and you are satisfied with the answers you have received.
- You freely agree to the child participating in this research project as described and understand that you are free to withdraw them at any time during the research project without affecting their future health care.
- You understand that you will be given a signed copy of this document to keep.

____
Name of Parent/ Guardian/

____________________________ _______________
Signature or right thumb impression of Parent/ Guardian/ Attendant — Date

____
Name of Witness (*If Parent/ Guardian cannot read and write*)

____________________________ _______________
Signature of Witness — Date

____
Name of Investigator or study staff

____________________________ _______________
Signature of Investigator or study staff — Date

IRMA Study

Training and Research Unit of Excellence (TRUE), Department of Public Health, College of Medicine, Malawi

University of Melbourne, Victoria, Australia

2. KALATA YOPEMPHA CHILOLEZO

Protocol No. 2213	Version No. 4.0	Date: July 25, 2022

Mutu wa kafukufuku: Kufufuza ubwino ndi chitetezo cha mankhwala okhala ndi ironi komanso mankhwala oteteza ku malungo kwa ana m'Malawi**.**

Oyang'anira kafukufuku: Professor Kamija Phiri ndi Dr Sant-Rayn Pasricha
Bungwe: Training and Research Unit of Excellence (TRUE), Department of Public Health, College of Medicine, Blantyre, Malawi mogwirizana ndi University of Melbourne, Australia

Takuitanani kuti tikupempheni kuti mwana wanu atenge nawo mbali mu kafukufuku amene tikuchita. Muli ololedwa kufunsa mafunso okhudza chilichonse chomwe mukufuna mutadziwa kapena chomwe simukumvetsa pakukambirana kwathu. Musanapange chiganizo chokuti mwana wanu atenge nawo mbali mukafukufuku kapena ayi, mutha kudziwitsa kapena kuuza wachibale, mnzanu kapena munthu wina aliyense za nkhaniyi.

Cholinga cha kafukufuku
Monga mukudziwa, ana ambiri m'Malawi muno ndi kwina konse m'dziko lapansi, amakhala ndi vuto lakuchepekedwa magazi. Mabungwe, kuphatikizapo boma la Malawi amalimbikitsa kuti ana onse alandire iron oonjezera kapena michere yosiyana yosakaniza yoonjezera popewa matenda akuchepa kwa magazi. Iron ndi michere yi zikupangidwa ndi kupezeka kuno ku Malawi. Ngakhale zili chomwechi, pakadali pano sizikudziwika bwinobwino ngati kupereka iron oonjezera kapena michere yosakaniza ndi njira zabwino zopititsa patsogolo umoyo wa ana. Komanso sizikudziwika bwinobwino kuti kodi njira yabwino kwambiri pa njira ziwiri zimenezi ndi iti pothandizira kakulidwe ka ana. Mu kafukufukuyu, tikufuna kufufuza zotsatira za njirazi makamaka pa makulidwe komanso umoyo wa ana. Zotsatira za kafukufukuyu zidzathandiza boma komanso ogwira ntchito za umoyo kupanga chiganizo ngati kuli koyenera kupereka njirazi kwa ana onse mtsogolomu.

N'chifukwa chiyani mwasankhidwa kutenga nawo mbali mu kafukufuku?
Boma la Chikwawa lasankhidwa ndi akuluakulu a Training and Research Unit of Excellence (TRUE) mogwirizana ndi University of Melbourne ku Australia potengera zomwe tikudziwa kale zokhudzana ndi kukula kwa vuto lakuchepa magazi kuno ku Malawi. Mwana wanu amakhala mu dera lomwe lasankhidwali ndipo ali ndi zonse zomuyenereza kutenga nawo mbali mu kafukufukuyu. Pa chifukwa ichi, tikukupempthani kuti mwana wanu atenge nawo mbali mu kafukufukuyu.

Njira zomwe zidzagwiritsidwe ntchito kapena kutsatidwa
Kodi muyenera kuchita chiyani pamene mwatenga mbali mu kafukufukuyu

Mwangi MN, *et al. BMJ Open* 2023; 13:e069011. doi: 10.1136/bmjopen-2022-069011

Mudzayenera kubwera ndi mwana wanu ku chipatala kuno katatu; 1) tsiku loyamba (lero), 2) patatha miyezi isanu ndi umodzi (6) kutanthauza kuti ku mapeto kolandira mankhwala, 3) patatha miyezi khumi ndi iwiri chiyambireni kafukufukuyu

Pa tsiku loyamba, a kafukufuku adzakufunsani mafunso angapo makamaka okhudzana ndi pakhomo panu, zinthu zomwe mwana wanu amaseweretsa pakhomo, makhalidwe ake atsiku ndi tsiku komanso mmene inu mumaonera za umoyo wanu. Adzayesanso mmene mwana wanu akukulira m'maganizidwe ake ndipo adzachita izi posewera naye. Adzayesanso msinkhu wanu ndi wa mwana, kulemera komanso kanenepedwe ka mkono komanso mutu wa mwana wanu. Pamapeto pa mafunso, adzakupemphani kuti atengeko magazi a mu mtsempha wa pa mkono okwanira 3ml (osakwanira pa supuni yaing'ono). Pa nthawi ya chiwiri yomwe mudzabwerenso, patatha miyezi isanu ndi umodzi (6), zonse zomwe munayesedwa zidzayesedwanso kupatula mafunso okhudza inu ndi za mwana wanu. Kuchita zimenezi zidzatenga maola awiri kapena atatu kuti timalize. Tidzatenganso chimbudzi cha ana onse omwe adzalowe mu kafukufukuyu pa nthawi imene akubwera ku chipatala kuno komaliza.

Tikufuna kukuitananinso mu kalondolondo yemwe adzachitike patatha miyezi khumi ndi iwiri. Dziwani kuti kalondolondo ameneyu adzatengera mmene za chuma zidzakhalire. Komanso tikufuna kukupemphani ngati mungatilore kusunga zonse zotsala kuti tidzathe kugwiritsanso ntchito mukafukufuku, komanso kulumikizana nanu pa nkhani ya kafukufuku mtsogolomu.

Kuyendera makomo

Pamene mwana wanu walowa mu kafukufukuyu, m'modzi mwa othandizira mu kafukufuku adzayendera mwana wanu masabata awiri aliwonse kwa miyezi isanu ndi umodzi (6) pamene akulandira mankhwala komanso mwezi uliwonse kwa miyezi isanu ndi umodzi yotsatira. Adzalemba mu mabuku awo mankhwala amene mwana wamwa mu mmasabata awiri a mmbuyomu, kutenga mankhwala omwe sanagwiritsidwe ntchito ndi kupereka mankhwala oti mwana wanu amwe mu masabata otsatira. Adzakufunsinanso mafunso a umoyo wa mwana wanu monga ngati mwana wanu anatentha thupi, kapena munamutengera ku chipatala kukaonana ndi adokotala kapena kugonekedwa mu chipatala mu masabata awiri angothawo.

Kodi mwana ayenera kuchita chiyani mukafukufukuyi?

Mu kafukufukuyu, inu komanso opereka makhwala sadzadziwa kuti mwana wanu, potenga nawo mbali, ali gulu liti ndipo akulandira mankhwala anji pa magulu anayi amankhwala omwe akhazikitsidwa. Magulu anayi amankhwalawa ndi 1) makhwala oteteza ku malungo ndi ironi wa madzi, 2) mankhwala oteteza ku malungo ndi iron wa ufa, 3) mankhwala oteteza ku malungo ndi 4) mankhwala ena ongofanizira. Mwana wanu adzakhala mugulu limodzi la magulu mwamagulu anayi amenewa. Iye adzapatsidwa mankhwala a mitundu itatu; botolo la mankhwala a madzi, kaphukusi ka timasacheti a mankhwala a ufa ndi mankhwala a malungo. Nthawi ya kafukufuku, adzidzamwa supuni imodzi yothilira shuga ya mankhwala a madzi ndi kaphukusi kamodzi ka mankhwala a ufa tsiku lililonse kwa miyezi isanu ndi umodzi. Mutha kupereka ma dontho a mankhwala a madzi pogwiritsa ntchito kachubu kolembedwa milingo. Mankhwala a ufa atha kuperekedwa pothira mankhwalawo ku chakudya cha

mwana chomwe chazizira pang'ono. Mankhwala a madzi atha kukhala ndi iron kapena izo zongofanizira, ndipo mankhwala a ufa atha kukhala ndi michere kapena mankhwala ongofanizira a ufa. Mulingo wa mankhwala a madzi wa 1.3 mililita yomwe muzampatsa mwana wanu ndiwolingana ndi mamiligalamuzi 10 a iron ndipo sacheti imodzi ya mankhwala a ufa ndiyolingana ndi mamiligalamu 10 a iron, mamiligalamu 0.3 a vitamin A, mamiligalamu 30 a vitamin C, mamiligalamu 0.16 a foliki asidi ndi mamiligalamu 5 a zinki). Mankhwala omwe adzakhale opanda chilichonse nkati mwake adzaoneka ngati mankhwala ena amene mwana aliyense adzalandire. Mankhwala ofanizira sangagwire ntchito ina iliyonse mnthupi ngakhale amaoneka ngati mankhwala eni eni. Pamene mwana wanu waikidwa mu gulu, adzalandira mankhwala ngati amenewa kwa miyezi isanu ndi umodzi (6).

Ngati mwana wanu wadwala, mumutengere ku chipatala kuti akalandire thandizo ndipo tidzafunsa ngati mwana anadwala pa nthawi imene tizidzayendera makomo masabata awiri aliwonse.

Ufulu otenga nawo mbali komanso kutuluka mu kafukufuku
Kodi mwana akuyenera kutenga mbali mu kafukufukuyu?
Kutenga nawo mbali mu kafukufukuyu ndikosakakamiza kwa wina aliyense. Ngati simukufuna kuti mwana wanu atenge nawo mbali, muli ololedwa kutero. Ngati mwavomera kuti mwana wanu atenge nawo mbali mu kafukufukuyu kenako mwasintha maganizo pakati pa kafukufukuyu, muli ololedwa kumutulutsa mwana wanu nthawi ina iliyonse imene mwaganiza kutero. Ngati mwavomera kuti mwana wanu atenge mbali, mudzapatsidwa kalata ya mtengambali komanso kalata yopempha chilolezo yomwe mudzasainira ndipo mudzapatsidwa kalata imodzi kuti musunge.

Chingachitike ndi chiyani nditamutulutsa mwana mu kafukufukuyu?
Ngati mutaganiza zomutulutsa mwana wanu pakati pa kafukufuku, a dotolo ndi ena onse ochita kafukufukuyu sadzatenganso chilichonse chokhudza inu kapena mwana wanu. Dziwani kuti zonse zomwe tinatolera kale zidzasungidwa ndipo zidzakhala nawo mbali imodzi ya ma lipoti a kafukufukuyi. Dziwaninso kuti, kutuluka mukafukufukuyi simudzalandira chilango china chilichonse ndipo sizidzakhudza chithandizo chomwe mumalandira ku chipatala kapena kuononga ubale wanu ndi madotolo anu, kapenanso ubale wawo ndi masukulu a ukachenjede omwe akuchita kafukufukuyu.

Kodi pali njira inanso yotengera nawo mbali mu kafukufukuyu?
Dziwani kuti monga tinanenela muja kuti kutenga nawo mbali mukafukufukuyi sizokakamiza. Ngati mwasankha kusatenga nawo mbali mu kafukufukuyu mutha kukapeza thandizo la vuto la kuperewera kwa magazi ndi za thanzi kwina kulikonse komwe inu mungafune.

Zoopsya zomwe zingachitike
Kodi zoopsya zomwe zingachitike potenga mbali mukafufufukuyu ndi chiyani?
Iron oonjezera komanso ufa wa michere zakhala zikupatsidwa kwa ana miyandamiyanda ndipo ndi osaopsya. Ngakhale zili chonchi, tikhala tikufufuza ngati pali zina zoopsya zomwe zingabwere chifukwa cha mankhwalawa. Ngati zingachitike zina zoopsya sizitenga nthawi yambiri zisanathe kuchokera pa nthawi yomwe

Mwangi MN, *et al. BMJ Open* 2023; 13:e069011. doi: 10.1136/bmjopen-2022-069011

mankhwala asiya kuperekedwa. Ngati mwana wanu akuonetsa zizindikiro zosakhala bwino kapena mukudandaula za iye, chonde tiuzeni. Ngakhale zili chonchi, zina zotsatira mankhwalawa zitha kukhala zoopsya, zokhala nthawi yaitali zisanathe komanso zokhalitsa mpaka kale. Ngati zotsatira zoopsya zaoneka, tidzasiya kumupatsa mwana mankhwalawa. Amene akuchita kafukufuku pa mwanayu, adzayenera kupereka upangiri oyenera wothana ndi vutolo ndi inu. Koma nthawi zambiri, palibe icho choopsya chomwe chinaoneka kwa ana omwe akulandira iron komanso michere imene tatchula ija.

Zotsatira zosayembekezeleka	Zimachitika pafupipafupi?	Ndi zoopsya motani	Zingakhale nthawi yaitali bwanji zisanathe?
Kupweteka kwa m'mimba, chilungulira, kudzimbidwa kapena kutsekula m'mimba	Mwina m'modzi mwa ana anayi	Ndizosaopsya kwambiri	Masiku ochepa kapena nthawi yomwe akulandira chithandizo; koma zopililika
Matenda (monga kutsekula m'mimba)	Izi zifanana ndi m'mene zingachitikire mwa ana amene sakulandira iron kapena zitha kuchitikachitika	Ngati mene zingachitikire mwa ana amene sakulandira iron kapena zitha kuchitika moopsa pang'ono	Masiku ochepa
Kuda kwa chimbudzi – izi sizotsatira zoopsa ndipo sizoopsa kwa mwana	Zimachitikachitika	Sizoopsya ndipo musadandaule nazo	Nthawi yomwe akulandira mankhwala.

Nthawi yotenga magazi munthu atha kumva kupweteka kobaya pang'ono, kutupa kapena kusintha mtundu pa malo omwe abaidwa. Madotolo athu odziwa bwino ntchito adzaonetsetsa kuti pali ukhondo komanso kuchepetsa ululu.

Kodi phindu lotenga nawo mbali mu kafukufukuyu ndi lotani?

Sitinganene mwandithendithe kuti mwana adzapeza phindu lililonse mu kafukufukuyu; ngakhale zili chonchi, zina mwa izo zomwe mwana angapindule nazo ndizo; kuthandizidwa ku vuto lakuchepa kwa magazi ngati mwana wanu walandira iron, kukhala wa mphamvu ndi nyonga, kukhala ndi umoyo wabwino, kukula mu nzeru ndi msinkhu. Tidzakupatsani zotsatira za magazi a mwana makamaka kuchuluka kwa iron. Ngati vuto lochepa kwa magazi lili lalikulu kapena mwana wanu sakukula bwino, tidzamutumiza mwana wanu ku chipatala choyenelera kuti akalandire thandizo pa nthawi ya kafukufukuyu. Kutenga nawo mbali kwa mwana wanu kudzatithandiza kuti tipeze njira za bwino zothana ndi vuto lakuchepa kwa magazi ndipo zidzathandiza mmene tingathandizire ana ena onse dziko lonse lapansi. Dziwaninso kuti pamene mwabwera kuchipatala pa nthawi yakafukufukuyi tidzakubwezerani ndalama yanu yoyendera yolingana ndi 10 USD.

Mwangi MN, *et al. BMJ Open* 2023; 13:e069011. doi: 10.1136/bmjopen-2022-069011

Kusunga chinsinsi

Kodi uthenga wa mwana omwe mudzatenge udzagwiritsidwa ntchito motani?
Pamene mwasaina kalata yopereka chilolezo, ndiye kuti mukupereka chilolezo kwa dotolo komanso onse amene akuthandizira mu kafukufukuyu kutenga ndi kugwiritsa ntchito uthenga okhudza mwana wanu mu kafukufukuyu basi. Uthenga uliwonse omwe udazatengedwe okhudza mwana wanu mogwirizana ndi kafukufukuyu, pamene tikusindikiza, kapena kugawidwa kwa ena, udzaperekedwa mu njira yokuti palibe amene adzaziwe dzina la mwana wanu kapena kulondoloza mwana wanu. Uthengawo udzasungidwa bwino ku bungwe lomwe likuyendetsa za kafukufukuyi komanso udzagawidwa kwa a kadaulo a sukulu ya ukachenjede a ku Australia kudzera mu uthenga wa pa internet omwe udzakhale wotetezedwa. Uthengawu udzagwiritsidwa ntchito mu kafukufukuyu komanso kafukufuku wina ofanana naye kutsogoloko. Molingana ndi ndondomeko ya kasungidwe ka uthenga wa kafukufuku okhudza ana, tidzasunga uthengawu kwa zaka 25 ndipo kenaka udzafutidwa. Uthenga wa mwana udzagwiritsidwa ntchito pa zolinga za kafukufukuyu ndipo sudzaulutsidwa pokhapokha ngati inu mwavomereza ndi kupereka chilolezo, kupatula pamene uthengawu ukufunidwa molingana ndi lamulo.

Pa nthawi imene kafukufuku akuchitika, tidzafuna kudziwa nthenda kapena vuto linalililonse lomwe mwana angapezeke nalo pamene wapita ku chipatala kukapimidwa. Pamene mwasaina kalata ya chilolezo ndiye kuti mukugwirizana ndi zokuti onse amene akuthandizira mu kafukufukuyu akhoza kuona chiphaos cha umoyo cha mwana kuti afufuze ngati umoyo wa mwanawo ukumuyenereza kutenga nawo mbali mu kafukufukuyu.

Uthenga omwe udzatengedwa nthawi yakafukufuku kuphatikizapo omwe udzatengetengedwe mu chiphaso cha umoyo wa mwana zidzafufuzidwa mokwana ndi cholinga chofuna kuona ngati pali mgwirizano pakati pa njira zomwe zidzatsatidwe mu kafukufukuyu ndi uthenga omwe watengedwa. Izi zidzachitidwa ndi akuluakulu a kafukufuku amene ali ololedwa kutero. Pamene mwasaina kalata ya chilolezo, ndiye kuti mwagwirizana ndi zakuti mudzalola kuonetsa kapena kupereka uthenga oyenera kwa onse amene akugwira ntchito mu kafukufuku.

Kugwiritsa ntchito uthenga mtsogolo
Kodi magazi kapena chimbuzi chomwe chidzatengedwa zidzagwira ntchito yanji?
Mukuyenera kudzapereka chilolezo pa nthawi ya kafukufuku kuti mwana wanu adzatengedwe magazi. Kutenga magazi ndi mbali imodzi yofunika kwambiri mu kafukufukuyu.

Magazi amene adzatengedwa adzaunikidwa kuti tione kuchuluka kwa iron ndi cholinga chakuti, pa mapeto a kafukufukuyu tidzaone ngati mankhwala omwe mwana amalandira agwira ntchito kapena ayi. Tidzasunganso magazi otsala ku sukulu ya ukachenjede yomwe ikutsogolera kafukufukuyu ku Blantyre kwa zaka zisanu kuchokera tsiku lomaliza la kafukufuku. Ma sampulo a magazi adzasungidwa kuti wina aliyense asadzazindikire mwana wanu pogwiritsa ntchito nambala ya chinsinsi.

Tidzatha kuyesanso zina mu sampulo ya magazi otsala mu kafukufuku wina amene angafanane ndi amene tikupangayi mtsogolomu.

Kuyesa ma jinzi

Tidzatenganso magazi kuti tikayese DNA wa mwana wanu koma izi zidzachitika mtsogolomu. Komabe, sitidzayesa matenda ochokera ku mtundu, kotero zotsatira za kuyesa kumeneku sikudzabweretsa nkhawa iliyonse kwa mwana komanso banja lonu ndipo palibenso kuthekera kulikonse koyesa matenda a ku kumtundu mu sampulo ya magazi imeneyi. M'malo mwake, tikuyembekezera kudzayesa magaziwa kuti tidziwe ngati pali mgwirizano ulionse pakati pa zotengera za ku mtundu ndi vuto la kusowa kwa magazi, nthanzi, makulidwe, kapena matenda. Magazi ndi zotsatira zake, sizidzaperekedwa kwa wina aliyense kupatula amene akugwira ntchito mu kafukufukuyu. Ngati zipangizo zoyezela zidzavute kwathu kuno, ndiye kuti magaziwa adzatumizidwa ku maiko a kunja ndipo pamapeto ake tidzakawataya moyenelera.

Nanga ngati patapezeka uthenga wina pamene kafukufukuyu akuchitika?
Nthawi zina, pakati pa kafukufuku, pamatha kubwera uthenga wina wa chilendo okhudza mankhwala amene akufufuzidwa. Ngati izi zitachitika, dokotala akuyenera kukufotokozerani ndipo muli ndi ufulu osankha kupitiliza kapena kumutulutsa mwana wanu mu kafukufukuyu. Ngati mwaganiza zomutulutsa mwana wanu, a dotolo amene akutenga mbali mu kafukufuku adzapanga chikonzero chakuti mwana wanu apitirize kulandira thandizo linalonse la ku chipatala moyenera. Mgati mudzaganiza zopitiliza kutenga nawo mbali, mudzayenera kusaina kalata ina yachilolezo yokonzedwa molingana ndi kusinthako.

Kodi mwana akhonza kulandira mankhwala ena a iron mu nthawi ya yakafukufukuyu?
Pa nthawi imene mwana akutenga nawo mbali mu kafukufukuyu, timalimbikitsa kuti mwana asalandirenso iron wina kupatula yekhayo amene ife tikupereka chifukwa ngati mutero, ndiye kuti mwana wanu akulandira iron kawiri zimenene zitha kukhala zoopsa kwa mwana wanu komanso zingasokoneze zotsatira za kafukufuku. Chonde, tidziwitseni ngati mwana akulandira iron wina kupatula amene tikupatseniyi.

Kodi kafukufukuyu akhonza kuimitsidwa mwadzidzidzi?
Eya. Kafukufukuyu akhonza kuimitsidwa mosayembekezereka pa zifukwa zosiyanasiyana monga kusowa kwa thandizo la ndalama loyendetsera kafukufuku, kapena zizindikiro zoopsya zomwe zingabwere chifukwa cholandira mankhwala.

Chidzachitike ndi chiyani pamene kafukufuku wafika pamapeto?
Pa mapeto a kafukufukuyu tidzayesa mwana wanu kuti tione ngati ali ndi vuto lakuchepa kwa magazi kapena ayi. Ngati mwana wanu adzapezeke ndi vutoli, tidzamutumiza ku chipatala kuti akalandire thandizo. Tikadzamaliza kafukufuku ndi kulemba lipoti lomaliza, tidzapereka zotsatira za kafukufukuyu kwa alangizi a za umoyo ndi m'zipatala.

Madandaulo ndi chipukuta misonzi
Ngati muli ndi dandaulo lokhudza kafukufukuyu kapena mukufuna kudziwa zambiri za kafukufukuyi mukhonza kudziwitsa Mkulu woyang'anira bungwe lomwe limaona za

Mwangi MN, *et al. BMJ Open* 2023; 13:e069011. doi: 10.1136/bmjopen-2022-069011

madandaulo a anthu omwe akutenga nawo mbali mu kafukufuku la National Health Sciences Research Committee (NHSRC) limene lili ku unduna wa za umoyo wa boma la Malawi ku Lilongwe, poyimba foni pa: 0999 397 913 komanso uthenga wodzera pa intaneti polembela ku: cmitambo@gmail.com kapena mohdoccentre@gmail.com.

Ngati amene akutenga nawo mbali wavulala kapena wapezeka ndi vuto lomwe ladza kamba ka kafukufukuyu, mukuyenera kudziwitsa ogwira ntchito mu kafukufukuyu mwa msanga, ndipo iwo adzakuthandizani pokonza chithandizo choyenera cha mankhwala.

Chipukuta misonzi
Mudzabwezeredwa ndalama m'makwacha yofanana mphamvu ndi 10 USD ya nthawi imene mwagwiritsa ntchito potenga nawo gawo mu kafukufukuyu kuphatikiza ndalama ya mayendedwe pa nthawi yomwe mwapemphedwa kubwera kuchipatala.

Kuyankha mafunso/ anthu omwe mungalumikizane nawo
Kodi amene akonza ndi kupereka thandizo la ndalama mu kafukufukuyu ndi ndani? Amene akuchita kafukufukuyu ndi nkulu wa kafukufuku a Kamija Phiri a ku sukulu ya ukachenjede ya madotolo kuno ku Malawi ndi a Sant-Rayn Pasricha a ku sukulu ya ukachenjede ya Melbourne ku Australia.

Amene apereka chilolezo chochitira kafukufukuyu ndi ndani?
Amene apereka chilolezo chochitira kafukufukuyu ndi mabungwe amene amaona za ufulu wa anthu amene akutenga nawo mbali mu kafukufuku. Mabungwewa ndi la ku Malawi ndipo lina ndi la ku Australia.

Ngati mukufuna kudziwa zambiri ndi amene mungalumikizane nawo
Ngati mukufuna kudziwa zambiri kapena amene watenga nawo mbali mu kafukufukuyu wakumana ndi vuto lomwe ladza kamba ka kafukufukuyu (mwa chitsanzo, zizindikiro zoopsya zomwe zadza chifukwa cholandira mankhwala athu), mutha kufunsa pompano kapena nthawi iliyonse yomwe tidzakuyenderani. Koma ngati mungakhale ndi mafunso nthawi ina iliyonse, mutha kuimba lamya ndi kuyankhulana ndi mkulu wa kafukufukuyu amene akupezeka ku Blantyre, Professor Kamija Phiri pa nambala iyi: 0999 957 048 kapena Dr. Sant-Rayn Pasricha, mkulu wa kafukufuku ku chokera ku Australia, Tel: +61393452618

Pa nkhani zokhudza kafukufuku pa malo kapena chipatala chimene mwana akutengerapo mbali, mutha kupereka madandaulo anu kwa munthu wa chipatalapo kudzera pamanambala awa:

Chikwawa Boma: 0995 835 215 or 0883 536 917

Mfera: 0990 676 012 or 0881 861 069

Mapelera: 0990 676 014 or 0881 861 169

Makwhira: 0994 952 510 or 0883 536 925

Ngati muli ndi madandaulo ena okhudzana ndi kafukufukuyu, mmene akuyendetsedwera, kapena mafunso ena alionse okhudzana ndi otenga mbali, mukhonza kulumikizana ndi:

Reviewing HREC name	**National Health Sciences Research Committee**
HREC Executive Officer	**Dr Collins Mitambo**
Telephone	*0999 397 913*
Email	**cmitambo@gmail.com** or **mohdoccentre@gmail.com**

Kuvomereza kwa oyang'anira mwana

Ngati mupange chisankho chotenga nawo mbali mukafukufukuyu, mudzapemphedwa kuti musayine kalatayi, ndipo kalata ina idzasungidwa ndiife ina ndi inu ngati umboni wakuti mwavomera kutenga nawo mukafukufukuyu. Posayinira kalata ya chilolezoyi mukuvomereza kuti:

- Mwawerenga kapena kuwerengeredwa kalata yopempha chilolezoyi mu Chichewa.
- Mwamvetsetsa cholinga, ndondomeko komanso zovuta zina za kafukufukuyu.
- Mukupereka chilolezo kwa ma dotolo komanso onse a zaumoyo kupereka uthenga uliwonse okhudza umoyo wa mwana wanu ku College of Medicine ndinso University of Melbourne *ndi cholinga chofuna kuthandiza nawo mu kafukufukuyu ndipo muli ndi chitsimikizo kuti uthenga wa umoyo wa mwanawu udzasungidwa mwa chinsinsi.*
- Munapatsidwa mwayi ofunsa mafunso ndipo mwakhutira ndi mayankho omwe mwapatsidwa.
- Mukuvomera mwa ufulu kuti mwana wanu atenge nawo mbali mu kafukufukuyu molingana ndi mmene mwafotokozeredwa ndipo mukudziwa kuti muli ndi ufulu osiya kutenga nawo mbali mu kafukufukuyu nthawi inailiyonse ndipo izi sizidzakhudzana ndi kulandira chithandizo ku chipatala mtsogolomo.
- Mukudziwanso kuti mukuyenera kusunga kalata imodzi yopempha chilolezo yomwe ya sainidwa.

____

Dzina la kholo kapena womuyang'anira mwana

____________________________ _______________

Saini la kholo kapena womuyang'anira mwana Tsiku

____

Dzina la mboni (*ngati kholo kapena womuyang'anira mwana sadziwa kulemba kapena kuwerenga*)

____________________________ _______________

Saini la mboni Tsiku

Mwangi MN, *et al. BMJ Open* 2023; 13:e069011. doi: 10.1136/bmjopen-2022-069011

____

Dzina la woyang'anira kafukufuku

_______________________________________ _____________________

Saini ya woyang'anira kafukufuku Tsiku
