# Supplementary material for: Protocol for a randomised, multicentre, four-arm, double-blinded, placebo-controlled trial to assess the benefits and safety of iron supplementation with malaria chemoprevention to children in Malawi: IRMA trial
Source: BMJ Open. 2023 Oct 13;13(10):e069011. doi: 10.1136/bmjopen-2022-069011 (PMC10583080; doi:10.1136/bmjopen-2022-069011)
Supplement: Author's manuscript [file bmjopen-2022-069011.draft_revisions.pdf]

BMJ Open is committed to open peer review. As part of this commitment we make the peer review history of every article we publish publicly available.

When an article is published we post the peer reviewers' comments and the authors' responses online. We also post the versions of the paper that were used during peer review. These are the versions that the peer review comments apply to.

The versions of the paper that follow are the versions that were submitted during the peer review process. They are not the versions of record or the final published versions. They should not be cited or distributed as the published version of this manuscript.

BMJ Open is an open access journal and the full, final, typeset and author-corrected version of record of the manuscript is available on our site with no access controls, subscription charges or pay-per-view fees (http://bmjopen.bmj.com).

If you have any questions on BMJ Open's open peer review process please email info.bmjopen@bmj.com

BMJ Open

Protocol for a randomised, multicentre, four-arm, double-blinded, placebo-controlled trial to assess the benefits and safety of Iron supplementation with Malaria chemoprevention to children in Malawi - IRMA trial

Journal:	*BMJ Open*
Manuscript ID	bmjopen-2022-069011
Article Type:	Protocol
Date Submitted by the Author:	07-Oct-2022

Complete List of Authors:	Mwangi, Martin; Training and Research Unit of Excellence (TRUE), Nutrition and Infectious Diseases ; The Micronutrient Forum, Healthy Mothers Healthy Babies Consortium<br>Mzembe, Glory; Training and Research Unit of Excellence (TRUE), Nutrition and Infectious Diseases ; Kamuzu University of Health Sciences, Public Health<br>Ngwira, Chikondi ; Training and Research Unit of Excellence (TRUE), Nutrition and Infectious Diseases ; Liverpool School of Tropical Medicine<br>Kapulula, Mayamiko; Training and Research Unit of Excellence (TRUE), Nutrition and Infectious Diseases<br>Vokhiwa, Maclean; Training and Research Unit of Excellence (TRUE), Nutrition and Infectious Diseases<br>Larson, Leila; University of South Carolina Arnold School of Public Health, Department of Health Promotion, Education, and Behaviour<br>Braat, Sabine ; University of Melbourne School of Population and Global Health, Centre for Epidemiology and Biostatistics; Walter and Eliza Hall Institute of Medical Research<br>Harding, Rebecca; The Walter and Eliza Hall Institute of Medical Research,<br>McLean, Alistair; Walter and Eliza Hall Institute of Medical Research; The University of Melbourne, Department of Infectious Diseases<br>Hamadani, Jena; International Centre for Diarrhoeal Disease Research<br>Biggs, Beverley-Ann ; The University of Melbourne, Medicine<br>Ataíde, Ricardo; The Walter and Eliza Hall Institute of Medical Research; The University of Melbourne, Department of Infectious Diseases<br>Phiri, Kamija; Training and Research Unit of Excellence (TRUE), Nutrition and Infectious Diseases ; Kamuzu University of Health Sciences, Public Health<br>Pasricha, Sant-Rayn; Walter and Eliza Hall Institute of Medical Research,

Keywords:	Anaemia < HAEMATOLOGY, Infection control < INFECTIOUS DISEASES, PUBLIC HEALTH, Nutrition < TROPICAL MEDICINE

For peer review only - http://bmjopen.bmj.com/site/about/guidelines.xhtml

Protocol for a randomised, multicentre, four-arm, double-blinded, placebo-controlled trial to assess the benefits and safety of Iron supplementation with Malaria chemoprevention to children in Malawi - IRMA trial

Authors

*Martin N. Mwangi[1,2], *Glory Mzembe[1,3], Chikondi C. Ngwira[1], Mayamiko D. Kapulula[1], Maclean Vokhiwa[1,3], Leila M. Larson[4], Sabine Braat[5,6,7], Rebecca Harding[7], Alistair R.D. McLean[6,7], Jena D. Hamadani[8], Beverley-Ann Biggs[5,9] Ricardo Ataíde[5,7,9], Kamija S. Phiri[1,3], Sant-Rayn Pasricha [7,9,10]

*Joint first authors

Corresponding author:

Martin N. Mwangi, Training and Research Unit of Excellence (TRUE), 1 Kufa Road, Post Office Box 30538, Blantyre, Malawi. Email: mmwangi@true.mw

Author Institutions

1. Training and Research Unit of Excellence (TRUE), Blantyre, Malawi.
2. The Micronutrient Forum, Healthy Mothers Healthy Babies Consortium, Washington DC, USA.
3. Department of Public Health, School of Public and Global Health, Kamuzu University of Health Sciences, Blantyre, Malawi.
4. Department of Health Promotion, Education, and Behaviour, Arnold School of Public Health, University of South Carolina, Columbia, South Carolina, USA.
5. Department of Infectious Diseases, University of Melbourne, at the Peter Doherty Institute for Infection and Immunity, Melbourne, Australia.

For peer review only - http://bmjopen.bmj.com/site/about/guidelines.xhtml

6. Centre for Epidemiology and Biostatistics, Melbourne School of Population and Global Health, The University of Melbourne, Melbourne, Australia.
7. Population Health and Immunity Division, The Walter and Eliza Hall Institute of Medical Research, Melbourne, Australia.
8. International Centre for Diarrhoeal Disease Research, Bangladesh, Dhaka, Bangladesh.
9. Diagnostic Haematology, The Royal Melbourne Hospital; and Clinical Haematology, , Melbourne, Australia.
10. Department of Medical Biology, Faculty of Medicine, Dentistry and Health Sciences, The University of Melbourne, Melbourne, Australia.

Word count: 4303. (Abstract) 337 words. *(excluding title page, abstract, references, figures and tables)*

For peer review only - http://bmjopen.bmj.com/site/about/guidelines.xhtml

ABSTRACT

Introduction: Approximately 40% of children 6-59 months of age worldwide are anaemic. Iron-containing multiple micronutrient powders (MNPs) and iron supplements (syrup/drops) are used to combat anaemia in children in different parts of the world. However, evidence for functional benefits of iron supplementation in children is scarce, and potential risks remain poorly defined, particularly concerning diarrhoea and malaria. The trial aims to determine if: a) the efficacy of iron supplements or MNPs (containing iron) given with malaria chemoprevention is superior to malaria chemoprevention alone, or b) if the efficacy of malaria chemoprevention alone is superior to placebo on child cognitive development.

Methods and Analysis: IRMA is a four-arm, parallel-group, double-blinded, placebo-controlled, triple-dummy, randomised trial in Southern Malawi. The study recruits 2,168 infants aged six months, with an intervention period of six months and a post-intervention period of a further six months. Children are randomised into four arms: (a) No intervention (placebo); (b) malaria chemoprevention only; (c) MNPs and Malaria chemoprevention; and (d) Iron syrup and malaria chemoprevention. The primary outcome, cognitive development (CogCS), is measured at the end of the six months intervention. Secondary outcomes include CogCS at a further six months post-intervention, and motor, language, behavioural development, physical growth, and prevalence of anaemia and iron deficiency at the end of the six months intervention and a further six months post intervention. Safety outcomes include incidence of malaria and other infections, and prevalence of malaria parasitaemia post-intervention and after six months further follow-up.

Ethics and dissemination: The trial is approved by the National Health Sciences Research Committee (#19/01/2213) (Malawi) and the Human Research Ethics Committee (WEHI:

For peer review only

For peer review only - http://bmjopen.bmj.com/site/about/guidelines.xhtml

19/012) (Australia). Results will be shared with the local community and internationally with academic and policy stakeholders.

Protocol registration details: The protocol is registered with the Australian and New Zealand Clinical Trials Registry (ACTRN12620000386932).

Keywords: Anaemia, Infant, Infection, Iron Deficiency, Malawi, Malaria, Micronutrient Powders, randomised controlled trial.

For peer review only

ARTICLE SUMMARY

Strengths and limitations of this study

- Trial setting: The setting represents malaria-endemic (African) countries where anaemia and malaria co-exist, which is an ideal place to study the benefits versus risks of iron supplementation (under malaria chemoprevention) to establish a generalisable clinical rationale.
- Participant screening/inclusion criteria: Screening involves all infants within the recruitment age range across a large geographical catchment area. This enables generalizability of the study results to most national programs in malaria-endemic countries.
- Intervention: The trial compares the two major delivery modes for iron in young children: MNPs and iron syrup which will help identify the ideal and most effective iron source for children. The trial also includes malaria chemoprevention, which will determine the safety of providing iron supplementation in a population with a high incidence of malaria and other infectious morbidities. Further, the DP-alone versus

For peer review only - http://bmjopen.bmj.com/site/about/guidelines.xhtml

no-intervention arms of the trial will evaluate if malaria chemoprevention alone is effective in improving functional outcomes and anaemia in this context.

- Outcomes: The trial utilises a functional clinical health outcome – child cognitive development – as its primary outcome, ensuring the trial provides evidence of the functional relevance of universal iron interventions and malaria prevention. The trial also measures a broad range of secondary functional, growth, haematologic, biomarker and safety outcomes.
- Sample size and design: The trial is powered to detect a small but clinically relevant effect; the use of a post-intervention follow-up period enables the assessment of both the immediate and medium-term impact of the intervention. Additionally, the trial is double-blind, including a triple dummy, ensuring blinding of participants and all study personnel, including outcome assessors.

For peer review only - http://bmjopen.bmj.com/site/about/guidelines.xhtml

INTRODUCTION

Anaemia remains a critical global health problem affecting up to 39.8% of children aged 6 – 59 months worldwide and 60.2% in Africa.[1-3] In Malawi, 63% of children 6-59 months are anaemic, with a peak prevalence of 91% observed among children aged 9-11 months.[4] Iron deficiency accounts for most (60%) of the global burden of anaemia[5] but less so in malaria-endemic areas, where only 32% of childhood anaemia is iron responsive.[1]

World Health Organisation (WHO) anaemia prevention guidelines recommend two universal iron supplementation strategies for children, namely; 1) iron supplement drops or syrups (10-12.5mg of elemental iron) – given daily to children 6-24 months in areas where the prevalence of anaemia is 40% or higher;[6-8] and 2) iron-containing multiple micronutrient powders (MNPs): 10-12.5mg of iron – given daily over six months in areas where the prevalence of anaemia is 20% or higher.[6-8] However, no recommendation is given regarding the preferred approach. MNPs are the most common form of iron supplementation, with at least 58 countries reported to have implemented MNPs home fortification by 2021.[9] The rationale for universal iron supplementation is an understanding that iron supplementation would reduce childhood anaemia and improve child development and other functional health outcomes. However, we recently showed that in a rural South Asian population, neither universal distribution of oral iron supplements nor MNPs meaningfully improved immediate or medium-term child cognitive, language, motor, and other developmental outcomes, even though anaemia and iron deficiency were reduced.[10] However, these data were generated from a trial in a non-malaria endemic part of South Asia, where the determinants of anaemia and infection risk are likely very different.

Benefits of universal iron supplementation programmes

For peer review only - http://bmjopen.bmj.com/site/about/guidelines.xhtml

For peer review only

Universal iron supplementation programs have shown benefits in haematological outcomes. A meta-analysis[11] and a Cochrane review[12] showed improvements in anaemia with daily iron supplementation in children. However, the effects on growth and development are less clear. Few studies have investigated the impact on cognitive development. Only 8 of the 33 studies included in the meta-analysis by Pasricha et al. assessed cognitive development, and no significant beneficial effect was seen (mean difference 1.65 [95% CI -0.63, 3.94]), but data were sparse.[11] Subgroup analysis did suggest a benefit of iron supplementation on cognitive development in iron-deficient children. However, this data came from only two studies.[13 14] Only one study in the Cochrane review assessed child development, showing that children receiving MNPs were more likely to walk independently at 12 months of age than those receiving no intervention.[15] There is also little evidence for a beneficial effect on growth;[11 12 16] however, data were limited.[11] WHO has acknowledged the need for further research into the functional outcomes of iron supplementation programs.[7]

Risks of iron supplementation

Whilst the evidence for functional benefits of iron supplementation is scarce, there has been emerging evidence of potential harm, particularly regarding increased malaria risk in endemic areas. The seminal Pemba RCT in Tanzania was stopped early due to increased death, hospitalisation and malaria in the children receiving iron compared with placebo and called for revisions of universal iron supplementation recommendations.[17] In another RCT in Tanzania, multi-nutrient supplementation (including iron) increased the number of malaria cases by 41% in iron-deficient children.[18] However, a 2016 Cochrane review found iron supplementation safe in areas with adequate malaria prevention and management strategies.[19] In contrast, there was an increased risk in areas where this was not the case.[19]

For peer review only - http://bmjopen.bmj.com/site/about/guidelines.xhtml

The current recommendation is for universal iron supplementation in malaria-endemic regions with a high prevalence of anaemia only "in conjunction with public health measures to prevent, diagnose and treat malaria."[7]

Iron deficiency protects against malaria

There is evidence to suggest that iron deficiency is protective against malaria. A study of preschool children in Malawi found that iron-deficient children were at lower risk of clinical malaria and parasitaemia than iron-replete children.[20] *In vitro* studies have also demonstrated that *Plasmodium falciparum* parasites are less effective at invading iron deficient red cells and prefer invading reticulocytes (which are increased in the recovery phase of iron deficiency anaemia when iron supplementation is given).[21] Parasite invasion and growth were reduced in laboratory growth and invasion studies of *P. falciparum* using red cells from anaemic children.[22] The protective effect imparted by iron deficiency in both in vitro studies was reversed when iron supplementation was given.[21 22]

Anaemia in malaria-endemic areas is less iron-responsive

Malaria is a significant cause of severe anaemia, hospitalisation, and death in endemic areas.[23-25] Several aetiologies, including malaria, were identified in a study of Malawian children with severe anaemia. However, iron deficiency was not a common contributor.[23] Similarly, in a study in children from The Gambia and Tanzania,[26] 61.2% of children were anaemic, but only 13% had iron deficiency anaemia.[26] In keeping with this, the anaemia in these areas is less iron-responsive.[11 27] Without clear evidence of the neurodevelopmental benefits of iron supplementation, iron interventions are likely to be extremely expensive public health interventions in terms of cost per DALY averted, especially in sub-Saharan Africa.[28]

For peer review only - http://bmjopen.bmj.com/site/about/guidelines.xhtml

Malaria may be a risk factor for impaired cognitive development

Despite few studies, the majority with relatively small sample sizes and varying methods of assessing cognitive function, evidence suggests that severe and uncomplicated malaria may be independent risk factors for cognitive impairment.[29] In addition, intermittent preventive therapy in preschool and school-aged children, has improved cognitive performance.[30 31]

Intermittent preventive treatment to decrease malaria and its sequelae

Intermittent Preventive Treatment in Children (IPTc) involves the empiric treatment of malaria at regular intervals and is a potential strategy to decrease malaria rates and sequelae. A Cochrane review that included seven RCTs evaluated the effect of chemoprophylaxis and intermittent treatment for malaria in preschool children in areas with seasonal malaria and showed a reduction in clinical malaria and moderate to severe anaemia.[32] Furthermore, in a meta-analysis, iron supplementation reduced the rate of anaemia by 30% in malaria-endemic areas, compared with 60% in non-endemic regions.[11]

DP as chemoprevention

Recommended treatment for *P. falciparum* malaria is with artemisinin-based combination therapy (ACT). DP is an ACT and is among WHO's five recommended first-line treatments.[33] DP with its long half-life, given monthly, is an attractive option for IPTc. In an RCT in children in Uganda using this regimen, DP reduced clinical malaria by 58% (compared with 28% for trimethoprim-sulfamethoxazole and 7% for sulfadoxine-pyrimethamine) and parasitaemia by 76%. IPTc with DP also reduced moderate-to-severe anaemia by 47%.[34] The administration of DP-IPTc during MNPs/iron supplementation may improve the safety of these interventions. However, there are concerns that malaria prevention may delay the development of immunity and increase malaria risk once IPTc is ceased.[32]

For peer review only - http://bmjopen.bmj.com/site/about/guidelines.xhtml

Iron deficiency and malaria co-exist in Malawi

Despite important gains made worldwide in malaria control since 2010, worldwide cases increased in 2016. In Malawi, there were a total of 5.2 million presumed and confirmed malaria cases in 2016, 231,970 more cases than the year prior, resulting from a gradual increase in cases since 2014.[35] In addition, the prevalence of malaria parasitaemia in children under five years increased from 28% in 2012 to 33% in 2014. These increases come despite increases in insecticide-treated bed net coverage.[36]

Both malaria and iron deficiency anaemia remain important causes of morbidity and mortality in Malawi. In Malawian children under five years of age, malaria is the leading cause of death,[37] and the prevalence of anaemia is 63%.[38] Addressing both of these conditions is challenging. Current WHO recommendations for improving anaemia in malaria-endemic regions are difficult to interpret and implement in countries like Malawi, where malaria preventive measures and treatment are present but by no means universal.

Why this trial is needed

Several fundamental questions concerning the benefits and safety of iron-intervention programs in malaria-endemic regions remain unanswered. The IRMA trial will assess functional health outcomes from iron supplementation, identify the ideal mode of iron delivery (MNPs versus iron syrup/drops), and determine whether adding IPT diminishes potential risks. It will also allow evaluation of the effect of malaria chemoprevention alone on anaemia and child development.

METHODS AND ANALYSIS

Trial objectives

For peer review only - http://bmjopen.bmj.com/site/about/guidelines.xhtml

The primary objective is to determine a) if the efficacy of iron supplements or MNPs (containing iron) given with malaria chemoprevention is superior to malaria chemoprevention alone, or b) if the efficacy of malaria chemoprevention alone is superior to placebo on child cognitive development (measured using locally adapted Bayley Scales of Infant and Toddler Development 3rd Edition (Bayley-III) Cognitive Composite Scores) at the end of the 6-month intervention period.

The specific objectives are:

1. Determine if the efficacy of a) iron (as supplements or MNPs) given with malaria chemoprevention is superior to malaria chemoprevention alone or b) if the efficacy of malaria chemoprevention alone is superior to placebo on;
   i. child cognitive development, child motor, language, behavioural development, temperament, and physical growth at the end of the six months intervention and a further six months post-intervention;
   ii. anaemia (and haemoglobin concentration), iron deficiency (and ferritin) and iron deficiency anaemia after six months of intervention and six months post intervention.
2. Compare the effects of iron given as supplements to MNPs on the above outcomes after six months of intervention and a further six-months post-intervention.
3. Determine the safety of iron (as supplements or MNPs) given with malaria chemoprevention on the risk of malaria and other morbidities versus malaria chemoprevention alone or placebo during and post-intervention.

For peer review only

For peer review only - http://bmjopen.bmj.com/site/about/guidelines.xhtml

4. Determine the efficacy of malaria chemoprevention alone compared to placebo on clinical malaria, parasitaemia and malaria-specific clinic visits, and all-cause sick visits during and after intervention.
5. Determine the safety of iron (as supplements or MNPs) given with malaria chemoprevention compared to placebo on infection risk in young children, during and post-intervention.

Study design

IRMA is a four-arm parallel-group, double-blinded, placebo-controlled, triple-dummy, individually randomised trial comparing: (a) No intervention, (b) Malaria chemoprevention only, (c) MNPs + Malaria chemoprevention and (d) Iron syrup + Malaria chemoprevention at the end of the six months intervention and at a further 6-months post-intervention. The trial design is summarised in **Figure 1**.

Study settings and participants

The trial is set in Chikwawa district, in Southern Malawi, a rural area where >70% of children 6-12 months are anaemic,[39] and where 33% of children under five years old have *Plasmodium falciparum* parasitaemia.[40] Four health facilities within the district are hosting the trial, namely: Chikwawa District Hospital, Mapelera Health Centre, Mfera Health Centre and Makhwira Health Centre. These are the primary health facilities where children attend routine under-five clinics and sick visits. Our target participants are all children aged six months residing in these health facilities' catchment area.

Eligibility criteria

For peer review only

For peer review only - http://bmjopen.bmj.com/site/about/guidelines.xhtml

The criteria to select participants considers participants' safety, the generalizability to national programs, and the feasibility of the trial. Children are randomly assigned to a study intervention group only if they meet all the inclusion and none of the exclusion criteria.

Inclusion criteria

1. Infant aged six months (+/- 14 days) at randomisation.
2. Availability of a legally acceptable guardian/representative and providing written consent on the participant's behalf.

Exclusion criteria

1. Severe anaemia (Hb<7g/dL).
2. Clinical signs of current infective illness (respiratory infection, diarrhoea, malaria); however, children will be screened after recovery and recruited if they meet the age eligibility criteria at the second screening.
3. Severe malnutrition (weight-for-length z-score<-3) or MUAC < 11.5 cm.
4. Established haemoglobinopathy diagnosis (e.g., sickle cell disease, beta thalassaemia major, HbE-beta thalassaemia).
5. Known congenital anomaly, developmental disorder, or severe developmental delay.
6. Children with physical or behavioural problems that will make it impossible to undertake trial assessments.
7. Children of multiple births, e.g., twins.
8. Children currently consuming iron supplements or MNPs, e.g., from the National MNPs programme.
9. Enrolment in another trial.

For peer review only

For peer review only - http://bmjopen.bmj.com/site/about/guidelines.xhtml

Trial Interventions

Participants are randomly assigned to receive one of the following interventions:

1) **No intervention (Placebo DP *plus* Placebo MNPs *plus* placebo iron syrup)**: Placebo DP (Guilin Pharmaceutical company, China)**:** three consecutive days every four weeks for six months *plus* Placebo MNPs (maltodextrin vehicle only; DSM Nutritional Products, South Africa) daily for six months *plus* placebo iron syrup (ACME Laboratories Ltd., Bangladesh) daily for six months.
2) **Malaria chemoprevention only (Active DP *plus* placebo MNPs *plus* placebo iron syrup)**: Active DP (20mg dihydroartemisinin/160mg piperaquine; Guilin Pharmaceutical company, China) dosed by body weight for three consecutive days every four weeks for six months *plus* placebo MNPs daily for six months, *plus* daily placebo iron syrup for six months.
3) **MNP + malaria chemopevention (Active DP *plus* active MNPs *plus* placebo iron syrup):** Active DP as above, *plus* daily active MNPs containing 10.0mg iron and 14 other micronutrients (see supplementary material 1); DSM Nutritional Products, South Africa, *plus* placebo iron syrup daily for six months.
4) **Iron syrup + malaria chemoprevention (Active DP *plus* placebo MNPs *plus* active iron syrup):** Active DP as above, *plus* placebo MNPs, *plus* active iron syrup containing 10.0mg ferrous sulphate (ACME Laboratories Ltd., Bangladesh) daily for six months.

Randomisation, allocation concealment, and blinding

Participants are randomly allocated to one of the four study arms with 1:1:1:1 allocation via a computer-generated randomisation schedule of randomly permuted blocks stratified by sex and health facility/study sites (Chikwawa District Hospital, Mapelera health centre, Mfera

For peer review only

For peer review only - http://bmjopen.bmj.com/site/about/guidelines.xhtml

health centre and Makhwira Health Centre) to achieve a balance between the intervention arms. An independent statistician from the University of Melbourne (Australia) generated the randomisation list.

An independent Trial Pharmacist pre-packed and pre-labelled participant packs containing all three investigational products based on the randomisation list. Each participating child who meets the inclusion criteria is sequentially allocated to the next available study number indicated on the randomisation list. The participant is then given a pre-labelled pre-packed Ziplock bag containing the study medication corresponding to the study number assigned to them. Hence, participants and their families and all study team members involved in collecting and analysing outcome data, including field workers, developmental assessors, laboratory scientists, statisticians, and investigators, are blinded to the treatment allocation until the database is locked and before unblinding.

Recruitment and Visits

Patient and Public Involvement

All levels of the local leadership (head men/women, sub-chiefs, chiefs, Community Health Action Groups (CHAGS), Health Surveillance Assistants (HSAs) and District Health Assembly (DHA)) are continuously engaged by the trial’s Community Engagement Team (CET). CHAGs and HSAs were engaged to ensure that we reach the whole community via it's resident health “gate-keepers”. Additionally, the CET conducted community sensitisation meetings to explain the research objectives, what it means to participate, benefits for the individual or the community, and overall contribution to science. Finally, community members are regularly invited to contribute ideas that could help improve the success of the research thereby increasing the probability of success.

For peer review only

For peer review only - http://bmjopen.bmj.com/site/about/guidelines.xhtml

315

316 ***Screening and enrolment of participants***

317 Study recruitment opened on 17 April 2020. Study activities are as detailed in **Table 1.**

For peer review only

For peer review only - http://bmjopen.bmj.com/site/about/guidelines.xhtml

Table 1. Main study activities per visit

		Intervention period (Active follow-up)													Post intervention period (Passive follow up)					
	**Month**	**M1**			**M2**		**M3**		**M4**		**M5**		**M6**		**M7**	**M8**	**M9**	**M10**	**M11**	**M12‡**
**Planned activity**	**Day** 0	D 1#	D 14	D 28	D 42	D 56	D 70	D 84	D 98	D 112	D 126	D 140	D 154	D 168§						
**Location**	**H/HF**	**HF**	**H**	**H**	**H**	**H**	**H**	**H**	**H**	**H**	**H**	**H**	**H**	**HF**	**H**	**H**	**H**	**H**	**H**	**HF**
Pre-screening/ screening, & informed consent	**X**	**X**																		
Randomization		**X**																		
Demographic, clinical, and SES assessments		**X**																		
Family Care Index (FCI)		**X**												**X**						**X**
Bayley-III Cognitive Composite Score (CogCS)														**X**						**X**
Bayley-III Motor (MotCS) and Language (LangCS) Composite Scores														**X**						**X**
Behaviour: Wolke's Behaviour Ratings (WBR)														**X**						**X**
Food security & dietary diversity questionnaires		**X**												**X**						**X**
Anthropometry[1] - (mother and child)		**X**												**X**						**X**
**Laboratory procedures**																				
Haemoglobin		**X**												**X**						**X**

For peer review only - http://bmjopen.bmj.com/site/about/guidelines.xhtml

Malaria microscopy		X												X						X
Serum iron marker tests[2]		X												X						X
Serum for inflammatory marker tests (CRP/AGP)[3]		X												X						X
**Active follow-up:**																				
Compliance assessment		X	X	X	X	X	X	X	X	X	X	X	X	X						
MNPs/Placebo MNPs[4](distribute, instruct)		X		X		X		X		X		X								
DP/Placebo DP[5] (distribute, instruct & observe use/intake)		X		X		X		X		X		X								
Iron syrup/Placebo iron syrup[6]		X		X		X		X		X		X								
Provide educational information		X	X	X	X	X	X	X	X	X	X	X	X	X						
Assess infections[7]			X	X	X	X	X	X	X	X	X	X	X	X	X	X	X	X	X	X
Adverse Events monitoring			X	X	X	X	X	X	X	X	X	X	X	X	X	X	X	X	X	X
Health care utilization monitoring		X	X	X	X	X	X	X	X	X	X	X	X	X	X	X	X	X	X	X

# - Baseline; § - Midline visit at day 168. Primary outcome was assessed at day 168 ± 14 days as per protocol)**; ‡ - Endline; M –** Month; **D –** Day; **H – Home** (visit at the Participant's Home)**; HF –** Visit at the Health Facility; **X** – scheduled activity. **SES -** Social Economic Status; [1]**Anthropometric assessments** (mother and child): height/length, weight; [2]**Serum iron marker tests**: Serum ferritin; [3]**Inflammation markers**: CRP (C-Reactive Protein) or AGP (alpha 1 acid glycoprotein); [4]**MNPs** containing 10.0mg iron and 14 other micronutrients, daily for 6 months; **Placebo MNPs**: (maltodextrin), daily for 6 months; [5]**DP/Placebo DP** (20mg dihydroartemisinin/ 160mg piperaquine in suspension) dosed by body weight, three consecutive days every 4 weeks for 6 months; [6]**Iron syrup/Placebo iron syrup:** 10mg daily for six months; [7]**Infections**(assessment): (rate and number of days affected) fever/respiratory/diarrhoea.

NB: Families were encouraged to permit collection of venous blood for venous haemoglobin and iron and inflammation biomarkers at all timepoints; however, if a family refused venous blood collection or if collection was unsuccessful, data for the primary outcome and other non-laboratory secondary outcomes was still collected.

For peer review only

For peer review only - http://bmjopen.bmj.com/site/about/guidelines.xhtml

Pre-screening

We conducted a house-to-house enumeration (census) of all potential participants (children under six months of age) living in the target area and generated a study population database. New births are identified and reported by HSAs or registered at the health facilities' under-five clinics throughout the trial recruitment period and added to the database. Research Assistants visit all children approaching six months of age in the community. After taking written informed consent, the team reviews the child's medical history to determine potential eligibility. Eligible children are invited to visit a selected health facility for further screening.

Screening

On a pre-determined day, the child, accompanied by their parent(s) or legal guardian, visit the selected health centre. HemoCue Hb301+ is used to assess capillary Hb level to exclude severely anaemic children. Additionally, anthropometric measurements: weight and length (used to calculate weight-for-length z-scores[41]) and mid-upper arm circumference (MUAC) are performed to exclude severely malnourished children. Severely anaemic and severely malnourished children are referred to the routine health system for further investigation and treatment.

Baseline and Enrolment (at six months of age (+/- 14 days))

A second, more detailed consent form for participation in the study is signed, and a study identification number is assigned. Randomisation is performed after the following baseline evaluations (see **Table 1**) are carried out and necessary information collected: demographic information, socioeconomic status (using the Malawi Demographic Health Survey (MDHS) standard of living scale), medical history, family care indicators/index (FCI), feelings questionnaire, cost survey (household out of pocket health care costs), food security

For peer review only - http://bmjopen.bmj.com/site/about/guidelines.xhtml

questionnaire, dietary diversity questionnaire, developmental screening using Caregiver Reported Early Development Instrument (CREDI), anthropometric measurements (weight, length, MUAC and head circumference), and anthropometric measurements for the mother (weight, height). A venous blood sample (3 mL), for haemoglobin testing, determination of iron indices (serum – for ferritin, CRP, and AGP), thick and thin malaria slides for malaria microscopy testing (to be conducted at the completion of the trial) and filter paper for plasmodium PCR, is collected from the child. The mother is given educational information regarding the use of study medications, administration of the first dose of DP and instructions on how to administer day two and day three DP treatments, administration of the first dose of iron syrup, use of MNPs and advice on future visits.

Subsequent study visits

Adherence assessment

Fortnightly 'home visits' by experienced research assistants to the participants' homes in the community are done to assess the following: 1) documentation of episodes of infection (rate, days affected), 2) recording any adverse events/effects, 3) documentation of details of unplanned hospital and healthcare facility attendance for example to a doctor, traditional healer, clinic or hospital, 4) provision of educational information to participants on the correct use of the study interventions and encouraging adherence to study interventions, 5) adherence monitoring (measured from parental questioning along with assessment of residual MNPs and syrup) and 6) advice on future visits.

Monthly DP issuance

The research assistants issue the investigational products monthly during a home visit. The following activities (including data collection) are undertaken: issuance of the study

For peer review only

For peer review only - http://bmjopen.bmj.com/site/about/guidelines.xhtml

interventions according to study protocol and adherence monitoring (measured from parental questioning along with assessment of residual MNPs and syrup). In addition, unused investigational products are collected.

Study midline visit (at 12 months of age +/- 14 days)

The participating child and their parent/ legal guardian attend the health facility at the end of the six months intervention for developmental assessments using locally adapted Bayley Scales of Infant and Toddler Development, Wolke's Behavioural Rating (WBR), and Child Temperament using the Bates' infant characteristics questionnaire. Anthropometric measurements are conducted on both the child and the mother. Venous blood (3 mL) for haemoglobin testing, determination of iron indices, malaria microscopy (to be conducted at the completion of the trial) and malaria filter paper for future PCR analyses is collected from the child.

Monthly malaria infection testing

Research assistants visit the homes of participants once per month after completion of the midline visit for assessment of episodes of infection (rate, number of days affected) in the previous one month and documentation of unplanned hospital and/or healthcare facility attendance (details of any interim visit to doctor, clinic or hospital visits in the previous month) and record of healthcare utilisation.

Endline visit (at 18 months of age (-2 weeks or +6 weeks)

The procedures are similar to the midline visit described above and in **Table 1**.

Unscheduled visits

For peer review only

For peer review only - http://bmjopen.bmj.com/site/about/guidelines.xhtml

Mothers or legal guardians are asked to attend the research site/clinic when their child is symptomatically unwell. They are managed according to national standard treatment guidelines by a trained healthcare provider. Blood samples for malaria rapid diagnostic test (RDT) and blood culture are taken if clinically indicated. The clinical diagnosis of the unscheduled visit is recorded in the participant's record and captured on the study's interim visit Case Report Form (CRF) and adverse event form. If the child requires inpatient care, they are referred to the Chikwawa district hospital.

Laboratory Procedures

A capillary blood sample is collected by finger prick from all age-eligible participants to measure the haemoglobin concentration by HemoCue (HemoCue 301+, Angelholm, Sweden) and *Plasmodium* infection by rapid diagnostic test (mRDTs); (SD Bioline Malaria Ag Pf/Pan®, Standard Diagnostics) at baseline. In addition, up to 3 mL venous blood sample are collected from all enrolled participants at baseline, midline, endline and unscheduled visits (if necessary)) for haemoglobin testing, determination of iron indices (serum – for ferritin, CRP, and AGP), thick and thin malaria slides for malaria microscopy testing and filter paper for *Plasmodium* PCR. Families are encouraged to permit the collection of venous blood for venous haemoglobin and iron and inflammation biomarkers at all time points; however, if a family refuses venous blood collection or if collection was unsuccessful, data for the primary outcome and other non-laboratory secondary outcomes will still be collected.

Data Safety and Monitoring Committee (DSMC)

An independent DSMC comprised of international experts in clinical trials, child health, infectious diseases, epidemiology, and statistics was set up to review regularly, safety data of

For peer review only

For peer review only - http://bmjopen.bmj.com/site/about/guidelines.xhtml

the ongoing trial. The DSMC recommend to the sponsor and investigators whether to continue, modify, or terminate the trial on ethical grounds.

Outcomes

The primary outcome

Cognitive development: assessed by the differences in Bayley-III Cognitive Composite Score (CogCS) between intervention arms measured after six months intervention (midline) and 12 months post-intervention (endline). The Bayley-III is a validated index of child development that can be administered in ~0.5-1 hour and comprises cognitive, motor, and language development scales. Assessors are specially trained to administer the tests, and inter-rater reliability is established before starting data collection. In addition, we perform 10% inter-observer testing to monitor inter-rater reliability of the measure over the study period.

Secondary outcomes

Secondary outcomes include: motor and language development assessed by Bayley-III Motor (MotCS) and Language (LangCS) Composite Scores (+6m, +12m), behaviour assessed by WBR, temperament, growth assessed by weight-for-age z-score, length-for-age z-score, weight-for-length z-score, head circumference z-score, anaemia (Hb<11g/dL), haemoglobin concentration, Iron Deficiency (Ferritin<12ng/uL adjusted for inflammation assessed by CRP or AGP), ferritin concentration, and Iron Deficiency Anaemia, as well as safety assessed by parasitaemia, adverse events, clinical malaria, C-reactive protein (CRP) and inflammation (CRP>5mg/L), incidence of unplanned hospital/ health-care facility attendance, diarrhoea, fever, respiratory symptoms, constipation, vomiting, and death.

Detection and reporting of Adverse Events and Serious Adverse Events

For peer review only - http://bmjopen.bmj.com/site/about/guidelines.xhtml

Non-serious Adverse Events (AEs) and serious adverse events (SAEs) are collected from the time consent is given until the participant completes the study (the final visit or withdrawal). These are identified through spontaneous reports by the participant, unplanned visits to the research site or any participating health centres, observation by the study staff, and standard questioning at each visit (biweekly during the intervention period and monthly during the postintervention period. All SAEs are reported to the sponsor and the appropriate ethics committee, whether or not they are considered causally related to the investigational products.

Sample size

The primary objective will be met if either i) MNPs+DP is superior to DP alone, or ii) Iron+DP alone is superior to DP alone or iii) DP alone is superior to placebo on the Bayley-III CogCS immediately after completion of the intervention (midline). A previous study in 2853 Bangladeshi children showed that a 0.2 standardised mean difference (SMD) in Bayley-II Mental Development Index at seven months translates to a 0.7 SMD at 18 months and a 1.2 SMD at 64 months age.[42] Therefore, preventing a loss of at least 0.2 SMD may produce magnified long-term developmental benefits as manifestations of brain dysfunction become more evident. A total sample size of 2168 infants (542 per group) will provide 90% power to detect an effect size of 0.25 SMD, assuming a 20% dropout and a two-sided alpha of 1.67% (Bonferroni adjustment for three primary between-group comparisons).

Statistical Analysis Plan

A stand-alone statistical analysis plan will be detailed before breaking the blind. Participants will be analysed according to their randomised treatment group for efficacy objectives and actual treatment group for safety objectives. The primary outcome of Bayley-III CogCS will be

For peer review only - http://bmjopen.bmj.com/site/about/guidelines.xhtml

analysed using a mixed-model repeated measures (MMRM) analysis including in the model treatment group, time-point, and group by time-point interaction. The stratification variables sex and site will be included in all models as main effects. Secondary repeated continuous outcomes (e.g., growth outcomes) will be analysed similarly to Bayley-III CogCS. The outcome may be appropriately transformed before fitting the analysis model if considered skewed (e.g., log-transformation for ferritin). Secondary repeated binary outcomes (e.g., haematologic outcomes) will be analysed using a log-binomial model and account for the repeated measures with a random intercept for participant. Safety (e.g., AEs, infections, hospital attendance) will be summarised during the intervention and post-intervention periods separately and compared between the groups using log-binomial regression. To investigate the robustness of the results to non-negligible missing data[43] additional analysis of multiply imputed missing outcome data will be conducted. Subgroup analyses (e.g., sex, site, iron deficiency) will be performed to explore the heterogeneity of the results independent of the study findings.

Data Management

Data collected from the subjects are recorded in digital Case Report Forms (CRFs) using Redcap software on tablets. Relevant hard copy patient hospital files are scanned for reference and stored digitally, and securely. The trial is independently monitored by an external monitoring service. No interim analysis is planned.

ETHICS AND DISSEMINATION

The National Health Science Research Committee, Malawi, and the Walter and Eliza Hall Institute of Medical Research Human Research Ethics Committee, Australia, approved this study. The Malawian Pharmacy and Medicines Regulatory Authority (PMRA) also approved

For peer review only - http://bmjopen.bmj.com/site/about/guidelines.xhtml

the trial. Additionally, the protocol is registered with the Australian and New Zealand Clinical Trials Registry (ACTRN12620000386932). Protocol modifications are reported to the ethics committees, regulatory authorities in Malawi, investigators, trial participants and trial registries. Written informed consent in the local language is obtained from each participant before conducting any study-related procedure. The results will be presented and shared with the local community that hosted the research and to the international forum. We will publish in peer-reviewed scientific journals and report to relevant policymaking bodies like the Malawi Ministry of Health.

Discussion

More than two-thirds of African children aged 6-59 months are anaemic. The IRMA trial will provide valuable evidence on the immediate and medium-term impacts of iron interventions (both supplements and MNPs) and malaria prevention with DP on critical functional outcomes, most importantly, child cognitive development. These findings will complement high-quality data recently reported in South Asia to provide generalisable evidence for or against universal iron interventions across sub-Saharan Africa and other malaria-endemic settings.

For peer review only - http://bmjopen.bmj.com/site/about/guidelines.xhtml

References

1. World Health Organization. The global prevalence of anaemia in 2011. Geneva, Switzerland: WHO, 2015.

2. Stevens GA, Paciorek CJ, Flores-Urrutia MC, et al. National, regional, and global estimates of anaemia by severity in women and children for 2000-19: a pooled analysis of population-representative data. *Lancet Glob Health* 2022;10(5):e627-e39. doi: 10.1016/s2214-109x(22)00084-5 [published Online First: 2022/04/16]

3. World Health Organization. WHO Global anaemia estimates, 2021 Edition. Global anaemia estimates in women of reproductive age, by pregnancy status, and in children aged 6-59 months. Geneva, Switzerland: WHO; 2021 [cited 2022 28 August]. Available from: https://www.who.int/data/gho/data/themes/topics/anaemia_in_women_and_children accessed 28 August 2022.

4. National Statistical Office (NSO) [Malawi] and ICF. Malawi Demographic and Health Survey 2015-16,. Zomba, Malawi and Rockville, Maryland, USA: NSO and ICF, 2017.

5. Institute for Health Metrics and Evaluation (IHME). Global Burden of Disease (GBD) 2022 [cited 2022. Available from: https://www.healthdata.org/gbd/2019 accessed 28 August 2022.

6. World Health Organization. Use of multiple micronutrient powders for point-of-use fortification of foods consumed by infants and young children aged 6-23 months and children aged 2-12 years. Geneva, Switzerland: WHO, 2016a.

7. World Health Organization. Guideline: Daily iron supplementation in infants and children. Geneva, Switzerland: WHO, 2016b.

8. World Health Organization, United Nations Children's Fund, United Nations University. Iron Deficiency Anaemia: Assessment, Prevention, and Control. A guide for programme managers. Geneva, Switzerland: WHO, 2001.

9. UNICEF. Multiple Micronutrient Powder: Supply and market update - July 2021. New York: UNICEF, 2021.

10. Pasricha SR, Hasan MI, Braat S, et al. Benefits and Risks of Iron Interventions in Infants in Rural Bangladesh. *N Engl J Med* 2021;385(11):982-95. doi: 10.1056/NEJMoa2034187 [published Online First: 2021/09/09]

11. Pasricha SR, Hayes E, Kalumba K, et al. Effect of daily iron supplementation on health in children aged 4-23 months: a systematic review and meta-analysis of randomised controlled trials. *Lancet Glob Health* 2013;1(2):e77-e86. doi: 10.1016/s2214-109x(13)70046-9 [published Online First: 2014/08/12]

12. De-Regil LM, Suchdev PS, Vist GE, et al. Home fortification of foods with multiple micronutrient powders for health and nutrition in children under two years of age. *Cochrane Database Syst Rev* 2011(9):Cd008959. doi: 10.1002/14651858.CD008959.pub2 [published Online First: 2011/09/09]

13. Akman M, Cebeci D, Okur V, et al. The effects of iron deficiency on infants' developmental test performance. *Acta Paediatr* 2004;93(10):1391-6. [published Online First: 2004/10/27]

14. Idjradinata P, Pollitt E. Reversal of developmental delays in iron-deficient anaemic infants treated with iron. *Lancet* 1993;341(8836):1-4. doi: 10.1016/0140-6736(93)92477-b [published Online First: 1993/01/02]

For peer review only - http://bmjopen.bmj.com/site/about/guidelines.xhtml

15. Adu-Afarwuah S, Lartey A, Brown KH, et al. Home fortification of complementary foods with micronutrient supplements is well accepted and has positive effects on infant iron status in Ghana. *Am J Clin Nutr* 2008;87(4):929-38. doi: 10.1093/ajcn/87.4.929 [published Online First: 2008/04/11]
16. Somassè YE, Dramaix M, Traoré B, et al. The WHO recommendation of home fortification of foods with multiple-micronutrient powders in children under 2 years of age and its effectiveness on anaemia and weight: a pragmatic cluster-randomized controlled trial. *Public Health Nutr* 2018;21(7):1350-58. doi: 10.1017/s1368980017003858 [published Online First: 2018/01/22]
17. Sazawal S, Black RE, Ramsan M, et al. Effects of routine prophylactic supplementation with iron and folic acid on admission to hospital and mortality in preschool children in a high malaria transmission setting: community-based, randomised, placebo-controlled trial. *Lancet* 2006;367(9505):133-43. doi: 10.1016/s0140-6736(06)67962-2 [published Online First: 2006/01/18]
18. Veenemans J, Milligan P, Prentice AM, et al. Effect of supplementation with zinc and other micronutrients on malaria in Tanzanian children: a randomised trial. *PLoS Med* 2011;8(11):e1001125. doi: 10.1371/journal.pmed.1001125 [published Online First: 2011/12/02]
19. Neuberger A, Okebe J, Yahav D, et al. Oral iron supplements for children in malaria-endemic areas. *Cochrane Database Syst Rev* 2016;2(2):Cd006589. doi: 10.1002/14651858.CD006589.pub4 [published Online First: 2016/02/28]
20. Jonker FA, Calis JC, van Hensbroek MB, et al. Iron status predicts malaria risk in Malawian preschool children. *PLoS One* 2012;7(8):e42670. doi: 10.1371/journal.pone.0042670 [published Online First: 2012/08/24]
21. Clark MA, Goheen MM, Fulford A, et al. Host iron status and iron supplementation mediate susceptibility to erythrocytic stage Plasmodium falciparum. *Nat Commun* 2014;5:4446. doi: 10.1038/ncomms5446 [published Online First: 2014/07/26]
22. Goheen MM, Wegmüller R, Bah A, et al. Anemia Offers Stronger Protection Than Sickle Cell Trait Against the Erythrocytic Stage of Falciparum Malaria and This Protection Is Reversed by Iron Supplementation. *EBioMedicine* 2016;14:123-30. doi: 10.1016/j.ebiom.2016.11.011 [published Online First: 2016/11/18]
23. Calis JC, Phiri KS, Faragher EB, et al. Severe anemia in Malawian children. *N Engl J Med* 2008;358(9):888-99. doi: 10.1056/NEJMoa072727 [published Online First: 2008/02/29]
24. Newton CR, Warn PA, Winstanley PA, et al. Severe anaemia in children living in a malaria endemic area of Kenya. *Trop Med Int Health* 1997;2(2):165-78. doi: 10.1046/j.1365-3156.1997.d01-238.x [published Online First: 1997/02/01]
25. Njua-Yafi C, Achidi EA, Anchang-Kimbi JK, et al. Malaria, helminths, co-infection and anaemia in a cohort of children from Mutengene, south western Cameroon. *Malar J* 2016;15:69. doi: 10.1186/s12936-016-1111-2 [published Online First: 2016/02/08]
26. Pasricha SR, Atkinson SH, Armitage AE, et al. Expression of the iron hormone hepcidin distinguishes different types of anemia in African children. *Sci Transl Med* 2014;6(235):235re3. doi: 10.1126/scitranslmed.3008249 [published Online First: 2014/05/09]
27. Zlotkin S, Newton S, Aimone AM, et al. Effect of iron fortification on malaria incidence in infants and young children in Ghana: a randomized trial. *Jama* 2013;310(9):938-47. doi: 10.1001/jama.2013.277129 [published Online First: 2013/09/05]

For peer review only - http://bmjopen.bmj.com/site/about/guidelines.xhtml

28. Pasricha SR, Gheorghe A, Sakr-Ashour F, et al. Net benefit and cost-effectiveness of universal iron-containing multiple micronutrient powders for young children in 78 countries: a microsimulation study. *Lancet Glob Health* 2020;8(8):e1071-e80. doi: 10.1016/s2214-109x(20)30240-0 [published Online First: 2020/07/28]
29. Fernando SD, Rodrigo C, Rajapakse S. The 'hidden' burden of malaria: cognitive impairment following infection. *Malar J* 2010;9:366. doi: 10.1186/1475-2875-9-366 [published Online First: 2010/12/22]
30. Clarke SE, Jukes MC, Njagi JK, et al. Effect of intermittent preventive treatment of malaria on health and education in schoolchildren: a cluster-randomised, double-blind, placebo-controlled trial. *Lancet (London, England)* 2008;372(9633):127-38. doi: 10.1016/s0140-6736(08)61034-x [published Online First: 2008/07/16]
31. Jukes MCH, Pinder M, Grigorenko EL, et al. Long-Term Impact of Malaria Chemoprophylaxis on Cognitive Abilities and Educational Attainment: Follow-Up of a Controlled Trial. *PLOS Clinical Trials* 2006;1(4):e19. doi: 10.1371/journal.pctr.0010019
32. Meremikwu MM, Donegan S, Sinclair D, et al. Intermittent preventive treatment for malaria in children living in areas with seasonal transmission. *The Cochrane database of systematic reviews* 2012(2):Cd003756. doi: 10.1002/14651858.CD003756.pub4 [published Online First: 2012/02/18]
33. WHO. Guidelines for the treatment of Malaria Third edition ed: World Health Organisation 2015.
34. Bigira V, Kapisi J, Clark TD, et al. Protective Efficacy and Safety of Three Antimalarial Regimens for the Prevention of Malaria in Young Ugandan Children: A Randomized Controlled Trial. *PLoS medicine* 2014;11(8):e1001689. doi: 10.1371/journal.pmed.1001689
35. World malaria report 2017: World Health Organisation, 2017.
36. Zgambo M, Mbakaya BC, Kalembo FW. Prevalence and factors associated with malaria parasitaemia in children under the age of five years in Malawi: A comparison study of the 2012 and 2014 Malaria Indicator Surveys (MISs). *PloS one* 2017;12(4):e0175537. doi: 10.1371/journal.pone.0175537 [published Online First: 2017/04/12]
37. WHO. Malawi: WHO statistical profile: World Health Organisation 2015.
38. Whitson D. Farta Child Survival Project: Final Evaluation: CARE Ethiopia, 2007.
39. National Statistical Office (NSO) and ICF Macro. Malawi Demographic and Health Survey 2010. Zomba, Malawi, and Calverton, Maryland, USA: National Statistical Office (NSO) and ICF Macro 2011.
40. National Malaria Control Programme (NMCP) [Malawi] and ICF International. Malawi Malaria Indicator Survey (MIS) 2014. Lilongwe, Malawi, and Rockville, Maryland, USA: NMCP and ICF International 2014.
41. WHO Multicentre Growth Reference Study Group. Assessment of differences in linear growth among populations in the WHO Multicentre Growth Reference Study. *Acta Paediatr Suppl* 2006;450:56-65. doi: 10.1111/j.1651-2227.2006.tb02376.x [published Online First: 2006/07/05]
42. Hamadani JD, Tofail F, Huda SN, et al. Cognitive deficit and poverty in the first 5 years of childhood in Bangladesh. *Pediatrics* 2014;134(4):e1001-8. doi: 10.1542/peds.2014-0694 [published Online First: 2014/10/01]

For peer review only - http://bmjopen.bmj.com/site/about/guidelines.xhtml

43. Jakobsen JC, Gluud C, Wetterslev J, et al. When and how should multiple imputation be used for handling missing data in randomised clinical trials - a practical guide with flowcharts. *BMC Med Res Methodol* 2017;17(1):162. doi: 10.1186/s12874-017-0442-1 [published Online First: 2017/12/07]

Author Contributions:

MNM drafted the initial study protocol. MNM and GM supervised the trial's implementation and wrote the protocol paper's first draft. CCN and MDK coordinated the implementation of the study. MV and LML planned for the measurement of the primary outcome. SB, RH, and ARDM wrote the statistical analysis plan. MNM and RA provided daily scientific input during the implementation of the trial. B-AB, KSP, and S-RP conceived the trial, sought funding, and provided senior scientific oversight in the conduct of the research. All authors read and approved the final manuscript.

Funding statement: This work is supported by the National Health and Medical Research Council (NHMRC), Australia, grant numbers GNT1141185 and GNT1159151. SP is funded by NHMRC GNT1158696 and GNT2009047.

Competing interests statement.

None to declare.

Acknowledgements:

We thank all the participants of the IRMA trial - including parents or legal guardians - especially during the challenging Covid-19 period. We thank the ethics and regulatory committees mentioned in the manuscript for their extensive review and approval of the study protocol and operational documents. We acknowledge the support from the Malawi government, the Chikwawa District Health Office, including the health management teams of

For peer review only - http://bmjopen.bmj.com/site/about/guidelines.xhtml

Chikwawa District Hospital, Makhwira, Mapelera, and Mfera Health Centres. We thank the Shire River East-and-West bank communities for hosting the study and continuously bearing with the extensive study procedures, especially the conduct of the two censuses to determine the extent of the source population. Finally, the study would not be possible without the support of the over 100 staff members (in Malawi and Australia) who contribute their time and expertise to ensure its success. We say thank you to all.

Data Statement

All relevant data are within the paper and its supporting information files.

Protocol version

IRMA Trial protocol version 4.0 dated July 25, 2022

Ethics and regulatory approvals

Ethical approval was granted by the National Health Sciences Research Committee (#19/01/2213) in Malawi and the Human Research Ethics Committee (WEHI: 19/012) in Melbourne, Australia. In addition, the Malawi Pharmacy and Medicines Regulatory Authority (PMRA) reviewed and approved the study and the purchase and importation of the investigational products to Malawi (clinical trial certificate number PMPB/CTRC/IV/06062019111).

Trial Sponsor

Training and Research Unit of Excellence (TRUE), 1 Kufa Road, Mandala. P.O. Box 30538, Chichiri, Blantyre 3, Malawi. Email: true@true.mw

Roles and responsibilities of sponsor and funder:

For peer review only

For peer review only - http://bmjopen.bmj.com/site/about/guidelines.xhtml

674 The sponsor and funder have no role in the study design. They will have no part or ultimate authority in the collection, management, analysis, and interpretation of data, report writing, and the decision to submit the report for publication.

677 **Figure heading, caption and legend**

678 ***Figure 1***

679 ***Figure caption and legend:*** *IRMA Trial schema. Overall display of IRMA activities. MNP - Iron-containing multiple micronutrient powders; DP - Dihydroartemisinin-piperaquine; Intervention – issued monthly, from enrolment at age six months to 12 months of age. Primary endpoint – Bayley's assessment conducted at the trial midline; Cognitive assessments – Bayley Scales of Infant and Toddler Development 3rd Edition.*

For peer review only

For peer review only - http://bmjopen.bmj.com/site/about/guidelines.xhtml

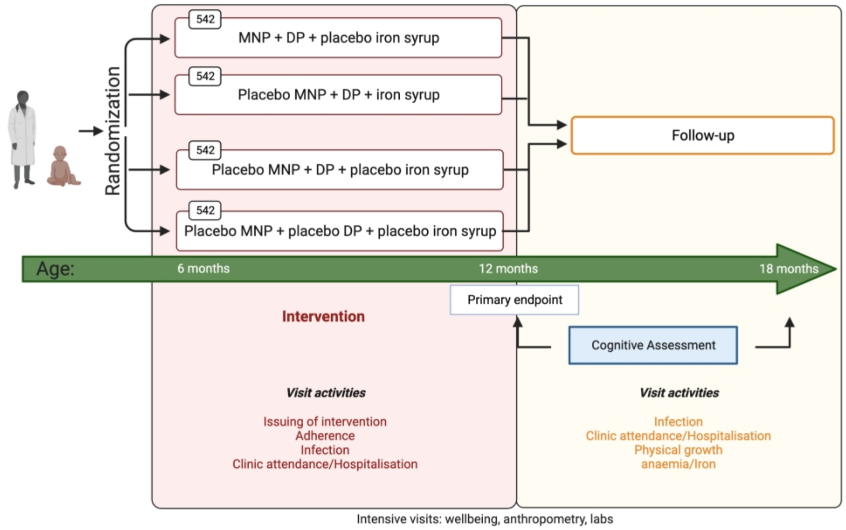

Figure 1: IRMA study design

645x516mm (118 x 118 DPI)

Supplementary material 1: Multiple micronutrient powders (MNPs) contents

	Micronutrient name	Quantity
1.	Niacinamide	7.02 - 8.58 mg/g
2.	Riboflavin	0.653 - 0.798 mg/g
3.	Pyridoxine	0.585 – 0.715 mg/g
4.	Thiamine	0.675 – 0.825 mg/g
5.	Vitamin C	32.4 – 39.6 mg/g
6.	Vitamin A	522 – 667 µgRE/g
7.	Vitamin E (as Tocopherol Eq.)	5.04 – 6.16 mgTE/g
8.	Vitamin D3	6.75 – 8.63 µgD/g
9.	Vitamin B12	1.09 – 1.40 µg/g
10.	Folic Acid	109 – 134 µg/g
11.	Selenium	17.0 – 24.5 µg/g
12.	Iodine	9.00 -129.6 µg/g
13.	Iron	10.00 -12.10 mg/g
14.	Zinc	4.10 – 0.739 mg/g
15.	Copper	0.605 – 0.739 mg/g

For peer review only

Reporting checklist for protocol of a clinical trial.

Based on the SPIRIT guidelines.

Instructions to authors

Complete this checklist by entering the page numbers from your manuscript where readers will find each of the items listed below.

Your article may not currently address all the items on the checklist. Please modify your text to include the missing information. If you are certain that an item does not apply, please write "n/a" and provide a short explanation.

Upload your completed checklist as an extra file when you submit to a journal.

In your methods section, say that you used the SPIRITreporting guidelines, and cite them as:

Chan A-W, Tetzlaff JM, Gøtzsche PC, Altman DG, Mann H, Berlin J, Dickersin K, Hróbjartsson A, Schulz KF, Parulekar WR, Krleža-Jerić K, Laupacis A, Moher D. SPIRIT 2013 Explanation and Elaboration: Guidance for protocols of clinical trials. BMJ. 2013;346:e7586

		Reporting Item	Page Number
**Administrative information**			
Title	#1	Descriptive title identifying the study design, population, interventions, and, if applicable, trial acronym	1
Trial registration	#2a	Trial identifier and registry name. If not yet registered, name of intended registry	4
Trial registration: data set	#2b	All items from the World Health Organization Trial Registration Data Set	4
Protocol version	#3	Date and version identifier	31
Funding	#4	Sources and types of financial, material, and other support	31
Roles and responsibilities: contributorship	#5a	Names, affiliations, and roles of protocol contributors	30

For peer review only

Roles and responsibilities: sponsor contact information	#5b	Name and contact information for the trial sponsor	31
Roles and responsibilities: sponsor and funder	#5c	Role of study sponsor and funders, if any, in study design; collection, management, analysis, and interpretation of data; writing of the report; and the decision to submit the report for publication, including whether they will have ultimate authority over any of these activities	31-32
Roles and responsibilities: committees	#5d	Composition, roles, and responsibilities of the coordinating centre, steering committee, endpoint adjudication committee, data management team, and other individuals or groups overseeing the trial, if applicable (see Item 21a for data monitoring committee)	22-23
**Introduction**			
Background and rationale	#6a	Description of research question and justification for undertaking the trial, including summary of relevant studies (published and unpublished) examining benefits and harms for each intervention	6-10
Background and rationale: choice of comparators	#6b	Explanation for choice of comparators	14
Objectives	#7	Specific objectives or hypotheses	10-12
Trial design	#8	Description of trial design including type of trial (eg, parallel group, crossover, factorial, single group), allocation ratio, and framework (eg, superiority, equivalence, non-inferiority, exploratory)	12
**Methods: Participants, interventions, and outcomes**			
Study setting	#9	Description of study settings (eg, community clinic, academic hospital) and list of countries where data will be collected. Reference to where list of study sites can be obtained	12
Eligibility criteria	#10	Inclusion and exclusion criteria for participants. If applicable, eligibility criteria for study centres and individuals who will	12-13

For peer review only

		perform the interventions (eg, surgeons, psychotherapists)	
Interventions: description	#11a	Interventions for each group with sufficient detail to allow replication, including how and when they will be administered	14
Interventions: modifications	#11b	Criteria for discontinuing or modifying allocated interventions for a given trial participant (eg, drug dose change in response to harms, participant request, or improving / worsening disease)	14
Interventions: adherance	#11c	Strategies to improve adherence to intervention protocols, and any procedures for monitoring adherence (eg, drug tablet return; laboratory tests)	20
Interventions: concomitant care	#11d	Relevant concomitant care and interventions that are permitted or prohibited during the trial	12-13
Outcomes	#12	Primary, secondary, and other outcomes, including the specific measurement variable (eg, systolic blood pressure), analysis metric (eg, change from baseline, final value, time to event), method of aggregation (eg, median, proportion), and time point for each outcome. Explanation of the clinical relevance of chosen efficacy and harm outcomes is strongly recommended	23
Participant timeline	#13	Time schedule of enrolment, interventions (including any run-ins and washouts), assessments, and visits for participants. A schematic diagram is highly recommended (see Figure)	15-22
Sample size	#14	Estimated number of participants needed to achieve study objectives and how it was determined, including clinical and statistical assumptions supporting any sample size calculations	24
Recruitment	#15	Strategies for achieving adequate participant enrolment to reach target sample size	15
**Methods: Assignment of interventions (for controlled trials)**			
Allocation: sequence generation	#16a	Method of generating the allocation sequence (eg, computer-generated random numbers), and list of any factors for stratification. To reduce predictability of a random sequence, details of any planned restriction (eg, blocking) should be provided in a separate document that is unavailable to those who enrol participants or assign interventions	14-15

For peer review only

Allocation concealment mechanism	#16b	Mechanism of implementing the allocation sequence (eg, central telephone; sequentially numbered, opaque, sealed envelopes), describing any steps to conceal the sequence until interventions are assigned	14-15
Allocation: implementation	#16c	Who will generate the allocation sequence, who will enrol participants, and who will assign participants to interventions	14-15
Blinding (masking)	#17a	Who will be blinded after assignment to interventions (eg, trial participants, care providers, outcome assessors, data analysts), and how	14-15
Blinding (masking): emergency unblinding	#17b	If blinded, circumstances under which unblinding is permissible, and procedure for revealing a participant's allocated intervention during the trial	14-15
**Methods: Data collection, management, and analysis**			
Data collection plan	#18a	Plans for assessment and collection of outcome, baseline, and other trial data, including any related processes to promote data quality (eg, duplicate measurements, training of assessors) and a description of study instruments (eg, questionnaires, laboratory tests) along with their reliability and validity, if known. Reference to where data collection forms can be found, if not in the protocol	15-22
Data collection plan: retention	#18b	Plans to promote participant retention and complete follow-up, including list of any outcome data to be collected for participants who discontinue or deviate from intervention protocols	15-22
Data management	#19	Plans for data entry, coding, security, and storage, including any related processes to promote data quality (eg, double data entry; range checks for data values). Reference to where details of data management procedures can be found, if not in the protocol	24-25
Statistics: outcomes	#20a	Statistical methods for analysing primary and secondary outcomes. Reference to where other details of the statistical analysis plan can be found, if not in the protocol	24
Statistics: additional analyses	#20b	Methods for any additional analyses (eg, subgroup and adjusted analyses)	24

For peer review only

Statistics: analysis population and missing data	#20c	Definition of analysis population relating to protocol non-adherence (eg, as randomised analysis), and any statistical methods to handle missing data (eg, multiple imputation)	24
**Methods: Monitoring**			
Data monitoring: formal committee	#21a	Composition of data monitoring committee (DMC); summary of its role and reporting structure; statement of whether it is independent from the sponsor and competing interests; and reference to where further details about its charter can be found, if not in the protocol. Alternatively, an explanation of why a DMC is not needed	22-23
Data monitoring: interim analysis	#21b	Description of any interim analyses and stopping guidelines, including who will have access to these interim results and make the final decision to terminate the trial	25
Harms	#22	Plans for collecting, assessing, reporting, and managing solicited and spontaneously reported adverse events and other unintended effects of trial interventions or trial conduct	23-24
Auditing	#23	Frequency and procedures for auditing trial conduct, if any, and whether the process will be independent from investigators and the sponsor	25
**Ethics and dissemination**			
Research ethics approval	#24	Plans for seeking research ethics committee / institutional review board (REC / IRB) approval	26
Protocol amendments	#25	Plans for communicating important protocol modifications (eg, changes to eligibility criteria, outcomes, analyses) to relevant parties (eg, investigators, REC / IRBs, trial participants, trial registries, journals, regulators)	26
Consent or assent	#26a	Who will obtain informed consent or assent from potential trial participants or authorised surrogates, and how (see Item 32)	19
Consent or assent: ancillary studies	#26b	Additional consent provisions for collection and use of participant data and biological specimens in ancillary studies, if applicable	26
Confidentiality	#27	How personal information about potential and enrolled participants will be collected, shared, and maintained in order to protect confidentiality before, during, and after the trial	26

For peer review only

Declaration of interests	#28	Financial and other competing interests for principal investigators for the overall trial and each study site	30
Data access	#29	Statement of who will have access to the final trial dataset, and disclosure of contractual agreements that limit such access for investigators	25
Ancillary and post trial care	#30	Provisions, if any, for ancillary and post-trial care, and for compensation to those who suffer harm from trial participation	
Dissemination policy: trial results	#31a	Plans for investigators and sponsor to communicate trial results to participants, healthcare professionals, the public, and other relevant groups (eg, via publication, reporting in results databases, or other data sharing arrangements), including any publication restrictions	22
Dissemination policy: authorship	#31b	Authorship eligibility guidelines and any intended use of professional writers	30
Dissemination policy: reproducible research	#31c	Plans, if any, for granting public access to the full protocol, participant-level dataset, and statistical code	31
**Appendices**			
Informed consent materials	#32	Model consent form and other related documentation given to participants and authorised surrogates	26
Biological specimens	#33	Plans for collection, laboratory evaluation, and storage of biological specimens for genetic or molecular analysis in the current trial and for future use in ancillary studies, if applicable	22

None The SPIRIT Explanation and Elaboration paper is distributed under the terms of the Creative Commons Attribution License CC-BY-NC. This checklist can be completed online using https://www.goodreports.org/, a tool made by the EQUATOR Network in collaboration with Penelope.ai

For peer review only

BMJ Open

Protocol for a randomised, multicentre, four-arm, double-blinded, placebo-controlled trial to assess the benefits and safety of Iron supplementation with Malaria chemoprevention to children in Malawi - IRMA trial

Journal:	*BMJ Open*
Manuscript ID	bmjopen-2022-069011.R1
Article Type:	Protocol
Date Submitted by the Author:	15-Feb-2023
Complete List of Authors:	Mwangi, Martin; Training and Research Unit of Excellence (TRUE), Nutrition and Infectious Diseases ; The Micronutrient Forum, Healthy Mothers Healthy Babies Consortium<br>Mzembe, Glory; Training and Research Unit of Excellence (TRUE), Nutrition and Infectious Diseases ; Kamuzu University of Health Sciences, Public Health<br>Ngwira, Chikondi ; Training and Research Unit of Excellence (TRUE), Nutrition and Infectious Diseases ; Liverpool School of Tropical Medicine<br>Vokhiwa, Maclean; Training and Research Unit of Excellence (TRUE), Nutrition and Infectious Diseases<br>Kapulula, Mayamiko; Training and Research Unit of Excellence (TRUE), Nutrition and Infectious Diseases<br>Larson, Leila; University of South Carolina Arnold School of Public Health, Department of Health Promotion, Education, and Behaviour<br>Braat, Sabine ; University of Melbourne School of Population and Global Health, Centre for Epidemiology and Biostatistics; Walter and Eliza Hall Institute of Medical Research<br>Harding, Rebecca; The Walter and Eliza Hall Institute of Medical Research,<br>McLean, Alistair; Walter and Eliza Hall Institute of Medical Research; The University of Melbourne, Department of Infectious Diseases<br>Hamadani, Jena; International Centre for Diarrhoeal Disease Research<br>Biggs, Beverley-Ann ; The University of Melbourne, Medicine<br>Ataíde, Ricardo; The Walter and Eliza Hall Institute of Medical Research; The University of Melbourne, Department of Infectious Diseases at the Peter Doherty Institute of Infection and Immunity<br>Phiri, Kamija; Training and Research Unit of Excellence (TRUE), Nutrition and Infectious Diseases ; Kamuzu University of Health Sciences, Public Health<br>Pasricha, Sant-Rayn; Walter and Eliza Hall Institute of Medical Research,
**Primary Subject Heading**:	Public health
Secondary Subject Heading:	Haematology (incl blood transfusion)
Keywords:	Anaemia < HAEMATOLOGY, Infection control < INFECTIOUS DISEASES, PUBLIC HEALTH, Nutrition < TROPICAL MEDICINE

For peer review only - http://bmjopen.bmj.com/site/about/guidelines.xhtml

Protocol for a randomised, multicentre, four-arm, double-blinded, placebo-controlled trial to assess the benefits and safety of Iron supplementation with Malaria chemoprevention to children in Malawi - IRMA trial

Authors

*Martin N. Mwangi[1,2], *Glory Mzembe[1,3], Chikondi C. Ngwira[1], Maclean Vokhiwa[1,3], Mayamiko D. Kapulula[1], Leila M. Larson[4], Sabine Braat[5,6,7], Rebecca Harding[7], Alistair R.D. McLean[6,7], Jena D. Hamadani[8], Beverley-Ann Biggs[5,9], Ricardo Ataíde[5,7], Kamija S. Phiri[1,3], Sant-Rayn Pasricha [7,9,10]

*Joint first authors

Corresponding author:

Martin N. Mwangi, Training and Research Unit of Excellence (TRUE), 1 Kufa Road, Post Office Box 30538, Blantyre, Malawi. Email: mmwangi@true.mw

Author Institutions

1. Training and Research Unit of Excellence (TRUE), Blantyre, Malawi.
2. The Micronutrient Forum, Healthy Mothers Healthy Babies Consortium, Washington DC, USA.
3. Department of Public Health, School of Public and Global Health, Kamuzu University of Health Sciences, Blantyre, Malawi.
4. Department of Health Promotion, Education, and Behaviour, Arnold School of Public Health, University of South Carolina, Columbia, South Carolina, USA.
5. Department of Infectious Diseases, University of Melbourne, at the Peter Doherty Institute for Infection and Immunity, Melbourne, Australia.

For peer review only - http://bmjopen.bmj.com/site/about/guidelines.xhtml

6. Centre for Epidemiology and Biostatistics, Melbourne School of Population and Global Health, The University of Melbourne, Melbourne, Australia.
7. Population Health and Immunity Division, The Walter and Eliza Hall Institute of Medical Research, Melbourne, Australia.
8. International Centre for Diarrhoeal Disease Research, Bangladesh, Dhaka, Bangladesh.
9. DDiagnostic Haematology, The Royal Melbourne Hospital; and Clinical Haematology, Melbourne, Australia.
10. Department of Medical Biology, Faculty of Medicine, Dentistry and Health Sciences, The University of Melbourne, Melbourne, Australia.

Word count: 4750. (Abstract) 298 words. *(excluding title page, abstract, references, figures and tables)*

For peer review only

For peer review only - http://bmjopen.bmj.com/site/about/guidelines.xhtml

ABSTRACT

Introduction: Approximately 40% of children 6-59 months of age worldwide are anaemic. Iron-containing multiple micronutrient powders (MNPs) and iron supplements (syrup/drops) are used to combat anaemia in children in different parts of the world. However, evidence for functional benefits of iron supplementation in children is scarce, and potential risks remain poorly defined, particularly concerning diarrhoea and malaria. The trial aims to determine if: a) the efficacy of iron supplements or MNPs (containing iron) given with malaria chemoprevention is superior to malaria chemoprevention alone, or b) if the efficacy of malaria chemoprevention alone is superior to placebo on child cognitive development.

Methods and Analysis: IRMA is a four-arm, parallel-group, double-blinded, placebo-controlled, triple-dummy, randomised trial in Southern Malawi. The study recruits 2,168 infants aged six months, with an intervention period of six months and a post-intervention period of a further six months. Children are randomised into four arms: (a) No intervention (placebo); (b) malaria chemoprevention only; (c) MNPs and Malaria chemoprevention; and (d) Iron syrup and malaria chemoprevention. The primary outcome, cognitive development (CogCS), is measured at the end of the six months intervention. Secondary outcomes include CogCS at a further six months post-intervention, motor, language and behavioural development, physical growth, and prevalence of anaemia and iron deficiency. Safety outcomes include incidence of malaria and other infections, and prevalence of malaria parasitaemia during and post-intervention period.

Ethics and dissemination: The trial is approved by the National Health Sciences Research Committee (#19/01/2213) (Malawi) and the Human Research Ethics Committee (WEHI: 19/012) (Australia). Written informed consent in the local language is obtained from each

For peer review only - http://bmjopen.bmj.com/site/about/guidelines.xhtml

participant before conducting any study-related procedure. Results will be shared with the local community and internationally with academic and policy stakeholders.

Protocol registration details: The protocol is registered with the Australian and New Zealand Clinical Trials Registry (ACTRN12620000386932).

Keywords: Anaemia, Infant, Infection, Iron Deficiency, Malawi, Malaria, Micronutrient Powders, randomised controlled trial.

Strengths and limitations of this study

- The trial setting represents malaria-endemic Sub-Saharan African countries where anaemia and malaria co-exist, which is an ideal place to study the benefits versus risks of iron supplementation (under malaria chemoprevention) to establish a generalisable clinical rationale.
- The participant screening and inclusion criteria involve all infants within the recruitment age range across a large geographical catchment area which enables the generalizability of the study results to most national programs in malaria-endemic countries.
- The trial compares the two major delivery modes for iron in young children: MNPs and iron syrup which will help identify the ideal and most effective iron source for children.
- The trial utilises a functional clinical health outcome – child cognitive development – as its primary outcome, ensuring the trial provides evidence of the functional relevance of universal iron interventions and malaria prevention.
- The trial is powered to detect a small but clinically relevant effect for the primary outcome of child cognitive development; the use of a post-intervention follow-up

For peer review only

For peer review only - http://bmjopen.bmj.com/site/about/guidelines.xhtml

period enables the assessment of both the immediate and medium-term impact of the intervention.

INTRODUCTION

Anaemia remains a critical global health problem affecting up to 39.8% of children aged 6 – 59 months worldwide and 60.2% in Africa.[1-3] In Malawi, 63% of children 6-59 months are anaemic, with a peak prevalence of 91% observed among children aged 9-11 months.[4] Iron deficiency accounts for most (60%) of the global burden of anaemia[5] but less so in malaria-endemic areas, where only 32% of childhood anaemia is iron responsive.[1]

World Health Organisation (WHO) anaemia prevention guidelines recommend two universal iron supplementation strategies for children, namely; 1) iron supplement drops or syrups (10-12.5mg of elemental iron) – given daily to children 6-24 months in areas where the prevalence of anaemia is 40% or higher;[6-8] and 2) iron-containing multiple micronutrient powders (MNPs): 10-12.5mg of iron – given daily over six months in areas where the prevalence of anaemia is 20% or higher.[6-8] However, no recommendation is given regarding the preferred approach. MNPs are the most common form of iron supplementation, with at least 58 countries reported to have implemented MNPs home fortification by 2021.[9] The rationale for universal iron supplementation is an understanding that iron supplementation would reduce childhood anaemia and improve child development and other functional health outcomes. However, we recently showed that in a rural South Asian population, neither universal distribution of oral iron supplements nor MNPs meaningfully improved immediate or medium-term child cognitive, language, motor, and other developmental outcomes, even though anaemia and iron deficiency were reduced.[10] However, these data were generated

For peer review only - http://bmjopen.bmj.com/site/about/guidelines.xhtml

from a trial in a non-malaria endemic part of South Asia, where the determinants of anaemia and infection risk are likely very different.

Benefits of universal iron supplementation programmes

Universal iron supplementation programs have shown benefits in haematological outcomes. A meta-analysis[11] and a Cochrane review[12] showed improvements in anaemia with daily iron supplementation in children. However, the effects on growth and development are less clear. Few studies have investigated the impact on cognitive development. Only 8 of the 33 studies included in the meta-analysis by Pasricha et al. assessed cognitive development, and no significant beneficial effect was seen (mean difference 1.65 [95% CI -0.63, 3.94]), but data were sparse.[11] Subgroup analysis did suggest a benefit of iron supplementation on cognitive development in iron-deficient children. However, this data came from only two studies.[13 14] Only one study in the Cochrane review assessed child development, showing that children receiving MNPs were more likely to walk independently at 12 months of age than those receiving no intervention.[15] There is also little evidence for a beneficial effect on growth;[11 12 16] however, data were limited.[11] WHO has acknowledged the need for further research into the functional outcomes of iron supplementation programs.[7]

Risks of iron supplementation

Whilst the evidence for functional benefits of iron supplementation is scarce, there has been emerging evidence of potential harm, particularly regarding increased malaria risk in endemic areas. The seminal Pemba RCT in Tanzania was stopped early due to increased death, hospitalisation and malaria in iron replete children receiving iron compared with placebo and called for revisions of universal iron supplementation recommendations.[17] In another RCT in Tanzania, multi-nutrient supplementation (including iron) increased the number of malaria

For peer review only - http://bmjopen.bmj.com/site/about/guidelines.xhtml

cases by 41% in iron-deficient children.[18] However, a 2016 Cochrane review found iron supplementation safe in areas with adequate malaria prevention and management strategies.[19] In contrast, there was an increased risk in areas where this was not the case.[19] The current recommendation is for universal iron supplementation in malaria-endemic regions with a high prevalence of anaemia only "in conjunction with public health measures to prevent, diagnose and treat malaria."[7]

Iron deficiency protects against malaria

There is evidence to suggest that iron deficiency is protective against malaria. A study of preschool children in Malawi found that iron-deficient children were at lower risk of clinical malaria and parasitaemia than iron-replete children.[20] *In vitro* studies have also demonstrated that *Plasmodium falciparum* parasites are less effective at invading iron deficient red cells and prefer invading reticulocytes (which are increased in the recovery phase of iron deficiency anaemia when iron supplementation is given).[21] Parasite invasion and growth were reduced in laboratory growth and invasion studies of *P. falciparum* using red cells from anaemic children.[22] The protective effect imparted by iron deficiency in both in vitro studies was reversed when iron supplementation was given.[21 22]

Anaemia in malaria-endemic areas is less iron-responsive

Malaria is a significant cause of severe anaemia, hospitalisation, and death in endemic areas.[23-25] Several aetiologies, including malaria, were identified in a study of Malawian children with severe anaemia. However, iron deficiency was not a common contributor.[23] Similarly, in a study in children from The Gambia and Tanzania,[26] 61.2% of children were anaemic, but only 13% had iron deficiency anaemia.[26] In keeping with this, the anaemia in these areas is less iron-responsive.[11 27] Without clear evidence of the neurodevelopmental

For peer review only

For peer review only - http://bmjopen.bmj.com/site/about/guidelines.xhtml

147 benefits of iron supplementation, iron interventions are likely to be extremely expensive public health interventions in terms of cost per DALY averted, especially in sub-Saharan Africa.[28]

150 **Malaria may be a risk factor for impaired cognitive development**

151 Despite few studies, the majority with relatively small sample sizes and varying methods of assessing cognitive function, evidence suggests that severe and uncomplicated malaria may be independent risk factors for cognitive impairment.[29] In addition, intermittent preventive therapy in preschool and school-aged children, has improved cognitive performance.[30 31]

155 **Intermittent preventive treatment to decrease malaria and its sequelae**

156 Intermittent Preventive Treatment in Children (IPTc) involves the empiric treatment of malaria at regular intervals and is a potential strategy to decrease malaria rates and sequelae. A Cochrane review that included seven RCTs evaluated the effect of chemoprophylaxis and intermittent treatment for malaria in preschool children in areas with seasonal malaria and showed a reduction in clinical malaria and moderate to severe anaemia.[32] Furthermore, in a meta-analysis, iron supplementation reduced the rate of anaemia by 30% in malaria-endemic areas, compared with 60% in non-endemic regions.[11]

163 **DP as chemoprevention**

164 Recommended treatment for *P. falciparum* malaria is with artemisinin-based combination therapy (ACT). DP is an ACT and is among WHO's five recommended first-line treatments.[33] DP with its long half-life, given monthly, is an attractive option for IPTc. In an RCT in children in Uganda using this regimen, DP reduced clinical malaria by 58% (compared with 28% for trimethoprim-sulfamethoxazole and 7% for sulfadoxine-pyrimethamine) and parasitaemia by

For peer review only - http://bmjopen.bmj.com/site/about/guidelines.xhtml

76%. IPTc with DP also reduced moderate-to-severe anaemia by 47%.[34] The administration of DP-IPTc during MNPs/iron supplementation may improve the safety of these interventions. However, there are concerns that malaria prevention may delay the development of immunity and increase malaria risk once IPTc is ceased.[32]

Iron deficiency and malaria co-exist in Malawi

Despite important gains made worldwide in malaria control since 2010, worldwide cases increased in 2016. In Malawi, there were a total of 5.2 million presumed and confirmed malaria cases in 2016, 231,970 more cases than the year prior, resulting from a gradual increase in cases since 2014.[35] In addition, the prevalence of malaria parasitaemia in children under five years increased from 28% in 2012 to 33% in 2014. These increases come despite increases in insecticide-treated bed net coverage.[36]

Both malaria and iron deficiency anaemia remain important causes of morbidity and mortality in Malawi. In Malawian children under five years of age, malaria is the leading cause of death,[37] and the prevalence of anaemia is 63%.[38] Addressing both of these conditions is challenging. Current WHO recommendations for improving anaemia in malaria-endemic regions are difficult to interpret and implement in countries like Malawi, where malaria preventive measures and treatment are present but by no means universal.

Why this trial is needed

Several fundamental questions concerning the benefits and safety of iron-intervention programs in malaria-endemic regions remain unanswered. The IRMA trial will assess functional health outcomes from iron supplementation, identify the ideal mode of iron delivery (MNPs versus iron syrup/drops), and determine whether adding IPT diminishes

For peer review only

For peer review only - http://bmjopen.bmj.com/site/about/guidelines.xhtml

potential risks. It will also allow evaluation of the effect of malaria chemoprevention alone on anaemia and child development.

OBJECTIVES

The primary objective is to determine a) if the efficacy of iron supplements or MNPs (containing iron) given with malaria chemoprevention is superior to malaria chemoprevention alone, or b) if the efficacy of malaria chemoprevention alone is superior to placebo, on child cognitive development (measured using locally adapted Bayley Scales of Infant and Toddler Development 3rd Edition (Bayley-III) Cognitive Composite Scores[39]) at the end of the 6-month intervention period.

The specific objectives are:

1. Determine if the efficacy of a) iron (as supplements or MNPs) given with malaria chemoprevention is superior to malaria chemoprevention alone or b) if the efficacy of malaria chemoprevention alone is superior to placebo on;
    i. child cognitive development, child motor, language, behavioural development, temperament, and physical growth at the end of the six months intervention and a further six months post-intervention;
    ii. anaemia (and haemoglobin concentration), iron deficiency (and ferritin) and iron deficiency anaemia after six months of intervention and six months post intervention.
2. Compare the effects of iron given as supplements to MNPs on the above outcomes after six months of intervention and a further six-months post-intervention.

For peer review only - http://bmjopen.bmj.com/site/about/guidelines.xhtml

3. Determine the safety of iron (as supplements or MNPs) given with malaria chemoprevention on the risk of malaria and other morbidities versus malaria chemoprevention alone or placebo during and post-intervention.
4. Determine the efficacy of malaria chemoprevention alone compared to placebo on clinical malaria, parasitaemia and malaria-specific clinic visits, and all-cause sick visits during and after intervention.
5. Determine the safety of iron (as supplements or MNPs) given with malaria chemoprevention compared to placebo on infection risk in young children, during and post-intervention.

Study design

IRMA is a four-arm parallel-group, double-blinded, placebo-controlled, triple-dummy, individually randomised trial comparing: (a) No intervention, (b) Malaria chemoprevention only, (c) MNPs + Malaria chemoprevention and (d) Iron syrup + Malaria chemoprevention at the end of the six months intervention and at a further 6-months post-intervention. The trial design is summarised in **Figure 1**.

Study settings and participants

The trial is set in Chikwawa district, in Southern Malawi, a rural area where >70% of children 6-12 months are anaemic,[40] and where 33% of children under five years old have *Plasmodium falciparum* parasitaemia.[41] Four health facilities within the district are hosting the trial, namely: Chikwawa District Hospital, Mapelera Health Centre, Mfera Health Centre and Makhwira Health Centre. These are the primary health facilities where children attend routine under-five clinics and sick visits. Our target participants are all children aged six months residing in these health facilities' catchment area.

For peer review only - http://bmjopen.bmj.com/site/about/guidelines.xhtml

Eligibility criteria

The criteria to select participants considers participants' safety, the generalizability to national programs, and the feasibility of the trial. Children are randomly assigned to a study intervention group only if they meet all the inclusion and none of the exclusion criteria.

Inclusion criteria

1. Infant aged six months (+/- 14 days) at randomisation.
2. Availability of a legally acceptable guardian/representative and providing written consent on the participant’s behalf.

Exclusion criteria

1. Severe anaemia (Hb<7g/dL).
2. Clinical signs of current infective illness (respiratory infection, diarrhoea, malaria); however, children will be screened after recovery and recruited if they meet the age eligibility criteria at the second screening.
3. Severe malnutrition (weight-for-length z-score<-3) or MUAC < 11.5 cm.
4. Established haemoglobinopathy diagnosis (e.g., sickle cell disease, beta thalassaemia major, HbE-beta thalassaemia).
5. Known congenital anomaly, developmental disorder, or severe developmental delay.
6. Children with physical or behavioural problems that will make it impossible to undertake trial assessments.
7. Children of multiple births, e.g., twins.
8. Children currently consuming iron supplements or MNPs, e.g., from the National MNPs programme.

For peer review only - http://bmjopen.bmj.com/site/about/guidelines.xhtml

9. Enrolment in another trial.

Trial Interventions

Participants are randomly assigned to receive one of the following interventions:

1) **No intervention (Placebo DP *plus* Placebo MNPs *plus* placebo iron syrup)**: Placebo DP (Guilin Pharmaceutical company, China)**:** three consecutive days every four weeks for six months *plus* Placebo MNPs (maltodextrin vehicle only; DSM Nutritional Products, South Africa) daily for six months *plus* placebo iron syrup (ACME Laboratories Ltd., Bangladesh) daily for six months.
2) **Malaria chemoprevention only (Active DP *plus* placebo MNPs *plus* placebo iron syrup)**: Active DP (20mg dihydroartemisinin/160mg piperaquine; Guilin Pharmaceutical company, China) dosed by body weight (2/16mg/Kg) for three consecutive days every four weeks for six months *plus* placebo MNPs daily for six months, *plus* daily placebo iron syrup for six months.
3) **MNP + malaria chemopevention (Active DP *plus* active MNPs *plus* placebo iron syrup):** Active DP as above, *plus* daily active MNPs containing 10.0mg iron and 14 other micronutrients (see supplementary material 1); DSM Nutritional Products, South Africa, *plus* placebo iron syrup daily for six months.
4) **Iron syrup + malaria chemoprevention (Active DP *plus* placebo MNPs *plus* active iron syrup):** Active DP as above, *plus* placebo MNPs, *plus* active iron syrup containing 10.0mg ferrous sulphate (ACME Laboratories Ltd., Bangladesh) daily for six months.

Randomisation, allocation concealment, and blinding

Participants are randomly allocated to one of the four study arms with 1:1:1:1 allocation via a computer-generated randomisation schedule of randomly permuted blocks stratified by sex

For peer review only - http://bmjopen.bmj.com/site/about/guidelines.xhtml

For peer review only

and health facility/study sites (Chikwawa District Hospital, Mapelera health centre, Mfera health centre and Makhwira Health Centre) to achieve a balance between the intervention arms. An independent statistician from the University of Melbourne (Australia) generated the randomisation list.

An independent Trial Pharmacist pre-packed and pre-labelled participant packs containing all three investigational products based on the randomisation list. Each participating child who meets the inclusion criteria is sequentially allocated to the next available study number indicated on the randomisation list. The participant is then given a pre-labelled pre-packed Ziplock bag containing the study medication corresponding to the study number assigned to them. Hence, participants and their families and all study team members involved in collecting and analysing outcome data, including field workers, developmental assessors, laboratory scientists, statisticians, and investigators, are blinded to the treatment allocation until the database is locked and before unblinding.

Recruitment and Visits

Patient and Public Involvement

All levels of the local leadership (head men/women, sub-chiefs, chiefs, Community Health Action Groups (CHAGS), Health Surveillance Assistants (HSAs) and District Health Assembly (DHA)) are continuously engaged by the trial's Community Engagement Team (CET). CHAGs and HSAs were engaged to ensure that we reach the whole community via it's resident health "gate-keepers". Additionally, the CET conducted community sensitisation meetings to explain the research objectives, what it means to participate, benefits for the individual or the community, and overall contribution to science. Finally, community members are regularly invited to contribute ideas that could help improve the success of the research thereby

For peer review only

For peer review only - http://bmjopen.bmj.com/site/about/guidelines.xhtml

increasing the probability of success.

Screening and enrolment of participants

Study recruitment opened on 17 April 2020. Study activities are as detailed below (also detailed in supplementary material 2).

Pre-screening

We conducted a house-to-house enumeration (census) of all potential participants (children under six months of age) living in the target area and generated a study population database. New births are identified and reported by HSAs or registered at the health facilities' under-five clinics throughout the trial recruitment period and added to the database. Research Assistants visit all children approaching six months of age in the community. After taking written informed consent, the team reviews the child's medical history to determine potential eligibility. Eligible children are invited to visit a selected health facility for further screening.

Screening

On a pre-determined day, the child, accompanied by their parent(s) or legal guardian, visit the selected health centre. HemoCue Hb301+ (Angelholm, Sweden) is used to assess capillary Hb level to exclude severely anaemic children. Additionally, anthropometric measurements: weight and length (used to calculate weight-for-length z-scores[42]) and mid-upper arm circumference (MUAC) are performed to exclude severely malnourished children. Severely anaemic and severely malnourished children are referred to the routine health system for further investigation and treatment.

Baseline and Enrolment (at six months of age (+/- 14 days))

For peer review only - http://bmjopen.bmj.com/site/about/guidelines.xhtml

A second, more detailed consent form for participation in the study is signed, and a study identification number is assigned. Randomisation is performed after the following baseline evaluations (see **supplementary material 2**) are carried out and necessary information collected: demographic information, socioeconomic status (using the Malawi Demographic Health Survey (MDHS) standard of living scale), medical history, family care indicators[43]/index (FCI), feelings questionnaire,[44] cost survey (household out of pocket health care costs), food security questionnaire,[45] dietary diversity questionnaire,[46] developmental screening using Caregiver Reported Early Development Instrument (CREDI),[47] anthropometric measurements (weight, length, MUAC and head circumference), and anthropometric measurements for the mother (weight, height). A venous blood sample (3 mL), for haemoglobin testing, determination of iron indices (serum – for ferritin, CRP, and AGP), thick and thin malaria slides for malaria microscopy testing (to be conducted at the completion of the trial) and filter paper for plasmodium PCR, is collected from the child. The mother is given educational information regarding the use of study medications, administration of the first dose of DP and instructions on how to administer day two and day three DP treatments, administration of the first dose of iron syrup, use of MNPs and advice on future visits.

Subsequent study visits

Adherence assessment

Fortnightly 'home visits' by experienced research assistants to the participants' homes in the community are done to assess the following: 1) documentation of episodes of infection (rate, days affected), 2) recording any adverse events/effects, 3) documentation of details of unplanned hospital and healthcare facility attendance for example to a doctor, traditional healer, clinic or hospital, 4) provision of educational information to participants on the correct

For peer review only - http://bmjopen.bmj.com/site/about/guidelines.xhtml

use of the study interventions and encouraging adherence to study interventions, 5) adherence monitoring (measured from parental questioning along with assessment of residual MNPs and syrup) and 6) advice on future visits.

Monthly DP issuance

The research assistants issue the investigational products monthly during a home visit. The following activities (including data collection) are undertaken: issuance of the study interventions according to study protocol and adherence monitoring (measured from parental questioning along with assessment of residual MNPs and syrup). In addition, unused investigational products are collected.

Study midline visit (at 12 months of age +/- 14 days)

The participating child and their parent/ legal guardian attend the health facility at the end of the six months intervention for developmental assessments using locally adapted Bayley Scales of Infant and Toddler Development, Wolke's Behavioural Rating (WBR), and Child Temperament using the Bates' infant characteristics questionnaire. Anthropometric measurements are conducted on both the child and the mother. Venous blood (3 mL) for haemoglobin testing, determination of iron indices, malaria microscopy (to be conducted at the completion of the trial) and malaria filter paper for future PCR analyses is collected from the child.

Monthly malaria infection testing

Research assistants visit the homes of participants once per month after completion of the midline visit for assessment of episodes of infection (rate, number of days affected) in the previous one month and documentation of unplanned hospital and/or healthcare facility

For peer review only

For peer review only - http://bmjopen.bmj.com/site/about/guidelines.xhtml

370 attendance (details of any interim visit to doctor, clinic or hospital visits in the previous
371 month) and record of healthcare utilisation.

372 *Endline visit (at 18 months of age (-2 weeks or +6 weeks)*

373 The procedures are similar to the midline visit described above and in **supplementary**
374 **material 2.**

375 *Unscheduled visits*

376 Mothers or legal guardians are asked to attend the research site/clinic when their child is
377 symptomatically unwell. They are managed according to national standard treatment
378 guidelines by a trained healthcare provider. Blood samples for malaria rapid diagnostic test
379 (RDT) and blood culture are taken if clinically indicated. The clinical diagnosis of the
380 unscheduled visit is recorded in the participant's record and captured on the study's interim
381 visit Case Report Form (CRF) and adverse event form. If the child requires inpatient care, they
382 are referred to the Chikwawa district hospital.

383 **Laboratory Procedures**

384 A capillary blood sample is collected by finger prick from all age-eligible participants to
385 measure the haemoglobin concentration by HemoCue (HemoCue 301+, Angelholm, Sweden)
386 and *Plasmodium* infection by rapid diagnostic test (mRDTs); (SD Bioline Malaria Ag Pf/Pan®,
387 Standard Diagnostics) at baseline. In addition, up to 3 mL venous blood sample are collected
388 from all enrolled participants at baseline, midline, endline and unscheduled visits (if
389 necessary)) for haemoglobin testing, determination of iron indices (serum – for ferritin, CRP,
390 and AGP), thick and thin malaria slides for malaria microscopy testing and filter paper for
391 *Plasmodium* PCR. Families are encouraged to permit the collection of venous blood for venous

For peer review only

haemoglobin and iron and inflammation biomarkers at all time points; however, if a family refuses venous blood collection or if collection was unsuccessful, data for the primary outcome and other non-laboratory secondary outcomes will still be collected.

Data Safety and Monitoring Committee (DSMC)

An independent DSMC comprised of international experts in clinical trials, child health, infectious diseases, epidemiology, and statistics was set up to review regularly, safety data of the ongoing trial. The DSMC may recommend to the sponsor and investigators whether to continue, modify, or terminate the trial on ethical grounds.

Outcomes

The primary outcome

Cognitive development: assessed by the differences in Bayley-III Cognitive Composite Score (CogCS) between intervention arms measured after six months intervention (midline) and 12 months post-intervention (endline). The Bayley-III is a validated index of child development that can be administered in ~0.5-1 hour and comprises cognitive, motor, and language development scales. Assessors are specially trained to administer the tests, and inter-rater reliability is established before starting data collection. In addition, we perform 10% inter-observer testing to monitor inter-rater reliability of the measure over the study period.

Secondary outcomes

Secondary outcomes include: motor and language development assessed by Bayley-III Motor (MotCS) and Language (LangCS) Composite Scores (+6m, +12m), behaviour assessed by WBR, temperament, growth assessed by weight-for-age z-score, length-for-age z-score, weight-for-length z-score, head circumference z-score, anaemia (Hb<11g/dL), haemoglobin

For peer review only - http://bmjopen.bmj.com/site/about/guidelines.xhtml

For peer review only

concentration, Iron Deficiency (Ferritin<12ng/uL adjusted for inflammation assessed by CRP or AGP), ferritin concentration, and Iron Deficiency Anaemia.

Safety will be assessed by prevalence of malaria parasitaemia and C-reactive protein (CRP) and inflammation (CRP>5mg/L) at the end of the intervention and a further 6 months post intervention; episodes of caregiver reported symptoms (e.g. fever, diarrhoea, vomiting, cough) determined by the biweekly and monthly morbidity questionnaire; episodes of unplanned clinic visits and hospitalisations and deaths.

Detection and reporting of Adverse Events and Serious Adverse Events

Non-serious Adverse Events (AEs) and serious adverse events (SAEs) are collected from the time consent is given until the participant completes the study (the final visit or withdrawal). These are identified through spontaneous reports by the participant, unplanned visits to the research site or any participating health centres, observation by the study staff, and standard questioning at each visit (biweekly during the intervention period and monthly during the postintervention period. All SAEs are reported to the sponsor and the appropriate ethics committee, whether or not they are considered causally related to the investigational products.

Sample size

The primary objective will be met if either i) MNPs+DP is superior to DP alone, or ii) Iron+DP alone is superior to DP alone or iii) DP alone is superior to placebo on the Bayley-III CogCS immediately after completion of the intervention (midline). A previous study in 2853 Bangladeshi children showed that a 0.2 standardised mean difference (SMD) in Bayley-II Mental Development Index at seven months translates to a 0.7 SMD at 18 months and a 1.2

For peer review only

For peer review only - http://bmjopen.bmj.com/site/about/guidelines.xhtml

436 SMD at 64 months age.[48] Therefore, preventing a loss of at least 0.2 SMD may produce magnified long-term developmental benefits as manifestations of brain dysfunction become more evident. A total sample size of 2168 infants (542 per group) will provide 90% power to detect an effect size of 0.25 SMD, assuming a 20% dropout and a two-sided alpha of 1.67% (Bonferroni adjustment for three primary between-group comparisons).

Statistical Analysis Plan

A stand-alone statistical analysis plan will be detailed before breaking the blind. This plan will detail all principles and precise methods of analysis, including primary and secondary treatment effects of interest; alternative analyses if model assumptions do not hold; and additional analyses. Participants will be analysed according to their randomised treatment group for efficacy objectives and actual treatment group for safety objectives. The primary outcome of Bayley-III CogCS will be analysed using a mixed-model repeated measures (MMRM) analysis including in the model treatment group, time-point, and group by time-point interaction. The stratification variables sex and site will be included in all models as main effects. In addition, the CREDI variable (collected at baseline) will be included as a main effect in the models for childhood development as it is considered prognostic for childhood development outcomes. Secondary repeated continuous outcomes (e.g., growth outcomes) will be analysed similarly to Bayley-III CogCS. The outcome may be appropriately transformed before fitting the analysis model if considered skewed (e.g., log-transformation for ferritin). Secondary repeated binary outcomes (e.g., haematologic outcomes) will be analysed using a log-binomial model and account for the repeated measures with a random intercept for participant. Safety (e.g., Aes, infections, hospital attendance) will be summarised during the intervention and post-intervention periods separately and compared between the groups.

For peer review only - http://bmjopen.bmj.com/site/about/guidelines.xhtml

Analysis of binary repeated outcome safety data (e.g., inflammation) and continuous repeated outcome safety data (e.g., CRP) will be analysed similarly to the secondary outcomes detailed above. Episode safety data (e.g., caregiver reported diarrhoea episodes) will be analysed using negative binomial regression models. Subgroup analyses (e.g., sex, site, iron deficiency) will be performed to explore the heterogeneity of the results independent of the study findings.

Data Management

Data collected from the subjects are recorded in digital Case Report Forms (CRFs) using Redcap software (Vanderbilt University, United States) on tablets. Relevant hard copy patient hospital files are scanned for reference and stored digitally, and securely. The trial is independently monitored by an external monitoring service. No interim analysis is planned.

ETHICS AND DISSEMINATION

The National Health Science Research Committee, Malawi, and the Walter and Eliza Hall Institute of Medical Research Human Research Ethics Committee, Australia, approved this study. The Malawian Pharmacy and Medicines Regulatory Authority (PMRA) also approved the trial. Additionally, the protocol is registered with the Australian and New Zealand Clinical Trials Registry (ACTRN12620000386932). Protocol modifications are reported to the ethics committees, regulatory authorities in Malawi, investigators, trial participants and trial registries. Written informed consent in the local language is obtained from each participant before conducting any study-related procedure. In the event where the participant is illiterate, the informed consent form is read out loudly to the potential participant in the presence of an impartial witness. The potential participant thumbprints the consent form if they agree to participate in the study and the witness signs to indicate he/she witnessed the

For peer review only

For peer review only - http://bmjopen.bmj.com/site/about/guidelines.xhtml

whole consenting process. The results will be presented and shared with the local community that hosted the research and to the international forum. We will publish in peer-reviewed scientific journals and report to relevant policymaking bodies like the Malawi Ministry of Health.

DISCUSSION

The IRMA trial will provide valuable evidence on the immediate and medium-term impacts of iron interventions (iron supplements and MNPs) and malaria prevention with DP on critical functional outcomes, most importantly, child cognitive development. These findings will complement high-quality data recently reported in South Asia to provide generalisable evidence for or against universal iron interventions across sub-Saharan Africa and other malaria-endemic settings.[10]

More than two-thirds of African children aged 6-59 months are anaemic. Preventing anaemia is considered important within the first 1000 days and aligns with WHO policy for children 6-23m of age,[49] especially because the central nervous system is rapidly developing and is highly vulnerable. The prevalence of iron deficiency in children across Africa has been shown to increase from birth to approximately one year, with a peak prevalence at 9-12 months of age, and then a decrease with increasing age. Iron interventions should thus target the critical under one year period.[50]

Recent clinical trials have indicated that the combination of Intermittent preventive treatment of malaria in infants (IPTi) and malaria vaccine (RTS<S/ $AS01_E$) provides much greater protection from malaria attacks in children compared to IPTi or RTS, S/$AS01_E$ given

For peer review only

For peer review only - http://bmjopen.bmj.com/site/about/guidelines.xhtml

alone.[51] Combining iron interventions with IPTi, would thus be optimal for preventing both malaria and anaemia in young children in this setting.

The study's strengths include the design with cognitive development as the primary functional outcome, the large sample size with the power to detect a small effect size, and the triple dummy design, which minimizes the risk of bias. The presumed benefit of cognitive development is the key rationale for iron preventive interventions in children. Limitations include routine six monthly interval malaria microscopy testing, which is not a reliable test for the prevalence of malaria parasitaemia compared to a monthly interval. However, the strong surveillance systems on clinical symptoms (biweekly home visits and phone calls) ensured that all clinical malaria cases were picked by ensuring that all children with fever and other symptoms could access hospital care and get a rapid malaria test done.

For peer review only

For peer review only - http://bmjopen.bmj.com/site/about/guidelines.xhtml

Author Contributions:

MNM drafted the initial study protocol. MNM and GM supervised the trial's implementation and wrote the protocol paper's first draft. CCN and MDK coordinated the implementation of the study. JDH, MV and LML planned for the measurement of the primary outcome. SB, RH, and ARDM wrote the statistical analysis plan. MNM and RA provided daily scientific input during the implementation of the trial. B-AB, KSP, and S-RP conceived the trial, sought funding, and provided senior scientific oversight in the conduct of the research. All authors read and approved the final manuscript.

Funding statement: This work is supported by the National Health and Medical Research Council (NHMRC), Australia, grant numbers GNT1141185 and GNT1159151. SP is funded by NHMRC GNT1158696 and GNT2009047.

Competing interests statement.

None to declare.

Acknowledgements:

We thank all the participants of the IRMA trial - including parents or legal guardians - especially during the challenging Covid-19 period. We thank the ethics and regulatory committees mentioned in the manuscript for their extensive review and approval of the study protocol and operational documents. We acknowledge the support from the Malawi government, the Chikwawa District Health Office, including the health management teams of Chikwawa District Hospital, Makhwira, Mapelera, and Mfera Health Centres. We thank the Shire River East-and-West bank communities for hosting the study and continuously bearing with the extensive study procedures, especially the conduct of the two censuses to determine

For peer review only

For peer review only - http://bmjopen.bmj.com/site/about/guidelines.xhtml

the extent of the source population. Finally, the study would not be possible without the support of the over 100 staff members (in Malawi and Australia) who contribute their time and expertise to ensure its success. We say thank you to all.

Data Statement

All relevant data are within the paper and its supporting information files.

Protocol version

IRMA Trial protocol version 4.0 dated July 25, 2022

Ethics and regulatory approvals

Ethical approval was granted by the National Health Sciences Research Committee (#19/01/2213) in Malawi and the Human Research Ethics Committee (WEHI: 19/012) in Melbourne, Australia. In addition, the Malawi Pharmacy and Medicines Regulatory Authority (PMRA) reviewed and approved the study and the purchase and importation of the investigational products to Malawi (clinical trial certificate number PMPB/CTRC/IV/06062019111).

Trial Sponsor

Training and Research Unit of Excellence (TRUE), 1 Kufa Road, Mandala. P.O. Box 30538, Chichiri, Blantyre 3, Malawi. Email: true@true.mw

Roles and responsibilities of sponsor and funder:

The sponsor and funder have no role in the study design. They will have no part or ultimate authority in the collection, management, analysis, and interpretation of data, report writing, and the decision to submit the report for publication.

For peer review only - http://bmjopen.bmj.com/site/about/guidelines.xhtml

Figure heading, caption and legend

Figure 1

Figure caption and legend: *IRMA Trial schema. Overall display of IRMA activities. MNP - Iron-containing multiple micronutrient powders; DP - Dihydroartemisinin-piperaquine; Intervention – issued monthly, from enrolment at age six months to 12 months of age. Primary endpoint – Bayley's assessment conducted at the trial midline; Cognitive assessments – Bayley Scales of Infant and Toddler Development 3rd Edition.*

For peer review only

For peer review only - http://bmjopen.bmj.com/site/about/guidelines.xhtml

References

1. World Health Organization. The global prevalence of anaemia in 2011. Geneva, Switzerland: WHO, 2015.
2. Stevens GA, Paciorek CJ, Flores-Urrutia MC, et al. National, regional, and global estimates of anaemia by severity in women and children for 2000-19: a pooled analysis of population-representative data. *Lancet Glob Health* 2022;10(5):e627-e39. doi: 10.1016/s2214-109x(22)00084-5 [published Online First: 2022/04/16]
3. World Health Organization. WHO Global anaemia estimates, 2021 Edition. Global anaemia estimates in women of reproductive age, by pregnancy status, and in children aged 6-59 months. Geneva, Switzerland: WHO; 2021 [cited 2022 28 August]. Available from: https://www.who.int/data/gho/data/themes/topics/anaemia_in_women_and_children accessed 28 August 2022.
4. National Statistical Office (NSO) [Malawi] and ICF. Malawi Demographic and Health Survey 2015-16,. Zomba, Malawi and Rockville, Maryland, USA: NSO and ICF, 2017.
5. Institute for Health Metrics and Evaluation (IHME). Global Burden of Disease (GBD) 2022 [cited 2022. Available from: https://www.healthdata.org/gbd/2019 accessed 28 August 2022.
6. World Health Organization. Use of multiple micronutrient powders for point-of-use fortification of foods consumed by infants and young children aged 6-23 months and children aged 2-12 years. Geneva, Switzerland: WHO, 2016a.
7. World Health Organization. Guideline: Daily iron supplementation in infants and children. Geneva, Switzerland: WHO, 2016b.
8. World Health Organization, United Nations Children's Fund, United Nations University. Iron Deficiency Anaemia: Assessment, Prevention, and Control. A guide for programme managers. Geneva, Switzerland: WHO, 2001.
9. UNICEF. Multiple Micronutrient Powder: Supply and market update - July 2021. New York: UNICEF, 2021.
10. Pasricha SR, Hasan MI, Braat S, et al. Benefits and Risks of Iron Interventions in Infants in Rural Bangladesh. *N Engl J Med* 2021;385(11):982-95. doi: 10.1056/NEJMoa2034187 [published Online First: 2021/09/09]
11. Pasricha SR, Hayes E, Kalumba K, et al. Effect of daily iron supplementation on health in children aged 4-23 months: a systematic review and meta-analysis of randomised controlled trials. *Lancet Glob Health* 2013;1(2):e77-e86. doi: 10.1016/s2214-109x(13)70046-9 [published Online First: 2014/08/12]
12. De-Regil LM, Suchdev PS, Vist GE, et al. Home fortification of foods with multiple micronutrient powders for health and nutrition in children under two years of age. *Cochrane Database Syst Rev* 2011(9):Cd008959. doi: 10.1002/14651858.CD008959.pub2 [published Online First: 2011/09/09]
13. Akman M, Cebeci D, Okur V, et al. The effects of iron deficiency on infants' developmental test performance. *Acta Paediatr* 2004;93(10):1391-6. [published Online First: 2004/10/27]
14. Idjradinata P, Pollitt E. Reversal of developmental delays in iron-deficient anaemic infants treated with iron. *Lancet* 1993;341(8836):1-4. doi: 10.1016/0140-6736(93)92477-b [published Online First: 1993/01/02]

For peer review only

For peer review only - http://bmjopen.bmj.com/site/about/guidelines.xhtml

15. Adu-Afarwuah S, Lartey A, Brown KH, et al. Home fortification of complementary foods with micronutrient supplements is well accepted and has positive effects on infant iron status in Ghana. *Am J Clin Nutr* 2008;87(4):929-38. doi: 10.1093/ajcn/87.4.929 [published Online First: 2008/04/11]

16. Somassè YE, Dramaix M, Traoré B, et al. The WHO recommendation of home fortification of foods with multiple-micronutrient powders in children under 2 years of age and its effectiveness on anaemia and weight: a pragmatic cluster-randomized controlled trial. *Public Health Nutr* 2018;21(7):1350-58. doi: 10.1017/s1368980017003858 [published Online First: 2018/01/22]

17. Sazawal S, Black RE, Ramsan M, et al. Effects of routine prophylactic supplementation with iron and folic acid on admission to hospital and mortality in preschool children in a high malaria transmission setting: community-based, randomised, placebo-controlled trial. *Lancet* 2006;367(9505):133-43. doi: 10.1016/s0140-6736(06)67962-2 [published Online First: 2006/01/18]

18. Veenemans J, Milligan P, Prentice AM, et al. Effect of supplementation with zinc and other micronutrients on malaria in Tanzanian children: a randomised trial. *PLoS Med* 2011;8(11):e1001125. doi: 10.1371/journal.pmed.1001125 [published Online First: 2011/12/02]

19. Neuberger A, Okebe J, Yahav D, et al. Oral iron supplements for children in malaria-endemic areas. *Cochrane Database Syst Rev* 2016;2(2):Cd006589. doi: 10.1002/14651858.CD006589.pub4 [published Online First: 2016/02/28]

20. Jonker FA, Calis JC, van Hensbroek MB, et al. Iron status predicts malaria risk in Malawian preschool children. *PLoS One* 2012;7(8):e42670. doi: 10.1371/journal.pone.0042670 [published Online First: 2012/08/24]

21. Clark MA, Goheen MM, Fulford A, et al. Host iron status and iron supplementation mediate susceptibility to erythrocytic stage Plasmodium falciparum. *Nat Commun* 2014;5:4446. doi: 10.1038/ncomms5446 [published Online First: 2014/07/26]

22. Goheen MM, Wegmüller R, Bah A, et al. Anemia Offers Stronger Protection Than Sickle Cell Trait Against the Erythrocytic Stage of Falciparum Malaria and This Protection Is Reversed by Iron Supplementation. *EBioMedicine* 2016;14:123-30. doi: 10.1016/j.ebiom.2016.11.011 [published Online First: 2016/11/18]

23. Calis JC, Phiri KS, Faragher EB, et al. Severe anemia in Malawian children. *N Engl J Med* 2008;358(9):888-99. doi: 10.1056/NEJMoa072727 [published Online First: 2008/02/29]

24. Newton CR, Warn PA, Winstanley PA, et al. Severe anaemia in children living in a malaria endemic area of Kenya. *Trop Med Int Health* 1997;2(2):165-78. doi: 10.1046/j.1365-3156.1997.d01-238.x [published Online First: 1997/02/01]

25. Njua-Yafi C, Achidi EA, Anchang-Kimbi JK, et al. Malaria, helminths, co-infection and anaemia in a cohort of children from Mutengene, south western Cameroon. *Malar J* 2016;15:69. doi: 10.1186/s12936-016-1111-2 [published Online First: 2016/02/08]

26. Pasricha SR, Atkinson SH, Armitage AE, et al. Expression of the iron hormone hepcidin distinguishes different types of anemia in African children. *Sci Transl Med* 2014;6(235):235re3. doi: 10.1126/scitranslmed.3008249 [published Online First: 2014/05/09]

27. Zlotkin S, Newton S, Aimone AM, et al. Effect of iron fortification on malaria incidence in infants and young children in Ghana: a randomized trial. *Jama* 2013;310(9):938-47. doi: 10.1001/jama.2013.277129 [published Online First: 2013/09/05]

For peer review only - http://bmjopen.bmj.com/site/about/guidelines.xhtml

28. Pasricha SR, Gheorghe A, Sakr-Ashour F, et al. Net benefit and cost-effectiveness of universal iron-containing multiple micronutrient powders for young children in 78 countries: a microsimulation study. *Lancet Glob Health* 2020;8(8):e1071-e80. doi: 10.1016/s2214-109x(20)30240-0 [published Online First: 2020/07/28]

29. Fernando SD, Rodrigo C, Rajapakse S. The 'hidden' burden of malaria: cognitive impairment following infection. *Malar J* 2010;9:366. doi: 10.1186/1475-2875-9-366 [published Online First: 2010/12/22]

30. Clarke SE, Jukes MC, Njagi JK, et al. Effect of intermittent preventive treatment of malaria on health and education in schoolchildren: a cluster-randomised, double-blind, placebo-controlled trial. *Lancet (London, England)* 2008;372(9633):127-38. doi: 10.1016/s0140-6736(08)61034-x [published Online First: 2008/07/16]

31. Jukes MCH, Pinder M, Grigorenko EL, et al. Long-Term Impact of Malaria Chemoprophylaxis on Cognitive Abilities and Educational Attainment: Follow-Up of a Controlled Trial. *PLOS Clinical Trials* 2006;1(4):e19. doi: 10.1371/journal.pctr.0010019

32. Meremikwu MM, Donegan S, Sinclair D, et al. Intermittent preventive treatment for malaria in children living in areas with seasonal transmission. *The Cochrane database of systematic reviews* 2012(2):Cd003756. doi: 10.1002/14651858.CD003756.pub4 [published Online First: 2012/02/18]

33. WHO. Guidelines for the treatment of Malaria Third edition ed: World Health Organisation 2015.

34. Bigira V, Kapisi J, Clark TD, et al. Protective Efficacy and Safety of Three Antimalarial Regimens for the Prevention of Malaria in Young Ugandan Children: A Randomized Controlled Trial. *PLoS medicine* 2014;11(8):e1001689. doi: 10.1371/journal.pmed.1001689

35. World malaria report 2017: World Health Organisation, 2017.

36. Zgambo M, Mbakaya BC, Kalembo FW. Prevalence and factors associated with malaria parasitaemia in children under the age of five years in Malawi: A comparison study of the 2012 and 2014 Malaria Indicator Surveys (MISs). *PloS one* 2017;12(4):e0175537. doi: 10.1371/journal.pone.0175537 [published Online First: 2017/04/12]

37. WHO. Malawi: WHO statistical profile: World Health Organisation 2015.

38. Whitson D. Farta Child Survival Project: Final Evaluation: CARE Ethiopia, 2007.

39. Bayley N. Bayley Scales of Infant and Toddler Development, Third Edition (Bayley--III®) [Database record]. APA PsycTests., 2005.

40. National Statistical Office (NSO) and ICF Macro. Malawi Demographic and Health Survey 2010. Zomba, Malawi, and Calverton, Maryland, USA: National Statistical Office (NSO) and ICF Macro 2011.

41. National Malaria Control Programme (NMCP) [Malawi] and ICF International. Malawi Malaria Indicator Survey (MIS) 2014. Lilongwe, Malawi, and Rockville, Maryland, USA: NMCP and ICF International 2014.

42. WHO Multicentre Growth Reference Study Group. Assessment of differences in linear growth among populations in the WHO Multicentre Growth Reference Study. *Acta Paediatr Suppl* 2006;450:56-65. doi: 10.1111/j.1651-2227.2006.tb02376.x [published Online First: 2006/07/05]

For peer review only

For peer review only - http://bmjopen.bmj.com/site/about/guidelines.xhtml

43. Hamadani JD, Tofail F, Hilaly A, et al. Use of family care indicators and their relationship with child development in Bangladesh. *J Health Popul Nutr* 2010;28(1):23-33. doi: 10.3329/jhpn.v28i1.4520 [published Online First: 2010/03/11]

44. Radloff LS. The CES-D Scale: A Self-Report Depression Scale for Research in the General Population. . *Applied Psychological Measurement* 1977;1(3):385–401. doi: https://doi.org/10.1177/014662167700100306

45. Coates Jennifer, Anne Swindale, Paula Bilinsky. Household Food Insecurity Access Scale (HFIAS)for Measurement of Household Food Access: Indicator Guide (v. 3). Washington, D.C.: FHI 360/FANTA, 2007.

46. Swindale Anne, Paula Bilinsky. Household Dietary Diversity Score (HDDS) for Measurement of Household Food Access: Indicator Guide (v.2). Washington, D.C.: FHI 360/FANTA, 2006.

47. McCoy DC, Sudfeld CR, Bellinger DC, et al. Development and validation of an early childhood development scale for use in low-resourced settings. *Popul Health Metr* 2017;15(1):3. doi: 10.1186/s12963-017-0122-8 [published Online First: 2017/02/12]

48. Hamadani JD, Tofail F, Huda SN, et al. Cognitive deficit and poverty in the first 5 years of childhood in Bangladesh. *Pediatrics* 2014;134(4):e1001-8. doi: 10.1542/peds.2014-0694 [published Online First: 2014/10/01]

49. WHO. Guideline: Daily iron supplementation in infants and children. Geneva: World Health Organization, 2016.

50. Muriuki JM, Mentzer AJ, Webb EL, et al. Estimating the burden of iron deficiency among African children. *BMC Med* 2020;18(1):31. doi: 10.1186/s12916-020-1502-7 [published Online First: 2020/02/28]

51. Greenwood B, Cairns M, Chaponda M, Chico RM, Dicko A, Ouedraogo JB, Phiri KS, Ter Kuile FO, Chandramohan D. Combining malaria vaccination with chemoprevention: a promising new approach to malaria control. Malar J. 2021 Sep 6;20(1):361. doi: 10.1186/s12936-021-03888-8. PMID: 34488784; PMCID: PMC8419817.

For peer review only

For peer review only - http://bmjopen.bmj.com/site/about/guidelines.xhtml

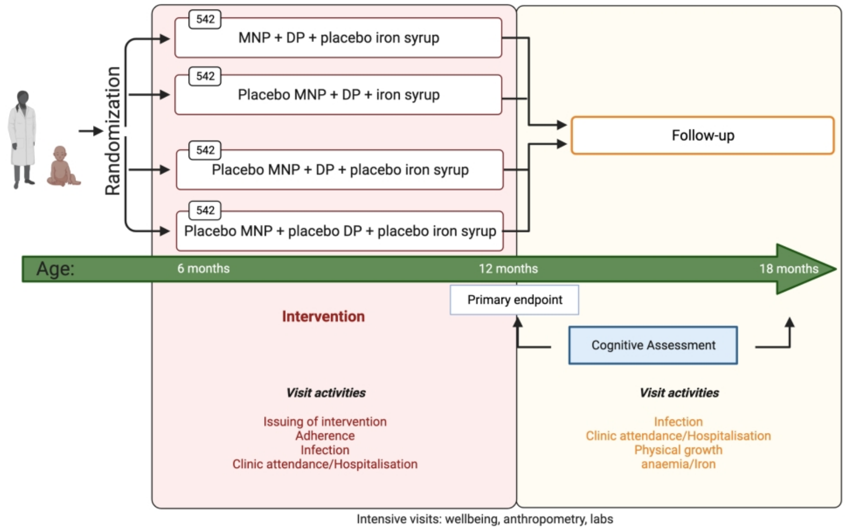

Figure 1: IRMA study design

645x516mm (118 x 118 DPI)

Supplementary material 1: Multiple micronutrient powders (MNPs) contents

	Micronutrient name	Quantity
1.	Niacinamide	7.02 - 8.58 mg/g
2.	Riboflavin	0.653 - 0.798 mg/g
3.	Pyridoxine	0.585 – 0.715 mg/g
4.	Thiamine	0.675 – 0.825 mg/g
5.	Vitamin C	32.4 – 39.6 mg/g
6.	Vitamin A	522 – 667 μgRE/g
7.	Vitamin E (as Tocopherol Eq.)	5.04 – 6.16 mgTE/g
8.	Vitamin D3	6.75 – 8.63 μgD/g
9.	Vitamin B12	1.09 – 1.40 μg/g
10.	Folic Acid	109 – 134 μg/g
11.	Selenium	17.0 – 24.5 μg/g
12.	Iodine	9.00 -129.6 μg/g
13.	Iron	10.00 -12.10 mg/g
14.	Zinc	4.10 – 0.739 mg/g
15.	Copper	0.605 – 0.739 mg/g

For peer review only

Supplementary material 2: Main study activities per visit

		Intervention period (Active follow-up)													Post intervention period (Passive follow up)					
	**Month**	**M1**			**M2**		**M3**		**M4**		**M5**		**M6**		**M7**	**M8**	**M9**	**M10**	**M11**	**M12**[‡]
**Planned activity**	**Day** 0	D 1[#]	D 14	D 28	D 42	D 56	D 70	D 84	D 98	D 112	D 126	D 140	D 154	D 168[§]						
**Location**	**H/HF**	**HF**	**H**	**H**	**H**	**H**	**H**	**H**	**H**	**H**	**H**	**H**	**H**	**HF**	**H**	**H**	**H**	**H**	**H**	**HF**
Pre-screening/ screening, & informed consent	**X**	**X**																		
Randomization		**X**																		
Demographic, clinical, CREDI and SES assessments		**X**																		
Family Care Index (FCI)		**X**												**X**						**X**
Bayley-III Cognitive Composite Score (CogCS)														**X**						**X**
Bayley-III Motor (MotCS) and Language (LangCS) Composite Scores														**X**						**X**
Behaviour: Wolke's Behaviour Ratings (WBR)														**X**						**X**
Food security & dietary diversity questionnaires		**X**												**X**						**X**
Anthropometry[1] - (mother and child)		**X**												**X**						**X**
**Laboratory procedures**																				
Haemoglobin		**X**												**X**						**X**
Malaria microscopy		**X**												**X**						**X**

For peer review only

Serum iron marker tests[2]		X												X						X
Serum for inflammatory marker tests (CRP/AGP)[3]		X												X						X
**Active follow-up:**																				
Compliance assessment		X	X	X	X	X	X	X	X	X	X	X	X	X						
MNPs/Placebo MNPs[4](distribute, instruct)		X		X		X		X		X		X								
DP/Placebo DP[5] (distribute, instruct & observe use/intake)		X		X		X		X		X		X								
Iron syrup/Placebo iron syrup[6]		X		X		X		X		X		X								
Provide educational information		X	X	X	X	X	X	X	X	X	X	X	X	X						
Assess infections[7]			X	X	X	X	X	X	X	X	X	X	X	X	X	X	X	X	X	X
Adverse Events monitoring			X	X	X	X	X	X	X	X	X	X	X	X	X	X	X	X	X	X
Health care utilization monitoring		X	X	X	X	X	X	X	X	X	X	X	X	X	X	X	X	X	X	X

# - Baseline; § - Midline visit at day 168. Primary outcome was assessed at day 168 ± 14 days as per protocol)**; ‡ - Endline; M –** Month; **D –** Day; **H – Home** (visit at the Participant's Home)**; HF –** Visit at the Health Facility; **X** – scheduled activity. **CREDI -** Caregiver Reported Early Development Instrument**; SES -** Social Economic Status; [1]**Anthropometric assessments** (mother and child): height/length, weight; [2]**Serum iron marker tests**: Serum ferritin; [3]**Inflammation markers**: CRP (C-Reactive Protein) or AGP (alpha 1 acid glycoprotein); [4]**MNPs** containing 10.0mg iron and 14 other micronutrients, daily for 6 months; **Placebo MNPs**: (maltodextrin), daily for 6 months; [5]**DP/Placebo DP** (20mg dihydroartemisinin/ 160mg piperaquine in suspension) dosed by body weight, three consecutive days every 4 weeks for 6 months; [6]**Iron syrup/Placebo iron syrup:** 10mg daily for six months; [7]**Infections**(assessment): (rate and number of days affected) fever/respiratory/diarrhoea.

NB: Families were encouraged to permit collection of venous blood for venous haemoglobin and iron and inflammation biomarkers at all timepoints; however, if a family refused venous blood collection or if collection was unsuccessful, data for the primary outcome and other non-laboratory secondary outcomes was still collected.

Reporting checklist for protocol of a clinical trial.

Based on the SPIRIT guidelines.

Instructions to authors

Complete this checklist by entering the page numbers from your manuscript where readers will find each of the items listed below.

Your article may not currently address all the items on the checklist. Please modify your text to include the missing information. If you are certain that an item does not apply, please write "n/a" and provide a short explanation.

Upload your completed checklist as an extra file when you submit to a journal.

In your methods section, say that you used the SPIRITreporting guidelines, and cite them as:

Chan A-W, Tetzlaff JM, Gøtzsche PC, Altman DG, Mann H, Berlin J, Dickersin K, Hróbjartsson A, Schulz KF, Parulekar WR, Krleža-Jerić K, Laupacis A, Moher D. SPIRIT 2013 Explanation and Elaboration: Guidance for protocols of clinical trials. BMJ. 2013;346:e7586

		Reporting Item	Page Number
**Administrative information**			
Title	#1	Descriptive title identifying the study design, population, interventions, and, if applicable, trial acronym	1
Trial registration	#2a	Trial identifier and registry name. If not yet registered, name of intended registry	4
Trial registration: data set	#2b	All items from the World Health Organization Trial Registration Data Set	4
Protocol version	#3	Date and version identifier	31
Funding	#4	Sources and types of financial, material, and other support	31
Roles and responsibilities: contributorship	#5a	Names, affiliations, and roles of protocol contributors	30

For peer review only

Roles and responsibilities: sponsor contact information	#5b	Name and contact information for the trial sponsor	31
Roles and responsibilities: sponsor and funder	#5c	Role of study sponsor and funders, if any, in study design; collection, management, analysis, and interpretation of data; writing of the report; and the decision to submit the report for publication, including whether they will have ultimate authority over any of these activities	31-32
Roles and responsibilities: committees	#5d	Composition, roles, and responsibilities of the coordinating centre, steering committee, endpoint adjudication committee, data management team, and other individuals or groups overseeing the trial, if applicable (see Item 21a for data monitoring committee)	22-23
**Introduction**			
Background and rationale	#6a	Description of research question and justification for undertaking the trial, including summary of relevant studies (published and unpublished) examining benefits and harms for each intervention	6-10
Background and rationale: choice of comparators	#6b	Explanation for choice of comparators	14
Objectives	#7	Specific objectives or hypotheses	10-12
Trial design	#8	Description of trial design including type of trial (eg, parallel group, crossover, factorial, single group), allocation ratio, and framework (eg, superiority, equivalence, non-inferiority, exploratory)	12
**Methods: Participants, interventions, and outcomes**			
Study setting	#9	Description of study settings (eg, community clinic, academic hospital) and list of countries where data will be collected. Reference to where list of study sites can be obtained	12
Eligibility criteria	#10	Inclusion and exclusion criteria for participants. If applicable, eligibility criteria for study centres and individuals who will	12-13

For peer review only

		perform the interventions (eg, surgeons, psychotherapists)	
Interventions: description	#11a	Interventions for each group with sufficient detail to allow replication, including how and when they will be administered	14
Interventions: modifications	#11b	Criteria for discontinuing or modifying allocated interventions for a given trial participant (eg, drug dose change in response to harms, participant request, or improving / worsening disease)	14
Interventions: adherance	#11c	Strategies to improve adherence to intervention protocols, and any procedures for monitoring adherence (eg, drug tablet return; laboratory tests)	20
Interventions: concomitant care	#11d	Relevant concomitant care and interventions that are permitted or prohibited during the trial	12-13
Outcomes	#12	Primary, secondary, and other outcomes, including the specific measurement variable (eg, systolic blood pressure), analysis metric (eg, change from baseline, final value, time to event), method of aggregation (eg, median, proportion), and time point for each outcome. Explanation of the clinical relevance of chosen efficacy and harm outcomes is strongly recommended	23
Participant timeline	#13	Time schedule of enrolment, interventions (including any run-ins and washouts), assessments, and visits for participants. A schematic diagram is highly recommended (see Figure)	15-22
Sample size	#14	Estimated number of participants needed to achieve study objectives and how it was determined, including clinical and statistical assumptions supporting any sample size calculations	24
Recruitment	#15	Strategies for achieving adequate participant enrolment to reach target sample size	15
**Methods: Assignment of interventions (for controlled trials)**			
Allocation: sequence generation	#16a	Method of generating the allocation sequence (eg, computer-generated random numbers), and list of any factors for stratification. To reduce predictability of a random sequence, details of any planned restriction (eg, blocking) should be provided in a separate document that is unavailable to those who enrol participants or assign interventions	14-15

For peer review only

Allocation concealment mechanism	#16b	Mechanism of implementing the allocation sequence (eg, central telephone; sequentially numbered, opaque, sealed envelopes), describing any steps to conceal the sequence until interventions are assigned	14-15
Allocation: implementation	#16c	Who will generate the allocation sequence, who will enrol participants, and who will assign participants to interventions	14-15
Blinding (masking)	#17a	Who will be blinded after assignment to interventions (eg, trial participants, care providers, outcome assessors, data analysts), and how	14-15
Blinding (masking): emergency unblinding	#17b	If blinded, circumstances under which unblinding is permissible, and procedure for revealing a participant's allocated intervention during the trial	14-15
**Methods: Data collection, management, and analysis**			
Data collection plan	#18a	Plans for assessment and collection of outcome, baseline, and other trial data, including any related processes to promote data quality (eg, duplicate measurements, training of assessors) and a description of study instruments (eg, questionnaires, laboratory tests) along with their reliability and validity, if known. Reference to where data collection forms can be found, if not in the protocol	15-22
Data collection plan: retention	#18b	Plans to promote participant retention and complete follow-up, including list of any outcome data to be collected for participants who discontinue or deviate from intervention protocols	15-22
Data management	#19	Plans for data entry, coding, security, and storage, including any related processes to promote data quality (eg, double data entry; range checks for data values). Reference to where details of data management procedures can be found, if not in the protocol	24-25
Statistics: outcomes	#20a	Statistical methods for analysing primary and secondary outcomes. Reference to where other details of the statistical analysis plan can be found, if not in the protocol	24
Statistics: additional analyses	#20b	Methods for any additional analyses (eg, subgroup and adjusted analyses)	24

For peer review only

Statistics: analysis population and missing data	#20c	Definition of analysis population relating to protocol non-adherence (eg, as randomised analysis), and any statistical methods to handle missing data (eg, multiple imputation)	24
**Methods: Monitoring**			
Data monitoring: formal committee	#21a	Composition of data monitoring committee (DMC); summary of its role and reporting structure; statement of whether it is independent from the sponsor and competing interests; and reference to where further details about its charter can be found, if not in the protocol. Alternatively, an explanation of why a DMC is not needed	22-23
Data monitoring: interim analysis	#21b	Description of any interim analyses and stopping guidelines, including who will have access to these interim results and make the final decision to terminate the trial	25
Harms	#22	Plans for collecting, assessing, reporting, and managing solicited and spontaneously reported adverse events and other unintended effects of trial interventions or trial conduct	23-24
Auditing	#23	Frequency and procedures for auditing trial conduct, if any, and whether the process will be independent from investigators and the sponsor	25
**Ethics and dissemination**			
Research ethics approval	#24	Plans for seeking research ethics committee / institutional review board (REC / IRB) approval	26
Protocol amendments	#25	Plans for communicating important protocol modifications (eg, changes to eligibility criteria, outcomes, analyses) to relevant parties (eg, investigators, REC / IRBs, trial participants, trial registries, journals, regulators)	26
Consent or assent	#26a	Who will obtain informed consent or assent from potential trial participants or authorised surrogates, and how (see Item 32)	19
Consent or assent: ancillary studies	#26b	Additional consent provisions for collection and use of participant data and biological specimens in ancillary studies, if applicable	26
Confidentiality	#27	How personal information about potential and enrolled participants will be collected, shared, and maintained in order to protect confidentiality before, during, and after the trial	26

For peer review only

Declaration of interests	#28	Financial and other competing interests for principal investigators for the overall trial and each study site	30
Data access	#29	Statement of who will have access to the final trial dataset, and disclosure of contractual agreements that limit such access for investigators	25
Ancillary and post trial care	#30	Provisions, if any, for ancillary and post-trial care, and for compensation to those who suffer harm from trial participation	
Dissemination policy: trial results	#31a	Plans for investigators and sponsor to communicate trial results to participants, healthcare professionals, the public, and other relevant groups (eg, via publication, reporting in results databases, or other data sharing arrangements), including any publication restrictions	22
Dissemination policy: authorship	#31b	Authorship eligibility guidelines and any intended use of professional writers	30
Dissemination policy: reproducible research	#31c	Plans, if any, for granting public access to the full protocol, participant-level dataset, and statistical code	31
**Appendices**			
Informed consent materials	#32	Model consent form and other related documentation given to participants and authorised surrogates	26
Biological specimens	#33	Plans for collection, laboratory evaluation, and storage of biological specimens for genetic or molecular analysis in the current trial and for future use in ancillary studies, if applicable	22

None The SPIRIT Explanation and Elaboration paper is distributed under the terms of the Creative Commons Attribution License CC-BY-NC. This checklist can be completed online using https://www.goodreports.org/, a tool made by the EQUATOR Network in collaboration with Penelope.ai

For peer review only

BMJ Open

Protocol for a randomised, multicentre, four-arm, double-blinded, placebo-controlled trial to assess the benefits and safety of Iron supplementation with Malaria chemoprevention to children in Malawi - IRMA trial

Journal:	*BMJ Open*
Manuscript ID	bmjopen-2022-069011.R2
Article Type:	Protocol
Date Submitted by the Author:	22-Jun-2023
Complete List of Authors:	Mwangi, Martin; Training and Research Unit of Excellence (TRUE), Nutrition and Infectious Diseases ; The Micronutrient Forum, Healthy Mothers Healthy Babies Consortium<br>Mzembe, Glory; Training and Research Unit of Excellence (TRUE), Nutrition and Infectious Diseases ; Kamuzu University of Health Sciences, Public Health<br>Ngwira, Chikondi ; Training and Research Unit of Excellence (TRUE), Nutrition and Infectious Diseases ; Liverpool School of Tropical Medicine<br>Vokhiwa, Maclean; Training and Research Unit of Excellence (TRUE), Nutrition and Infectious Diseases<br>Kapulula, Mayamiko; Training and Research Unit of Excellence (TRUE), Nutrition and Infectious Diseases<br>Larson, Leila; University of South Carolina Arnold School of Public Health, Department of Health Promotion, Education, and Behaviour<br>Braat, Sabine ; University of Melbourne School of Population and Global Health, Centre for Epidemiology and Biostatistics; Walter and Eliza Hall Institute of Medical Research<br>Harding, Rebecca; The Walter and Eliza Hall Institute of Medical Research,<br>McLean, Alistair; Walter and Eliza Hall Institute of Medical Research; The University of Melbourne, Department of Infectious Diseases<br>Hamadani, Jena; International Centre for Diarrhoeal Disease Research<br>Biggs, Beverley-Ann ; The University of Melbourne, Medicine<br>Ataíde, Ricardo; The Walter and Eliza Hall Institute of Medical Research; The University of Melbourne, Department of Infectious Diseases at the Peter Doherty Institute of Infection and Immunity<br>Phiri, Kamija; Training and Research Unit of Excellence (TRUE), Nutrition and Infectious Diseases ; Kamuzu University of Health Sciences, Public Health<br>Pasricha, Sant-Rayn; Walter and Eliza Hall Institute of Medical Research,
**Primary Subject Heading**:	Public health
Secondary Subject Heading:	Haematology (incl blood transfusion)
Keywords:	Anaemia < HAEMATOLOGY, Infection control < INFECTIOUS DISEASES, PUBLIC HEALTH, Nutrition < TROPICAL MEDICINE

Protocol for a randomised, multicentre, four-arm, double-blinded, placebo-controlled trial to assess the benefits and safety of Iron supplementation with Malaria chemoprevention to children in Malawi - IRMA trial

Authors

*Martin N. Mwangi[1,2], *Glory Mzembe[1,3], Chikondi C. Ngwira[1], Maclean Vokhiwa[1,3], Mayamiko D. Kapulula[1], Leila M. Larson[4], Sabine Braat[5,6,7], Rebecca Harding[7], Alistair R.D. McLean[6,7], Jena D. Hamadani[8], Beverley-Ann Biggs[5,9], Ricardo Ataíde[5,7], Kamija S. Phiri[1,3], Sant-Rayn Pasricha [7,9,10]

*Joint first authors

Corresponding author:

Martin N. Mwangi, Training and Research Unit of Excellence (TRUE), 1 Kufa Road, Post Office Box 30538, Blantyre, Malawi. Email: mmwangi@true.mw

Author Institutions

1. Training and Research Unit of Excellence (TRUE), Blantyre, Malawi.
2. The Micronutrient Forum, Healthy Mothers Healthy Babies Consortium, Washington DC, USA.
3. Department of Public Health, School of Public and Global Health, Kamuzu University of Health Sciences, Blantyre, Malawi.
4. Department of Health Promotion, Education, and Behaviour, Arnold School of Public Health, University of South Carolina, Columbia, South Carolina, USA.
5. Department of Infectious Diseases, University of Melbourne, at the Peter Doherty Institute for Infection and Immunity, Melbourne, Australia.

For peer review only - http://bmjopen.bmj.com/site/about/guidelines.xhtml

6. Centre for Epidemiology and Biostatistics, Melbourne School of Population and Global Health, The University of Melbourne, Melbourne, Australia.

7. Population Health and Immunity Division, The Walter and Eliza Hall Institute of Medical Research, Melbourne, Australia.

8. International Centre for Diarrhoeal Disease Research, Bangladesh, Dhaka, Bangladesh.

9. DDiagnostic Haematology, The Royal Melbourne Hospital; and Clinical Haematology, Melbourne, Australia.

10. Department of Medical Biology, Faculty of Medicine, Dentistry and Health Sciences, The University of Melbourne, Melbourne, Australia.

Word count: 4750. (Abstract) 298 words. *(excluding title page, abstract, references, figures and tables)*

For peer review only

For peer review only - http://bmjopen.bmj.com/site/about/guidelines.xhtml

ABSTRACT

Introduction: Approximately 40% of children 6-59 months of age worldwide are anaemic. Iron-containing multiple micronutrient powders (MNPs) and iron supplements (syrup/drops) are used to combat anaemia in children in different parts of the world. However, evidence for functional benefits of iron supplementation in children is scarce, and potential risks remain poorly defined, particularly concerning diarrhoea and malaria. The trial aims to determine if: a) the efficacy of iron supplements or MNPs (containing iron) given with malaria chemoprevention is superior to malaria chemoprevention alone, or b) if the efficacy of malaria chemoprevention alone is superior to placebo on child cognitive development.

Methods and Analysis: IRMA is a four-arm, parallel-group, double-blinded, placebo-controlled, triple-dummy, randomised trial in Southern Malawi. The study recruits 2,168 infants aged six months, with an intervention period of six months and a post-intervention period of a further six months. Children are randomised into four arms: (a) No intervention (placebo); (b) malaria chemoprevention only; (c) MNPs and Malaria chemoprevention; and (d) Iron syrup and malaria chemoprevention. The primary outcome, cognitive development (CogCS), is measured at the end of the six months intervention. Secondary outcomes include CogCS at a further six months post-intervention, motor, language and behavioural development, physical growth, and prevalence of anaemia and iron deficiency. Safety outcomes include incidence of malaria and other infections, and prevalence of malaria parasitaemia during and post-intervention period.

Ethics and dissemination: The trial is approved by the National Health Sciences Research Committee (#19/01/2213) (Malawi) and the Human Research Ethics Committee (WEHI: 19/012) (Australia). Written informed consent in the local language is obtained from each

For peer review only - http://bmjopen.bmj.com/site/about/guidelines.xhtml

participant before conducting any study-related procedure. Results will be shared with the local community and internationally with academic and policy stakeholders.

Protocol registration details: The protocol is registered with the Australian and New Zealand Clinical Trials Registry (ACTRN12620000386932).

Keywords: Anaemia, Infant, Infection, Iron Deficiency, Malawi, Malaria, Micronutrient Powders, randomised controlled trial.

Strengths and limitations of this study

- The trial setting represents malaria-endemic Sub-Saharan African countries where anaemia and malaria co-exist, which is an ideal place to study the benefits versus risks of iron supplementation (under malaria chemoprevention) to establish a generalisable clinical rationale.
- The participant screening and inclusion criteria involve all infants within the recruitment age range across a large geographical catchment area which enables the generalizability of the study results to most national programs in malaria-endemic countries.
- The trial compares the two major delivery modes for iron in young children: MNPs and iron syrup which will help identify the ideal and most effective iron source for children.
- The trial utilises a functional clinical health outcome – child cognitive development – as its primary outcome, ensuring the trial provides evidence of the functional relevance of universal iron interventions and malaria prevention.
- The trial is powered to detect a small but clinically relevant effect for the primary outcome of child cognitive development; the use of a post-intervention follow-up

For peer review only

For peer review only - http://bmjopen.bmj.com/site/about/guidelines.xhtml

period enables the assessment of both the immediate and medium-term impact of the intervention.

INTRODUCTION

Anaemia remains a critical global health problem affecting up to 39.8% of children aged 6 – 59 months worldwide and 60.2% in Africa.[1-3] In Malawi, 63% of children 6-59 months are anaemic, with a peak prevalence of 91% observed among children aged 9-11 months.[4] Iron deficiency accounts for most (60%) of the global burden of anaemia[5] but less so in malaria-endemic areas, where only 32% of childhood anaemia is iron responsive.[1]

World Health Organisation (WHO) anaemia prevention guidelines recommend two universal iron supplementation strategies for children, namely; 1) iron supplement drops or syrups (10-12.5mg of elemental iron) – given daily to children 6-24 months in areas where the prevalence of anaemia is 40% or higher;[6-8] and 2) iron-containing multiple micronutrient powders (MNPs): 10-12.5mg of iron – given daily over six months in areas where the prevalence of anaemia is 20% or higher.[6-8] However, no recommendation is given regarding the preferred approach. MNPs are the most common form of iron supplementation, with at least 58 countries reported to have implemented MNPs home fortification by 2021.[9] The rationale for universal iron supplementation is an understanding that iron supplementation would reduce childhood anaemia and improve child development and other functional health outcomes. However, we recently showed that in a rural South Asian population, neither universal distribution of oral iron supplements nor MNPs meaningfully improved immediate or medium-term child cognitive, language, motor, and other developmental outcomes, even though anaemia and iron deficiency were reduced.[10] However, these data were generated

For peer review only - http://bmjopen.bmj.com/site/about/guidelines.xhtml

from a trial in a non-malaria endemic part of South Asia, where the determinants of anaemia and infection risk are likely very different.

Benefits of universal iron supplementation programmes

Universal iron supplementation programs have shown benefits in haematological outcomes. A meta-analysis[11] and a Cochrane review[12] showed improvements in anaemia with daily iron supplementation in children. However, the effects on growth and development are less clear. Few studies have investigated the impact on cognitive development. Only 8 of the 33 studies included in the meta-analysis by Pasricha et al. assessed cognitive development, and no significant beneficial effect was seen (mean difference 1.65 [95% CI -0.63, 3.94]), but data were sparse.[11] Subgroup analysis did suggest a benefit of iron supplementation on cognitive development in iron-deficient children. However, this data came from only two studies.[13 14] Only one study in the Cochrane review assessed child development, showing that children receiving MNPs were more likely to walk independently at 12 months of age than those receiving no intervention.[15] There is also little evidence for a beneficial effect on growth;[11 12 16] however, data were limited.[11] WHO has acknowledged the need for further research into the functional outcomes of iron supplementation programs.[7]

For peer review only

Risks of iron supplementation

Whilst the evidence for functional benefits of iron supplementation is scarce, there has been emerging evidence of potential harm, particularly regarding increased malaria risk in endemic areas. The seminal Pemba RCT in Tanzania was stopped early due to increased death, hospitalisation and malaria in iron replete children receiving iron compared with placebo and called for revisions of universal iron supplementation recommendations.[17] In another RCT in Tanzania, multi-nutrient supplementation (including iron) increased the number of malaria

For peer review only - http://bmjopen.bmj.com/site/about/guidelines.xhtml

cases by 41% in iron-deficient children.[18] However, a 2016 Cochrane review found iron supplementation safe in areas with adequate malaria prevention and management strategies.[19] In contrast, there was an increased risk in areas where this was not the case.[19] The current recommendation is for universal iron supplementation in malaria-endemic regions with a high prevalence of anaemia only "in conjunction with public health measures to prevent, diagnose and treat malaria."[7]

Iron deficiency protects against malaria

There is evidence to suggest that iron deficiency is protective against malaria. A study of preschool children in Malawi found that iron-deficient children were at lower risk of clinical malaria and parasitaemia than iron-replete children.[20] *In vitro* studies have also demonstrated that *Plasmodium falciparum* parasites are less effective at invading iron deficient red cells and prefer invading reticulocytes (which are increased in the recovery phase of iron deficiency anaemia when iron supplementation is given).[21] Parasite invasion and growth were reduced in laboratory growth and invasion studies of *P. falciparum* using red cells from anaemic children.[22] The protective effect imparted by iron deficiency in both in vitro studies was reversed when iron supplementation was given.[21 22]

Anaemia in malaria-endemic areas is less iron-responsive

Malaria is a significant cause of severe anaemia, hospitalisation, and death in endemic areas.[23-25] Several aetiologies, including malaria, were identified in a study of Malawian children with severe anaemia. However, iron deficiency was not a common contributor.[23] Similarly, in a study in children from The Gambia and Tanzania,[26] 61.2% of children were anaemic, but only 13% had iron deficiency anaemia.[26] In keeping with this, the anaemia in these areas is less iron-responsive.[11 27] Without clear evidence of the neurodevelopmental

For peer review only - http://bmjopen.bmj.com/site/about/guidelines.xhtml

benefits of iron supplementation, iron interventions are likely to be extremely expensive public health interventions in terms of cost per DALY averted, especially in sub-Saharan Africa.[28]

Malaria may be a risk factor for impaired cognitive development

Despite few studies, the majority with relatively small sample sizes and varying methods of assessing cognitive function, evidence suggests that severe and uncomplicated malaria may be independent risk factors for cognitive impairment.[29] In addition, intermittent preventive therapy in preschool and school-aged children, has improved cognitive performance.[30 31]

Intermittent preventive treatment to decrease malaria and its sequelae

Intermittent Preventive Treatment in Children (IPTc) involves the empiric treatment of malaria at regular intervals and is a potential strategy to decrease malaria rates and sequelae. A Cochrane review that included seven RCTs evaluated the effect of chemoprophylaxis and intermittent treatment for malaria in preschool children in areas with seasonal malaria and showed a reduction in clinical malaria and moderate to severe anaemia.[32] Furthermore, in a meta-analysis, iron supplementation reduced the rate of anaemia by 30% in malaria-endemic areas, compared with 60% in non-endemic regions.[11]

DP as chemoprevention

Recommended treatment for *P. falciparum* malaria is with artemisinin-based combination therapy (ACT). DP is an ACT and is among WHO's five recommended first-line treatments.[33] DP with its long half-life, given monthly, is an attractive option for IPTc. In an RCT in children in Uganda using this regimen, DP reduced clinical malaria by 58% (compared with 28% for trimethoprim-sulfamethoxazole and 7% for sulfadoxine-pyrimethamine) and parasitaemia by

For peer review only - http://bmjopen.bmj.com/site/about/guidelines.xhtml

76%. IPTc with DP also reduced moderate-to-severe anaemia by 47%.[34] The administration of DP-IPTc during MNPs/iron supplementation may improve the safety of these interventions. However, there are concerns that malaria prevention may delay the development of immunity and increase malaria risk once IPTc is ceased.[32]

Iron deficiency and malaria co-exist in Malawi

Despite important gains made worldwide in malaria control since 2010, worldwide cases increased in 2016. In Malawi, there were a total of 5.2 million presumed and confirmed malaria cases in 2016, 231,970 more cases than the year prior, resulting from a gradual increase in cases since 2014.[35] In addition, the prevalence of malaria parasitaemia in children under five years increased from 28% in 2012 to 33% in 2014. These increases come despite increases in insecticide-treated bed net coverage.[36]

Both malaria and iron deficiency anaemia remain important causes of morbidity and mortality in Malawi. In Malawian children under five years of age, malaria is the leading cause of death,[37] and the prevalence of anaemia is 63%.[38] Addressing both of these conditions is challenging. Current WHO recommendations for improving anaemia in malaria-endemic regions are difficult to interpret and implement in countries like Malawi, where malaria preventive measures and treatment are present but by no means universal.

Why this trial is needed

Several fundamental questions concerning the benefits and safety of iron-intervention programs in malaria-endemic regions remain unanswered. The IRMA trial will assess functional health outcomes from iron supplementation and identify the ideal mode of iron

For peer review only - http://bmjopen.bmj.com/site/about/guidelines.xhtml

For peer review only

delivery (MNPs versus iron syrup/drops). It will also allow evaluation of the effect of malaria chemoprevention alone on anaemia and child development.

OBJECTIVES

The primary objective is to determine a) if the efficacy of iron supplements or MNPs (containing iron) given with malaria chemoprevention is superior to malaria chemoprevention alone, or b) if the efficacy of malaria chemoprevention alone is superior to placebo, on child cognitive development (measured using locally adapted Bayley Scales of Infant and Toddler Development 3rd Edition (Bayley-III) Cognitive Composite Scores[39]) at the end of the 6-month intervention period.

The specific objectives are:

1. Determine if the efficacy of a) iron (as supplements or MNPs) given with malaria chemoprevention is superior to malaria chemoprevention alone or b) if the efficacy of malaria chemoprevention alone is superior to placebo on;
   i. child cognitive development, child motor, language, behavioural development, temperament, and physical growth at the end of the six months intervention and a further six months post-intervention;
   ii. anaemia (and haemoglobin concentration), iron deficiency (and ferritin) and iron deficiency anaemia after six months of intervention and six months post intervention.
2. Compare the effects of iron given as supplements to MNPs on the above outcomes after six months of intervention and a further six-months post-intervention.

For peer review only

For peer review only - http://bmjopen.bmj.com/site/about/guidelines.xhtml

3. Determine the safety of iron (as supplements or MNPs) given with malaria chemoprevention on the risk of malaria and other morbidities versus malaria chemoprevention alone or placebo during and post-intervention.
4. Determine the efficacy of malaria chemoprevention alone compared to placebo on clinical malaria, parasitaemia and malaria-specific clinic visits, and all-cause sick visits during and after intervention.
5. Determine the safety of iron (as supplements or MNPs) given with malaria chemoprevention compared to placebo on infection risk in young children, during and post-intervention.

For peer review only

Study design

IRMA is a four-arm parallel-group, double-blinded, placebo-controlled, triple-dummy, individually randomised trial comparing: (a) No intervention, (b) Malaria chemoprevention only, (c) MNPs + Malaria chemoprevention and (d) Iron syrup + Malaria chemoprevention at the end of the six months intervention and at a further 6-months post-intervention. The trial design is summarised in **Figure 1**.

Study settings and participants

The trial is set in Chikwawa district, in Southern Malawi, a rural area where >70% of children 6-12 months are anaemic,[40] and where 33% of children under five years old have *Plasmodium falciparum* parasitaemia.[41] Four health facilities within the district are hosting the trial, namely: Chikwawa District Hospital, Mapelera Health Centre, Mfera Health Centre and Makhwira Health Centre. These are the primary health facilities where children attend routine under-five clinics and sick visits. Our target participants are all children aged six months residing in these health facilities' catchment area.

For peer review only - http://bmjopen.bmj.com/site/about/guidelines.xhtml

Eligibility criteria

The criteria to select participants considers participants' safety, the generalizability to national programs, and the feasibility of the trial. Children are randomly assigned to a study intervention group only if they meet all the inclusion and none of the exclusion criteria.

Inclusion criteria

1. Infant aged six months (+/- 14 days) at randomisation.
2. Availability of a legally acceptable guardian/representative and providing written consent on the participant's behalf.

Exclusion criteria

1. Severe anaemia (Hb<7g/dL).
2. Clinical signs of current infective illness (respiratory infection, diarrhoea, malaria); however, children will be screened after recovery and recruited if they meet the age eligibility criteria at the second screening.
3. Severe malnutrition (weight-for-length z-score<-3) or MUAC < 11.5 cm.
4. Established haemoglobinopathy diagnosis (e.g., sickle cell disease, beta thalassaemia major, HbE-beta thalassaemia).
5. Known congenital anomaly, developmental disorder, or severe developmental delay.
6. Children with physical or behavioural problems that will make it impossible to undertake trial assessments.
7. Children of multiple births, e.g., twins.
8. Children currently consuming iron supplements or MNPs, e.g., from the National MNPs programme.

For peer review only - http://bmjopen.bmj.com/site/about/guidelines.xhtml

9. Enrolment in another trial.

Trial Interventions

Participants are randomly assigned to receive one of the following interventions:

1) **No intervention (Placebo DP *plus* Placebo MNPs *plus* placebo iron syrup)**: Placebo DP (Guilin Pharmaceutical company, China)**:** three consecutive days every four weeks for six months *plus* Placebo MNPs (maltodextrin vehicle only; DSM Nutritional Products, South Africa) daily for six months *plus* placebo iron syrup (ACME Laboratories Ltd., Bangladesh) daily for six months.
2) **Malaria chemoprevention only (Active DP *plus* placebo MNPs *plus* placebo iron syrup)**: Active DP (20mg dihydroartemisinin/160mg piperaquine; Guilin Pharmaceutical company, China) dosed by body weight (2/16mg/Kg) for three consecutive days every four weeks for six months *plus* placebo MNPs daily for six months, *plus* daily placebo iron syrup for six months.
3) **MNP + malaria chemopevention (Active DP *plus* active MNPs *plus* placebo iron syrup):** Active DP as above, *plus* daily active MNPs containing 10.0mg iron and 14 other micronutrients (see supplementary material 1); DSM Nutritional Products, South Africa, *plus* placebo iron syrup daily for six months.
4) **Iron syrup + malaria chemoprevention (Active DP *plus* placebo MNPs *plus* active iron syrup):** Active DP as above, *plus* placebo MNPs, *plus* active iron syrup containing 10.0mg ferrous sulphate (ACME Laboratories Ltd., Bangladesh) daily for six months.

Randomisation, allocation concealment, and blinding

Participants are randomly allocated to one of the four study arms with 1:1:1:1 allocation via a computer-generated randomisation schedule of randomly permuted blocks stratified by sex

For peer review only - http://bmjopen.bmj.com/site/about/guidelines.xhtml

and health facility/study sites (Chikwawa District Hospital, Mapelera health centre, Mfera health centre and Makhwira Health Centre) to achieve a balance between the intervention arms. An independent statistician from the University of Melbourne (Australia) generated the randomisation list.

An independent Trial Pharmacist pre-packed and pre-labelled participant packs containing all three investigational products based on the randomisation list. Each participating child who meets the inclusion criteria is sequentially allocated to the next available study number indicated on the randomisation list. The participant is then given a pre-labelled pre-packed Ziplock bag containing the study medication corresponding to the study number assigned to them. Hence, participants and their families and all study team members involved in collecting and analysing outcome data, including field workers, developmental assessors, laboratory scientists, statisticians, and investigators, are blinded to the treatment allocation until the database is locked and before unblinding.

For peer review only

Recruitment and Visits

Patient and Public Involvement

All levels of the local leadership (head men/women, sub-chiefs, chiefs, Community Health Action Groups (CHAGS), Health Surveillance Assistants (HSAs) and District Health Assembly (DHA)) are continuously engaged by the trial's Community Engagement Team (CET). CHAGs and HSAs were engaged to ensure that we reach the whole community via it's resident health "gate-keepers". Additionally, the CET conducted community sensitisation meetings to explain the research objectives, what it means to participate, benefits for the individual or the community, and overall contribution to science. Finally, community members are regularly invited to contribute ideas that could help improve the success of the research thereby

For peer review only - http://bmjopen.bmj.com/site/about/guidelines.xhtml

increasing the probability of success.

Screening and enrolment of participants

Study recruitment opened on 17 April 2020. Study activities are as detailed below (also detailed in supplementary material 2).

Pre-screening

We conducted a house-to-house enumeration (census) of all potential participants (children under six months of age) living in the target area and generated a study population database. New births are identified and reported by HSAs or registered at the health facilities' under-five clinics throughout the trial recruitment period and added to the database. Research Assistants visit all children approaching six months of age in the community. After taking written informed consent, the team reviews the child's medical history to determine potential eligibility. Eligible children are invited to visit a selected health facility for further screening.

Screening

On a pre-determined day, the child, accompanied by their parent(s) or legal guardian, visit the selected health centre. HemoCue Hb301+ (Angelholm, Sweden) is used to assess capillary Hb level to exclude severely anaemic children. Additionally, anthropometric measurements: weight and length (used to calculate weight-for-length z-scores[42]) and mid-upper arm circumference (MUAC) are performed to exclude severely malnourished children. Severely anaemic and severely malnourished children are referred to the routine health system for further investigation and treatment.

Baseline and Enrolment (at six months of age (+/- 14 days))

For peer review only - http://bmjopen.bmj.com/site/about/guidelines.xhtml

A second, more detailed consent form for participation in the study is signed, and a study identification number is assigned. Randomisation is performed after the following baseline evaluations (see **supplementary material 2**) are carried out and necessary information collected: demographic information, socioeconomic status (using the Malawi Demographic Health Survey (MDHS) standard of living scale), medical history, family care indicators[43]/index (FCI), feelings questionnaire,[44] cost survey (household out of pocket health care costs), food security questionnaire,[45] dietary diversity questionnaire,[46] developmental screening using Caregiver Reported Early Development Instrument (CREDI),[47] anthropometric measurements (weight, length, MUAC and head circumference), and anthropometric measurements for the mother (weight, height). A venous blood sample (3 mL), for haemoglobin testing, determination of iron indices (serum – for ferritin, CRP, and AGP), thick and thin malaria slides for malaria microscopy testing (to be conducted at the completion of the trial) and filter paper for plasmodium PCR, is collected from the child. The mother is given educational information regarding the use of study medications, administration of the first dose of DP and instructions on how to administer day two and day three DP treatments, administration of the first dose of iron syrup, use of MNPs and advice on future visits.

Subsequent study visits

Adherence assessment

Fortnightly 'home visits' by experienced research assistants to the participants' homes in the community are done to assess the following: 1) documentation of episodes of infection (rate, days affected), 2) recording any adverse events/effects, 3) documentation of details of unplanned hospital and healthcare facility attendance for example to a doctor, traditional healer, clinic or hospital, 4) provision of educational information to participants on the correct

For peer review only

For peer review only - http://bmjopen.bmj.com/site/about/guidelines.xhtml

use of the study interventions and encouraging adherence to study interventions, 5) adherence monitoring (measured from parental questioning along with assessment of residual MNPs and syrup) and 6) advice on future visits.

Monthly DP issuance

The research assistants issue the investigational products monthly during a home visit. The following activities (including data collection) are undertaken: issuance of the study interventions according to study protocol and adherence monitoring (measured from parental questioning along with assessment of residual MNPs and syrup). In addition, unused investigational products are collected.

Study midline visit (at 12 months of age +/- 14 days)

The participating child and their parent/ legal guardian attend the health facility at the end of the six months intervention for developmental assessments using locally adapted Bayley Scales of Infant and Toddler Development, Wolke's Behavioural Rating (WBR), and Child Temperament using the Bates' infant characteristics questionnaire. Anthropometric measurements are conducted on both the child and the mother. Venous blood (3 mL) for haemoglobin testing, determination of iron indices, malaria microscopy (to be conducted at the completion of the trial) and malaria filter paper for future PCR analyses is collected from the child.

Monthly malaria infection testing

Research assistants visit the homes of participants once per month after completion of the midline visit for assessment of episodes of infection (rate, number of days affected) in the previous one month and documentation of unplanned hospital and/or healthcare facility

For peer review only

For peer review only - http://bmjopen.bmj.com/site/about/guidelines.xhtml

attendance (details of any interim visit to doctor, clinic or hospital visits in the previous month) and record of healthcare utilisation.

Endline visit (at 18 months of age (-2 weeks or +6 weeks)

The procedures are similar to the midline visit described above and in **supplementary material 2.**

Unscheduled visits

Mothers or legal guardians are asked to attend the research site/clinic when their child is symptomatically unwell. They are managed according to national standard treatment guidelines by a trained healthcare provider. Blood samples for malaria rapid diagnostic test (RDT) and blood culture are taken if clinically indicated. The clinical diagnosis of the unscheduled visit is recorded in the participant's record and captured on the study's interim visit Case Report Form (CRF) and adverse event form. If the child requires inpatient care, they are referred to the Chikwawa district hospital.

Laboratory Procedures

A capillary blood sample is collected by finger prick from all age-eligible participants to measure the haemoglobin concentration by HemoCue (HemoCue 301+, Angelholm, Sweden) and *Plasmodium* infection by rapid diagnostic test (mRDTs); (SD Bioline Malaria Ag Pf/Pan®, Standard Diagnostics) at baseline. In addition, up to 3 mL venous blood sample are collected from all enrolled participants at baseline, midline, endline and unscheduled visits (if necessary)) for haemoglobin testing, determination of iron indices (serum – for ferritin, CRP, and AGP), thick and thin malaria slides for malaria microscopy testing and filter paper for *Plasmodium* PCR. Families are encouraged to permit the collection of venous blood for venous

For peer review only

For peer review only - http://bmjopen.bmj.com/site/about/guidelines.xhtml

haemoglobin and iron and inflammation biomarkers at all time points; however, if a family refuses venous blood collection or if collection was unsuccessful, data for the primary outcome and other non-laboratory secondary outcomes will still be collected.

Data Safety and Monitoring Committee (DSMC)

An independent DSMC comprised of international experts in clinical trials, child health, infectious diseases, epidemiology, and statistics was set up to review regularly, safety data of the ongoing trial. The DSMC may recommend to the sponsor and investigators whether to continue, modify, or terminate the trial on ethical grounds.

For peer review only

Outcomes

The primary outcome

Cognitive development: assessed by the differences in Bayley-III Cognitive Composite Score (CogCS) between intervention arms measured after six months intervention (midline) and 12 months post-intervention (endline). The Bayley-III is a validated index of child development that can be administered in ~0.5-1 hour and comprises cognitive, motor, and language development scales. Assessors are specially trained to administer the tests, and inter-rater reliability is established before starting data collection. In addition, we perform 10% inter-observer testing to monitor inter-rater reliability of the measure over the study period.

Secondary outcomes

Secondary outcomes include: motor and language development assessed by Bayley-III Motor (MotCS) and Language (LangCS) Composite Scores (+6m, +12m), behaviour assessed by WBR, temperament, growth assessed by weight-for-age z-score, length-for-age z-score, weight-for-length z-score, head circumference z-score, anaemia (Hb<11g/dL), haemoglobin

For peer review only - http://bmjopen.bmj.com/site/about/guidelines.xhtml

concentration, Iron Deficiency (Ferritin<12ng/uL adjusted for inflammation assessed by CRP or AGP), ferritin concentration, and Iron Deficiency Anaemia.

Safety will be assessed by prevalence of malaria parasitaemia and C-reactive protein (CRP) and inflammation (CRP>5mg/L) at the end of the intervention and a further 6 months post intervention; episodes of caregiver reported symptoms (e.g. fever, diarrhoea, vomiting, cough) determined by the biweekly and monthly morbidity questionnaire; episodes of unplanned clinic visits and hospitalisations and deaths.

Detection and reporting of Adverse Events and Serious Adverse Events

Non-serious Adverse Events (AEs) and serious adverse events (SAEs) are collected from the time consent is given until the participant completes the study (the final visit or withdrawal). These are identified through spontaneous reports by the participant, unplanned visits to the research site or any participating health centres, observation by the study staff, and standard questioning at each visit (biweekly during the intervention period and monthly during the postintervention period. All SAEs are reported to the sponsor and the appropriate ethics committee, whether or not they are considered causally related to the investigational products.

Sample size

The primary objective will be met if either i) MNPs+DP is superior to DP alone, or ii) Iron+DP alone is superior to DP alone or iii) DP alone is superior to placebo on the Bayley-III CogCS immediately after completion of the intervention (midline). A previous study in 2853 Bangladeshi children showed that a 0.2 standardised mean difference (SMD) in Bayley-II Mental Development Index at seven months translates to a 0.7 SMD at 18 months and a 1.2

For peer review only

For peer review only - http://bmjopen.bmj.com/site/about/guidelines.xhtml

435 SMD at 64 months age.[48] Therefore, preventing a loss of at least 0.2 SMD may produce
436 magnified long-term developmental benefits as manifestations of brain dysfunction become
437 more evident. A total sample size of 2168 infants (542 per group) will provide 90% power to
438 detect an effect size of 0.25 SMD, assuming a 20% dropout and a two-sided alpha of 1.67%
439 (Bonferroni adjustment for three primary between-group comparisons).

440 **Statistical Analysis Plan**

441 A stand-alone statistical analysis plan will be detailed before breaking the blind. This plan will
442 detail all principles and precise methods of analysis, including primary and secondary
443 treatment effects of interest; alternative analyses if model assumptions do not hold; and
444 additional analyses. Participants will be analysed according to their randomised treatment
445 group for efficacy objectives and actual treatment group for safety objectives. The primary
446 outcome of Bayley-III CogCS will be analysed using a mixed-model repeated measures
447 (MMRM) analysis including in the model treatment group, time-point, and group by time-
448 point interaction. The stratification variables sex and site will be included in all models as main
449 effects. In addition, the CREDI variable (collected at baseline) will be included as a main effect
450 in the models for childhood development as it is considered prognostic for childhood
451 development outcomes. Secondary repeated continuous outcomes (e.g., growth outcomes)
452 will be analysed similarly to Bayley-III CogCS. The outcome may be appropriately transformed
453 before fitting the analysis model if considered skewed (e.g., log-transformation for ferritin).
454 Secondary repeated binary outcomes (e.g., haematologic outcomes) will be analysed using a
455 log-binomial model and account for the repeated measures with a random intercept for
456 participant. Safety (e.g., Aes, infections, hospital attendance) will be summarised during the
457 intervention and post-intervention periods separately and compared between the groups.

For peer review only - http://bmjopen.bmj.com/site/about/guidelines.xhtml

Analysis of binary repeated outcome safety data (e.g., inflammation) and continuous repeated outcome safety data (e.g., CRP) will be analysed similarly to the secondary outcomes detailed above. Episode safety data (e.g., caregiver reported diarrhoea episodes) will be analysed using negative binomial regression models. Subgroup analyses (e.g., sex, site, iron deficiency) will be performed to explore the heterogeneity of the results independent of the study findings.

Data Management

Data collected from the subjects are recorded in digital Case Report Forms (CRFs) using Redcap software (Vanderbilt University, United States) on tablets. Relevant hard copy patient hospital files are scanned for reference and stored digitally, and securely. The trial is independently monitored by an external monitoring service. No interim analysis is planned.

ETHICS AND DISSEMINATION

The National Health Science Research Committee, Malawi, and the Walter and Eliza Hall Institute of Medical Research Human Research Ethics Committee, Australia, approved this study. The Malawian Pharmacy and Medicines Regulatory Authority (PMRA) also approved the trial. Additionally, the protocol is registered with the Australian and New Zealand Clinical Trials Registry (ACTRN12620000386932). Protocol modifications are reported to the ethics committees, regulatory authorities in Malawi, investigators, trial participants and trial registries. Written informed consent in the local language is obtained from each participant before conducting any study-related procedure. In the event where the participant is illiterate, the informed consent form is read out loudly to the potential participant in the presence of an impartial witness. The potential participant thumbprints the consent form if they agree to participate in the study and the witness signs to indicate he/she witnessed the

For peer review only

For peer review only - http://bmjopen.bmj.com/site/about/guidelines.xhtml

whole consenting process. The results will be presented and shared with the local community that hosted the research and to the international forum. We will publish in peer-reviewed scientific journals and report to relevant policymaking bodies like the Malawi Ministry of Health.

DISCUSSION

The IRMA trial will provide valuable evidence on the immediate and medium-term impacts of iron interventions (iron supplements and MNPs) and malaria prevention with DP on critical functional outcomes, most importantly, child cognitive development. These findings will complement high-quality data recently reported in South Asia to provide generalisable evidence for or against universal iron interventions across sub-Saharan Africa and other malaria-endemic settings.[10]

More than two-thirds of African children aged 6-59 months are anaemic. Preventing anaemia is considered important within the first 1000 days and aligns with WHO policy for children 6-23m of age,[49] especially because the central nervous system is rapidly developing and is highly vulnerable. The prevalence of iron deficiency in children across Africa has been shown to increase from birth to approximately one year, with a peak prevalence at 9-12 months of age, and then a decrease with increasing age. Iron interventions should thus target the critical under one year period.[50]

Recent clinical trials have indicated that the combination of Intermittent preventive treatment of malaria in infants (IPTi) and malaria vaccine (RTS<S/ $AS01_E$) provides much greater protection from malaria attacks in children compared to IPTi or RTS, S/$AS01_E$ given

For peer review only - http://bmjopen.bmj.com/site/about/guidelines.xhtml

alone.[51] Combining iron interventions with IPTi, would thus be optimal for preventing both malaria and anaemia in young children in this setting.

The study's strengths include the design with cognitive development as the primary functional outcome, the large sample size with the power to detect a small effect size, and the triple dummy design, which minimizes the risk of bias. The presumed benefit of cognitive development is the key rationale for iron preventive interventions in children. Limitations include routine six monthly interval malaria microscopy testing, which is not a reliable test for the prevalence of malaria parasitaemia compared to a monthly interval. However, the strong surveillance systems on clinical symptoms (biweekly home visits and phone calls) ensured that all clinical malaria cases were picked by ensuring that all children with fever and other symptoms could access hospital care and get a rapid malaria test done.

For peer review only

For peer review only - http://bmjopen.bmj.com/site/about/guidelines.xhtml

Author Contributions:

MNM drafted the initial study protocol. MNM and GM supervised the trial's implementation and wrote the protocol paper's first draft. CCN and MDK coordinated the implementation of the study. JDH, MV and LML planned for the measurement of the primary outcome. SB, RH, and ARDM wrote the statistical analysis plan. MNM and RA provided daily scientific input during the implementation of the trial. B-AB, KSP, and S-RP conceived the trial, sought funding, and provided senior scientific oversight in the conduct of the research. All authors read and approved the final manuscript.

Funding statement: This work is supported by the National Health and Medical Research Council (NHMRC), Australia, grant numbers GNT1141185 and GNT1159151. SP is funded by NHMRC GNT1158696 and GNT2009047.

Competing interests statement.

None to declare.

Acknowledgements:

We thank all the participants of the IRMA trial - including parents or legal guardians - especially during the challenging Covid-19 period. We thank the ethics and regulatory committees mentioned in the manuscript for their extensive review and approval of the study protocol and operational documents. We acknowledge the support from the Malawi government, the Chikwawa District Health Office, including the health management teams of Chikwawa District Hospital, Makhwira, Mapelera, and Mfera Health Centres. We thank the Shire River East-and-West bank communities for hosting the study and continuously bearing with the extensive study procedures, especially the conduct of the two censuses to determine

For peer review only

For peer review only - http://bmjopen.bmj.com/site/about/guidelines.xhtml

the extent of the source population. Finally, the study would not be possible without the support of the over 100 staff members (in Malawi and Australia) who contribute their time and expertise to ensure its success. We say thank you to all.

Data Statement

All relevant data are within the paper and its supporting information files.

Protocol version

IRMA Trial protocol version 4.0 dated July 25, 2022

Ethics and regulatory approvals

Ethical approval was granted by the National Health Sciences Research Committee (#19/01/2213) in Malawi and the Human Research Ethics Committee (WEHI: 19/012) in Melbourne, Australia. In addition, the Malawi Pharmacy and Medicines Regulatory Authority (PMRA) reviewed and approved the study and the purchase and importation of the investigational products to Malawi (clinical trial certificate number PMPB/CTRC/IV/06062019111).

Trial Sponsor

Training and Research Unit of Excellence (TRUE), 1 Kufa Road, Mandala. P.O. Box 30538, Chichiri, Blantyre 3, Malawi. Email: true@true.mw

Roles and responsibilities of sponsor and funder:

The sponsor and funder have no role in the study design. They will have no part or ultimate authority in the collection, management, analysis, and interpretation of data, report writing, and the decision to submit the report for publication.

For peer review only

For peer review only - http://bmjopen.bmj.com/site/about/guidelines.xhtml

Figure heading, caption and legend

Figure 1

Figure caption and legend: *IRMA Trial schema. Overall display of IRMA activities. MNP - Iron-containing multiple micronutrient powders; DP - Dihydroartemisinin-piperaquine; Intervention – issued monthly, from enrolment at age six months to 12 months of age. Primary endpoint – Bayley's assessment conducted at the trial midline; Cognitive assessments – Bayley Scales of Infant and Toddler Development 3rd Edition.*

For peer review only

For peer review only - http://bmjopen.bmj.com/site/about/guidelines.xhtml

References

1. World Health Organization. The global prevalence of anaemia in 2011. Geneva, Switzerland: WHO, 2015.
2. Stevens GA, Paciorek CJ, Flores-Urrutia MC, et al. National, regional, and global estimates of anaemia by severity in women and children for 2000-19: a pooled analysis of population-representative data. *Lancet Glob Health* 2022;10(5):e627-e39. doi: 10.1016/s2214-109x(22)00084-5 [published Online First: 2022/04/16]
3. World Health Organization. WHO Global anaemia estimates, 2021 Edition. Global anaemia estimates in women of reproductive age, by pregnancy status, and in children aged 6-59 months. Geneva, Switzerland: WHO; 2021 [cited 2022 28 August]. Available from: https://www.who.int/data/gho/data/themes/topics/anaemia_in_women_and_children accessed 28 August 2022.
4. National Statistical Office (NSO) [Malawi] and ICF. Malawi Demographic and Health Survey 2015-16,. Zomba, Malawi and Rockville, Maryland, USA: NSO and ICF, 2017.
5. Institute for Health Metrics and Evaluation (IHME). Global Burden of Disease (GBD) 2022 [cited 2022. Available from: https://www.healthdata.org/gbd/2019 accessed 28 August 2022.
6. World Health Organization. Use of multiple micronutrient powders for point-of-use fortification of foods consumed by infants and young children aged 6-23 months and children aged 2-12 years. Geneva, Switzerland: WHO, 2016a.
7. World Health Organization. Guideline: Daily iron supplementation in infants and children. Geneva, Switzerland: WHO, 2016b.
8. World Health Organization, United Nations Children's Fund, United Nations University. Iron Deficiency Anaemia: Assessment, Prevention, and Control. A guide for programme managers. Geneva, Switzerland: WHO, 2001.
9. UNICEF. Multiple Micronutrient Powder: Supply and market update - July 2021. New York: UNICEF, 2021.
10. Pasricha SR, Hasan MI, Braat S, et al. Benefits and Risks of Iron Interventions in Infants in Rural Bangladesh. *N Engl J Med* 2021;385(11):982-95. doi: 10.1056/NEJMoa2034187 [published Online First: 2021/09/09]
11. Pasricha SR, Hayes E, Kalumba K, et al. Effect of daily iron supplementation on health in children aged 4-23 months: a systematic review and meta-analysis of randomised controlled trials. *Lancet Glob Health* 2013;1(2):e77-e86. doi: 10.1016/s2214-109x(13)70046-9 [published Online First: 2014/08/12]
12. De-Regil LM, Suchdev PS, Vist GE, et al. Home fortification of foods with multiple micronutrient powders for health and nutrition in children under two years of age. *Cochrane Database Syst Rev* 2011(9):Cd008959. doi: 10.1002/14651858.CD008959.pub2 [published Online First: 2011/09/09]
13. Akman M, Cebeci D, Okur V, et al. The effects of iron deficiency on infants' developmental test performance. *Acta Paediatr* 2004;93(10):1391-6. [published Online First: 2004/10/27]
14. Idjradinata P, Pollitt E. Reversal of developmental delays in iron-deficient anaemic infants treated with iron. *Lancet* 1993;341(8836):1-4. doi: 10.1016/0140-6736(93)92477-b [published Online First: 1993/01/02]

For peer review only - http://bmjopen.bmj.com/site/about/guidelines.xhtml

15. Adu-Afarwuah S, Lartey A, Brown KH, et al. Home fortification of complementary foods with micronutrient supplements is well accepted and has positive effects on infant iron status in Ghana. *Am J Clin Nutr* 2008;87(4):929-38. doi: 10.1093/ajcn/87.4.929 [published Online First: 2008/04/11]
16. Somassè YE, Dramaix M, Traoré B, et al. The WHO recommendation of home fortification of foods with multiple-micronutrient powders in children under 2 years of age and its effectiveness on anaemia and weight: a pragmatic cluster-randomized controlled trial. *Public Health Nutr* 2018;21(7):1350-58. doi: 10.1017/s1368980017003858 [published Online First: 2018/01/22]
17. Sazawal S, Black RE, Ramsan M, et al. Effects of routine prophylactic supplementation with iron and folic acid on admission to hospital and mortality in preschool children in a high malaria transmission setting: community-based, randomised, placebo-controlled trial. *Lancet* 2006;367(9505):133-43. doi: 10.1016/s0140-6736(06)67962-2 [published Online First: 2006/01/18]
18. Veenemans J, Milligan P, Prentice AM, et al. Effect of supplementation with zinc and other micronutrients on malaria in Tanzanian children: a randomised trial. *PLoS Med* 2011;8(11):e1001125. doi: 10.1371/journal.pmed.1001125 [published Online First: 2011/12/02]
19. Neuberger A, Okebe J, Yahav D, et al. Oral iron supplements for children in malaria-endemic areas. *Cochrane Database Syst Rev* 2016;2(2):Cd006589. doi: 10.1002/14651858.CD006589.pub4 [published Online First: 2016/02/28]
20. Jonker FA, Calis JC, van Hensbroek MB, et al. Iron status predicts malaria risk in Malawian preschool children. *PLoS One* 2012;7(8):e42670. doi: 10.1371/journal.pone.0042670 [published Online First: 2012/08/24]
21. Clark MA, Goheen MM, Fulford A, et al. Host iron status and iron supplementation mediate susceptibility to erythrocytic stage Plasmodium falciparum. *Nat Commun* 2014;5:4446. doi: 10.1038/ncomms5446 [published Online First: 2014/07/26]
22. Goheen MM, Wegmüller R, Bah A, et al. Anemia Offers Stronger Protection Than Sickle Cell Trait Against the Erythrocytic Stage of Falciparum Malaria and This Protection Is Reversed by Iron Supplementation. *EBioMedicine* 2016;14:123-30. doi: 10.1016/j.ebiom.2016.11.011 [published Online First: 2016/11/18]
23. Calis JC, Phiri KS, Faragher EB, et al. Severe anemia in Malawian children. *N Engl J Med* 2008;358(9):888-99. doi: 10.1056/NEJMoa072727 [published Online First: 2008/02/29]
24. Newton CR, Warn PA, Winstanley PA, et al. Severe anaemia in children living in a malaria endemic area of Kenya. *Trop Med Int Health* 1997;2(2):165-78. doi: 10.1046/j.1365-3156.1997.d01-238.x [published Online First: 1997/02/01]
25. Njua-Yafi C, Achidi EA, Anchang-Kimbi JK, et al. Malaria, helminths, co-infection and anaemia in a cohort of children from Mutengene, south western Cameroon. *Malar J* 2016;15:69. doi: 10.1186/s12936-016-1111-2 [published Online First: 2016/02/08]
26. Pasricha SR, Atkinson SH, Armitage AE, et al. Expression of the iron hormone hepcidin distinguishes different types of anemia in African children. *Sci Transl Med* 2014;6(235):235re3. doi: 10.1126/scitranslmed.3008249 [published Online First: 2014/05/09]
27. Zlotkin S, Newton S, Aimone AM, et al. Effect of iron fortification on malaria incidence in infants and young children in Ghana: a randomized trial. *Jama* 2013;310(9):938-47. doi: 10.1001/jama.2013.277129 [published Online First: 2013/09/05]

For peer review only

For peer review only - http://bmjopen.bmj.com/site/about/guidelines.xhtml

28. Pasricha SR, Gheorghe A, Sakr-Ashour F, et al. Net benefit and cost-effectiveness of universal iron-containing multiple micronutrient powders for young children in 78 countries: a microsimulation study. *Lancet Glob Health* 2020;8(8):e1071-e80. doi: 10.1016/s2214-109x(20)30240-0 [published Online First: 2020/07/28]
29. Fernando SD, Rodrigo C, Rajapakse S. The 'hidden' burden of malaria: cognitive impairment following infection. *Malar J* 2010;9:366. doi: 10.1186/1475-2875-9-366 [published Online First: 2010/12/22]
30. Clarke SE, Jukes MC, Njagi JK, et al. Effect of intermittent preventive treatment of malaria on health and education in schoolchildren: a cluster-randomised, double-blind, placebo-controlled trial. *Lancet (London, England)* 2008;372(9633):127-38. doi: 10.1016/s0140-6736(08)61034-x [published Online First: 2008/07/16]
31. Jukes MCH, Pinder M, Grigorenko EL, et al. Long-Term Impact of Malaria Chemoprophylaxis on Cognitive Abilities and Educational Attainment: Follow-Up of a Controlled Trial. *PLOS Clinical Trials* 2006;1(4):e19. doi: 10.1371/journal.pctr.0010019
32. Meremikwu MM, Donegan S, Sinclair D, et al. Intermittent preventive treatment for malaria in children living in areas with seasonal transmission. *The Cochrane database of systematic reviews* 2012(2):Cd003756. doi: 10.1002/14651858.CD003756.pub4 [published Online First: 2012/02/18]
33. WHO. Guidelines for the treatment of Malaria Third edition ed: World Health Organisation 2015.
34. Bigira V, Kapisi J, Clark TD, et al. Protective Efficacy and Safety of Three Antimalarial Regimens for the Prevention of Malaria in Young Ugandan Children: A Randomized Controlled Trial. *PLoS medicine* 2014;11(8):e1001689. doi: 10.1371/journal.pmed.1001689
35. World malaria report 2017: World Health Organisation, 2017.
36. Zgambo M, Mbakaya BC, Kalembo FW. Prevalence and factors associated with malaria parasitaemia in children under the age of five years in Malawi: A comparison study of the 2012 and 2014 Malaria Indicator Surveys (MISs). *PloS one* 2017;12(4):e0175537. doi: 10.1371/journal.pone.0175537 [published Online First: 2017/04/12]
37. WHO. Malawi: WHO statistical profile: World Health Organisation 2015.
38. Whitson D. Farta Child Survival Project: Final Evaluation: CARE Ethiopia, 2007.
39. Bayley N. Bayley Scales of Infant and Toddler Development, Third Edition (Bayley--III®) [Database record]. APA PsycTests., 2005.
40. National Statistical Office (NSO) and ICF Macro. Malawi Demographic and Health Survey 2010. Zomba, Malawi, and Calverton, Maryland, USA: National Statistical Office (NSO) and ICF Macro 2011.
41. National Malaria Control Programme (NMCP) [Malawi] and ICF International. Malawi Malaria Indicator Survey (MIS) 2014. Lilongwe, Malawi, and Rockville, Maryland, USA: NMCP and ICF International 2014.
42. WHO Multicentre Growth Reference Study Group. Assessment of differences in linear growth among populations in the WHO Multicentre Growth Reference Study. *Acta Paediatr Suppl* 2006;450:56-65. doi: 10.1111/j.1651-2227.2006.tb02376.x [published Online First: 2006/07/05]

For peer review only - http://bmjopen.bmj.com/site/about/guidelines.xhtml

43. Hamadani JD, Tofail F, Hilaly A, et al. Use of family care indicators and their relationship with child development in Bangladesh. *J Health Popul Nutr* 2010;28(1):23-33. doi: 10.3329/jhpn.v28i1.4520 [published Online First: 2010/03/11]
44. Radloff LS. The CES-D Scale: A Self-Report Depression Scale for Research in the General Population. . *Applied Psychological Measurement* 1977;1(3):385–401. doi: https://doi.org/10.1177/014662167700100306
45. Coates Jennifer, Anne Swindale, Paula Bilinsky. Household Food Insecurity Access Scale (HFIAS)for Measurement of Household Food Access: Indicator Guide (v. 3). Washington, D.C.: FHI 360/FANTA, 2007.
46. Swindale Anne, Paula Bilinsky. Household Dietary Diversity Score (HDDS) for Measurement of Household Food Access: Indicator Guide (v.2). Washington, D.C.: FHI 360/FANTA, 2006.
47. McCoy DC, Sudfeld CR, Bellinger DC, et al. Development and validation of an early childhood development scale for use in low-resourced settings. *Popul Health Metr* 2017;15(1):3. doi: 10.1186/s12963-017-0122-8 [published Online First: 2017/02/12]
48. Hamadani JD, Tofail F, Huda SN, et al. Cognitive deficit and poverty in the first 5 years of childhood in Bangladesh. *Pediatrics* 2014;134(4):e1001-8. doi: 10.1542/peds.2014-0694 [published Online First: 2014/10/01]
49. WHO. Guideline: Daily iron supplementation in infants and children. Geneva: World Health Organization, 2016.
50. Muriuki JM, Mentzer AJ, Webb EL, et al. Estimating the burden of iron deficiency among African children. *BMC Med* 2020;18(1):31. doi: 10.1186/s12916-020-1502-7 [published Online First: 2020/02/28]
51. Greenwood B, Cairns M, Chaponda M, Chico RM, Dicko A, Ouedraogo JB, Phiri KS, Ter Kuile FO, Chandramohan D. Combining malaria vaccination with chemoprevention: a promising new approach to malaria control. Malar J. 2021 Sep 6;20(1):361. doi: 10.1186/s12936-021-03888-8. PMID: 34488784; PMCID: PMC8419817.

For peer review only - http://bmjopen.bmj.com/site/about/guidelines.xhtml

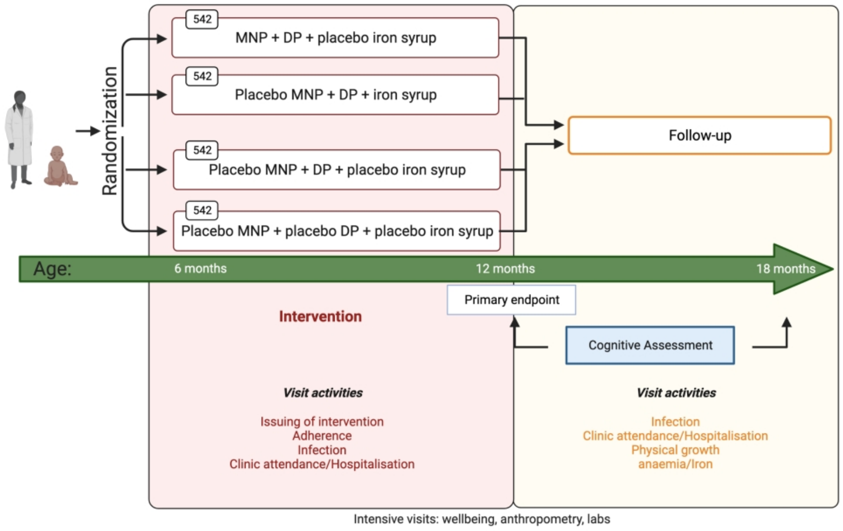

Figure 1: IRMA study design

645x516mm (118 x 118 DPI)

Supplementary material 1: Multiple micronutrient powders (MNPs) contents

	**Micronutrient name**	**Quantity**
1.	Niacinamide	7.02 - 8.58 mg/g
2.	Riboflavin	0.653 - 0.798 mg/g
3.	Pyridoxine	0.585 – 0.715 mg/g
4.	Thiamine	0.675 – 0.825 mg/g
5.	Vitamin C	32.4 – 39.6 mg/g
6.	Vitamin A	522 – 667 µgRE/g
7.	Vitamin E (as Tocopherol Eq.)	5.04 – 6.16 mgTE/g
8.	Vitamin D3	6.75 – 8.63 µgD/g
9.	Vitamin B12	1.09 – 1.40 µg/g
10.	Folic Acid	109 – 134 µg/g
11.	Selenium	17.0 – 24.5 µg/g
12.	Iodine	9.00 -129.6 µg/g
13.	Iron	10.00 -12.10 mg/g
14.	Zinc	4.10 – 0.739 mg/g
15.	Copper	0.605 – 0.739 mg/g

For peer review only

Supplementary material 2: Main study activities per visit

		Intervention period (Active follow-up)													Post intervention period (Passive follow up)					
	**Month**	**M1**			**M2**		**M3**		**M4**		**M5**		**M6**		**M7**	**M8**	**M9**	**M10**	**M11**	**M12[‡]**
**Planned activity**	**Day** 0	D 1[#]	D 14	D 28	D 42	D 56	D 70	D 84	D 98	D 112	D 126	D 140	D 154	D 168[§]						
**Location**	**H/HF**	**HF**	**H**	**H**	**H**	**H**	**H**	**H**	**H**	**H**	**H**	**H**	**H**	**HF**	**H**	**H**	**H**	**H**	**H**	**HF**
Pre-screening/ screening, & informed consent	**X**	**X**																		
Randomization		**X**																		
Demographic, clinical, CREDI and SES assessments		**X**																		
Family Care Index (FCI)		**X**												**X**						**X**
Bayley-III Cognitive Composite Score (CogCS)														**X**						**X**
Bayley-III Motor (MotCS) and Language (LangCS) Composite Scores														**X**						**X**
Behaviour: Wolke's Behaviour Ratings (WBR)														**X**						**X**
Food security & dietary diversity questionnaires		**X**												**X**						**X**
Anthropometry[1] - (mother and child)		**X**												**X**						**X**
**Laboratory procedures**																				
Haemoglobin		**X**												**X**						**X**
Malaria microscopy		**X**												**X**						**X**

For peer review only

For peer review only - http://bmjopen.bmj.com/site/about/guidelines.xhtml

Serum iron marker tests[2]		X												X						X
Serum for inflammatory marker tests (CRP/AGP)[3]		X												X						X
**Active follow-up:**																				
Compliance assessment		X	X	X	X	X	X	X	X	X	X	X	X	X						
MNPs/Placebo MNPs[4](distribute, instruct)		X		X		X		X		X		X								
DP/Placebo DP[5] (distribute, instruct & observe use/intake)		X		X		X		X		X		X								
Iron syrup/Placebo iron syrup[6]		X		X		X		X		X		X								
Provide educational information		X	X	X	X	X	X	X	X	X	X	X	X	X						
Assess infections[7]			X	X	X	X	X	X	X	X	X	X	X	X	X	X	X	X	X	X
Adverse Events monitoring			X	X	X	X	X	X	X	X	X	X	X	X	X	X	X	X	X	X
Health care utilization monitoring		X	X	X	X	X	X	X	X	X	X	X	X	X	X	X	X	X	X	X

# - Baseline; § - Midline visit at day 168. Primary outcome was assessed at day 168 ± 14 days as per protocol)**; ‡ - Endline; M –** Month; **D –** Day; **H – Home** (visit at the Participant's Home)**; HF –** Visit at the Health Facility; **X** – scheduled activity. **CREDI -** Caregiver Reported Early Development Instrument**; SES -** Social Economic Status; [1]**Anthropometric assessments** (mother and child): height/length, weight; [2]**Serum iron marker tests**: Serum ferritin; [3]**Inflammation markers**: CRP (C-Reactive Protein) or AGP (alpha 1 acid glycoprotein); [4]**MNPs** containing 10.0mg iron and 14 other micronutrients, daily for 6 months; **Placebo MNPs**: (maltodextrin), daily for 6 months; [5]**DP/Placebo DP** (20mg dihydroartemisinin/ 160mg piperaquine in suspension) dosed by body weight, three consecutive days every 4 weeks for 6 months; [6]**Iron syrup/Placebo iron syrup:** 10mg daily for six months; [7]**Infections**(assessment): (rate and number of days affected) fever/respiratory/diarrhoea.
NB: Families were encouraged to permit collection of venous blood for venous haemoglobin and iron and inflammation biomarkers at all timepoints; however, if a family refused venous blood collection or if collection was unsuccessful, data for the primary outcome and other non-laboratory secondary outcomes was still collected.

For peer review only

Reporting checklist for protocol of a clinical trial.

Based on the SPIRIT guidelines.

Instructions to authors

Complete this checklist by entering the page numbers from your manuscript where readers will find each of the items listed below.

Your article may not currently address all the items on the checklist. Please modify your text to include the missing information. If you are certain that an item does not apply, please write "n/a" and provide a short explanation.

Upload your completed checklist as an extra file when you submit to a journal.

In your methods section, say that you used the SPIRITreporting guidelines, and cite them as:

Chan A-W, Tetzlaff JM, Gøtzsche PC, Altman DG, Mann H, Berlin J, Dickersin K, Hróbjartsson A, Schulz KF, Parulekar WR, Krleža-Jerić K, Laupacis A, Moher D. SPIRIT 2013 Explanation and Elaboration: Guidance for protocols of clinical trials. BMJ. 2013;346:e7586

For peer review only

		Reporting Item	Page Number
**Administrative information**			
Title	#1	Descriptive title identifying the study design, population, interventions, and, if applicable, trial acronym	1
Trial registration	#2a	Trial identifier and registry name. If not yet registered, name of intended registry	4
Trial registration: data set	#2b	All items from the World Health Organization Trial Registration Data Set	4
Protocol version	#3	Date and version identifier	31
Funding	#4	Sources and types of financial, material, and other support	31
Roles and responsibilities: contributorship	#5a	Names, affiliations, and roles of protocol contributors	30

Roles and responsibilities: sponsor contact information	#5b	Name and contact information for the trial sponsor	31
Roles and responsibilities: sponsor and funder	#5c	Role of study sponsor and funders, if any, in study design; collection, management, analysis, and interpretation of data; writing of the report; and the decision to submit the report for publication, including whether they will have ultimate authority over any of these activities	31-32
Roles and responsibilities: committees	#5d	Composition, roles, and responsibilities of the coordinating centre, steering committee, endpoint adjudication committee, data management team, and other individuals or groups overseeing the trial, if applicable (see Item 21a for data monitoring committee)	22-23
**Introduction**			
Background and rationale	#6a	Description of research question and justification for undertaking the trial, including summary of relevant studies (published and unpublished) examining benefits and harms for each intervention	6-10
Background and rationale: choice of comparators	#6b	Explanation for choice of comparators	14
Objectives	#7	Specific objectives or hypotheses	10-12
Trial design	#8	Description of trial design including type of trial (eg, parallel group, crossover, factorial, single group), allocation ratio, and framework (eg, superiority, equivalence, non-inferiority, exploratory)	12
**Methods: Participants, interventions, and outcomes**			
Study setting	#9	Description of study settings (eg, community clinic, academic hospital) and list of countries where data will be collected. Reference to where list of study sites can be obtained	12
Eligibility criteria	#10	Inclusion and exclusion criteria for participants. If applicable, eligibility criteria for study centres and individuals who will	12-13

For peer review only

		perform the interventions (eg, surgeons, psychotherapists)	
Interventions: description	#11a	Interventions for each group with sufficient detail to allow replication, including how and when they will be administered	14
Interventions: modifications	#11b	Criteria for discontinuing or modifying allocated interventions for a given trial participant (eg, drug dose change in response to harms, participant request, or improving / worsening disease)	14
Interventions: adherance	#11c	Strategies to improve adherence to intervention protocols, and any procedures for monitoring adherence (eg, drug tablet return; laboratory tests)	20
Interventions: concomitant care	#11d	Relevant concomitant care and interventions that are permitted or prohibited during the trial	12-13
Outcomes	#12	Primary, secondary, and other outcomes, including the specific measurement variable (eg, systolic blood pressure), analysis metric (eg, change from baseline, final value, time to event), method of aggregation (eg, median, proportion), and time point for each outcome. Explanation of the clinical relevance of chosen efficacy and harm outcomes is strongly recommended	23
Participant timeline	#13	Time schedule of enrolment, interventions (including any run-ins and washouts), assessments, and visits for participants. A schematic diagram is highly recommended (see Figure)	15-22
Sample size	#14	Estimated number of participants needed to achieve study objectives and how it was determined, including clinical and statistical assumptions supporting any sample size calculations	24
Recruitment	#15	Strategies for achieving adequate participant enrolment to reach target sample size	15
**Methods: Assignment of interventions (for controlled trials)**			
Allocation: sequence generation	#16a	Method of generating the allocation sequence (eg, computer-generated random numbers), and list of any factors for stratification. To reduce predictability of a random sequence, details of any planned restriction (eg, blocking) should be provided in a separate document that is unavailable to those who enrol participants or assign interventions	14-15

For peer review only

Allocation concealment mechanism	#16b	Mechanism of implementing the allocation sequence (eg, central telephone; sequentially numbered, opaque, sealed envelopes), describing any steps to conceal the sequence until interventions are assigned	14-15
Allocation: implementation	#16c	Who will generate the allocation sequence, who will enrol participants, and who will assign participants to interventions	14-15
Blinding (masking)	#17a	Who will be blinded after assignment to interventions (eg, trial participants, care providers, outcome assessors, data analysts), and how	14-15
Blinding (masking): emergency unblinding	#17b	If blinded, circumstances under which unblinding is permissible, and procedure for revealing a participant's allocated intervention during the trial	14-15
**Methods: Data collection, management, and analysis**			
Data collection plan	#18a	Plans for assessment and collection of outcome, baseline, and other trial data, including any related processes to promote data quality (eg, duplicate measurements, training of assessors) and a description of study instruments (eg, questionnaires, laboratory tests) along with their reliability and validity, if known. Reference to where data collection forms can be found, if not in the protocol	15-22
Data collection plan: retention	#18b	Plans to promote participant retention and complete follow-up, including list of any outcome data to be collected for participants who discontinue or deviate from intervention protocols	15-22
Data management	#19	Plans for data entry, coding, security, and storage, including any related processes to promote data quality (eg, double data entry; range checks for data values). Reference to where details of data management procedures can be found, if not in the protocol	24-25
Statistics: outcomes	#20a	Statistical methods for analysing primary and secondary outcomes. Reference to where other details of the statistical analysis plan can be found, if not in the protocol	24
Statistics: additional analyses	#20b	Methods for any additional analyses (eg, subgroup and adjusted analyses)	24

For peer review only

Statistics: analysis population and missing data	#20c	Definition of analysis population relating to protocol non-adherence (eg, as randomised analysis), and any statistical methods to handle missing data (eg, multiple imputation)	24
**Methods: Monitoring**			
Data monitoring: formal committee	#21a	Composition of data monitoring committee (DMC); summary of its role and reporting structure; statement of whether it is independent from the sponsor and competing interests; and reference to where further details about its charter can be found, if not in the protocol. Alternatively, an explanation of why a DMC is not needed	22-23
Data monitoring: interim analysis	#21b	Description of any interim analyses and stopping guidelines, including who will have access to these interim results and make the final decision to terminate the trial	25
Harms	#22	Plans for collecting, assessing, reporting, and managing solicited and spontaneously reported adverse events and other unintended effects of trial interventions or trial conduct	23-24
Auditing	#23	Frequency and procedures for auditing trial conduct, if any, and whether the process will be independent from investigators and the sponsor	25
**Ethics and dissemination**			
Research ethics approval	#24	Plans for seeking research ethics committee / institutional review board (REC / IRB) approval	26
Protocol amendments	#25	Plans for communicating important protocol modifications (eg, changes to eligibility criteria, outcomes, analyses) to relevant parties (eg, investigators, REC / IRBs, trial participants, trial registries, journals, regulators)	26
Consent or assent	#26a	Who will obtain informed consent or assent from potential trial participants or authorised surrogates, and how (see Item 32)	19
Consent or assent: ancillary studies	#26b	Additional consent provisions for collection and use of participant data and biological specimens in ancillary studies, if applicable	26
Confidentiality	#27	How personal information about potential and enrolled participants will be collected, shared, and maintained in order to protect confidentiality before, during, and after the trial	26

For peer review only

Declaration of interests	#28	Financial and other competing interests for principal investigators for the overall trial and each study site	30
Data access	#29	Statement of who will have access to the final trial dataset, and disclosure of contractual agreements that limit such access for investigators	25
Ancillary and post trial care	#30	Provisions, if any, for ancillary and post-trial care, and for compensation to those who suffer harm from trial participation	
Dissemination policy: trial results	#31a	Plans for investigators and sponsor to communicate trial results to participants, healthcare professionals, the public, and other relevant groups (eg, via publication, reporting in results databases, or other data sharing arrangements), including any publication restrictions	22
Dissemination policy: authorship	#31b	Authorship eligibility guidelines and any intended use of professional writers	30
Dissemination policy: reproducible research	#31c	Plans, if any, for granting public access to the full protocol, participant-level dataset, and statistical code	31
**Appendices**			
Informed consent materials	#32	Model consent form and other related documentation given to participants and authorised surrogates	26
Biological specimens	#33	Plans for collection, laboratory evaluation, and storage of biological specimens for genetic or molecular analysis in the current trial and for future use in ancillary studies, if applicable	22

None The SPIRIT Explanation and Elaboration paper is distributed under the terms of the Creative Commons Attribution License CC-BY-NC. This checklist can be completed online using https://www.goodreports.org/, a tool made by the EQUATOR Network in collaboration with Penelope.ai

For peer review only

BMJ Open

Protocol for a randomised, multicentre, four-arm, double-blinded, placebo-controlled trial to assess the benefits and safety of Iron supplementation with Malaria chemoprevention to children in Malawi - IRMA trial

Journal:	*BMJ Open*
Manuscript ID	bmjopen-2022-069011.R3
Article Type:	Protocol
Date Submitted by the Author:	15-Sep-2023
Complete List of Authors:	Mwangi, Martin; Training and Research Unit of Excellence (TRUE), Nutrition and Infectious Diseases ; The Micronutrient Forum, Healthy Mothers Healthy Babies Consortium<br>Mzembe, Glory; Training and Research Unit of Excellence (TRUE), Nutrition and Infectious Diseases ; Kamuzu University of Health Sciences, Public Health<br>Ngwira, Chikondi ; Training and Research Unit of Excellence (TRUE), Nutrition and Infectious Diseases ; Liverpool School of Tropical Medicine<br>Vokhiwa, Maclean; Training and Research Unit of Excellence (TRUE), Nutrition and Infectious Diseases<br>Kapulula, Mayamiko; Training and Research Unit of Excellence (TRUE), Nutrition and Infectious Diseases<br>Larson, Leila; University of South Carolina Arnold School of Public Health, Department of Health Promotion, Education, and Behaviour<br>Braat, Sabine ; University of Melbourne School of Population and Global Health, Centre for Epidemiology and Biostatistics; Walter and Eliza Hall Institute of Medical Research<br>Harding, Rebecca; The Walter and Eliza Hall Institute of Medical Research,<br>McLean, Alistair; Walter and Eliza Hall Institute of Medical Research; The University of Melbourne, Department of Infectious Diseases<br>Hamadani, Jena; International Centre for Diarrhoeal Disease Research<br>Biggs, Beverley-Ann ; The University of Melbourne, Medicine<br>Ataíde, Ricardo; The Walter and Eliza Hall Institute of Medical Research; The University of Melbourne, Department of Infectious Diseases at the Peter Doherty Institute of Infection and Immunity<br>Phiri, Kamija; Training and Research Unit of Excellence (TRUE), Nutrition and Infectious Diseases ; Kamuzu University of Health Sciences, Public Health<br>Pasricha, Sant-Rayn; Walter and Eliza Hall Institute of Medical Research,
**Primary Subject Heading**:	Public health
Secondary Subject Heading:	Haematology (incl blood transfusion)
Keywords:	Anaemia < HAEMATOLOGY, Infection control < INFECTIOUS DISEASES, PUBLIC HEALTH, Nutrition < TROPICAL MEDICINE

SCHOLARONE™
Manuscripts

Protocol for a randomised, multicentre, four-arm, double-blinded, placebo-controlled trial to assess the benefits and safety of Iron supplementation with Malaria chemoprevention to children in Malawi - IRMA trial

Authors

*Martin N. Mwangi[1,2], *Glory Mzembe[1,3], Chikondi C. Ngwira[1], Maclean Vokhiwa[1,3], Mayamiko D. Kapulula[1], Leila M. Larson[4], Sabine Braat[5,6,7], Rebecca Harding[7], Alistair R.D. McLean[6,7], Jena D. Hamadani[8], Beverley-Ann Biggs[5,9], Ricardo Ataíde[5,7], Kamija S. Phiri[1,3], Sant-Rayn Pasricha [7,9,10]

*Joint first authors

Corresponding author:

Martin N. Mwangi, Training and Research Unit of Excellence (TRUE), 1 Kufa Road, Post Office Box 30538, Blantyre, Malawi. Email: mmwangi@true.mw

Author Institutions

1. Training and Research Unit of Excellence (TRUE), Blantyre, Malawi.
2. The Micronutrient Forum, Healthy Mothers Healthy Babies Consortium, Washington DC, USA.
3. Department of Public Health, School of Public and Global Health, Kamuzu University of Health Sciences, Blantyre, Malawi.
4. Department of Health Promotion, Education, and Behaviour, Arnold School of Public Health, University of South Carolina, Columbia, South Carolina, USA.
5. Department of Infectious Diseases, University of Melbourne, at the Peter Doherty Institute for Infection and Immunity, Melbourne, Australia.

For peer review only - http://bmjopen.bmj.com/site/about/guidelines.xhtml

For peer review only

6. Centre for Epidemiology and Biostatistics, Melbourne School of Population and Global Health, The University of Melbourne, Melbourne, Australia.

7. Population Health and Immunity Division, The Walter and Eliza Hall Institute of Medical Research, Melbourne, Australia.

8. International Centre for Diarrhoeal Disease Research, Bangladesh, Dhaka, Bangladesh.

9. Diagnostic Haematology, The Royal Melbourne Hospital; and Clinical Haematology, Melbourne, Australia.

10. Department of Medical Biology, Faculty of Medicine, Dentistry and Health Sciences, The University of Melbourne, Melbourne, Australia.

Word count: 4971. (Abstract) 298 words. *(excluding title page, abstract, references, figures and tables)*

For peer review only

For peer review only - http://bmjopen.bmj.com/site/about/guidelines.xhtml

ABSTRACT

Introduction: Approximately 40% of children 6-59 months of age worldwide are anaemic. Iron-containing multiple micronutrient powders (MNPs) and iron supplements (syrup/drops) are used to combat anaemia in children in different parts of the world. However, evidence for functional benefits of iron supplementation in children is scarce, and potential risks remain poorly defined, particularly concerning diarrhoea and malaria. The trial aims to determine if: a) the efficacy of iron supplements or MNPs (containing iron) given with malaria chemoprevention is superior to malaria chemoprevention alone, or b) if the efficacy of malaria chemoprevention alone is superior to placebo on child cognitive development.

Methods and Analysis: IRMA is a four-arm, parallel-group, double-blinded, placebo-controlled, triple-dummy, randomised trial in Southern Malawi. The study recruits 2,168 infants aged six months, with an intervention period of six months and a post-intervention period of a further six months. Children are randomised into four arms: (a) No intervention (placebo); (b) malaria chemoprevention only; (c) MNPs and Malaria chemoprevention; and (d) Iron syrup and malaria chemoprevention. The primary outcome, cognitive development (CogCS), is measured at the end of the six months intervention. Secondary outcomes include CogCS at a further six months post-intervention, motor, language and behavioural development, physical growth, and prevalence of anaemia and iron deficiency. Safety outcomes include incidence of malaria and other infections, and prevalence of malaria parasitaemia during and post-intervention period.

Ethics and dissemination: The trial is approved by the National Health Sciences Research Committee (#19/01/2213) (Malawi) and the Human Research Ethics Committee (WEHI: 19/012) (Australia). Written informed consent in the local language is obtained from each

For peer review only - http://bmjopen.bmj.com/site/about/guidelines.xhtml

For peer review only

participant before conducting any study-related procedure. Results will be shared with the local community and internationally with academic and policy stakeholders.

Protocol registration details: The protocol is registered with the Australian and New Zealand Clinical Trials Registry (ACTRN12620000386932).

Keywords: Anaemia, Infant, Infection, Iron Deficiency, Malaria, Malawi, Micronutrient Powders, randomised controlled trial.

Strengths and limitations of this study

- The trial setting represents malaria-endemic Sub-Saharan African countries where anaemia and malaria co-exist, which is an ideal place to study the benefits versus risks of iron supplementation (under malaria chemoprevention) to establish a generalisable clinical rationale.
- The participant screening and inclusion criteria involve all infants within the recruitment age range across a large geographical catchment area which enables the generalizability of the study results to most national programs in malaria-endemic countries.
- The trial compares two major delivery modes for iron in young children: MNPs and iron syrup which will help identify the ideal and most effective iron source for children.
- The trial utilises a functional clinical health outcome – child cognitive development – as its primary outcome, ensuring the trial provides evidence of the functional relevance of universal iron interventions and malaria prevention.
- The trial is powered to detect a small but clinically relevant effect for the primary outcome of child cognitive development; the use of a post-intervention follow-up

For peer review only

For peer review only - http://bmjopen.bmj.com/site/about/guidelines.xhtml

period enables the assessment of both the immediate and medium-term impact of the intervention.

INTRODUCTION

Anaemia remains a critical global health problem affecting up to 39.8% of children aged 6 – 59 months worldwide and 60.2% in Africa.[1–3] In Malawi, 63% of children 6-59 months are anaemic, with a peak prevalence of 91% observed among children aged 9-11 months.[4] Iron deficiency accounts for most (60%) of the global burden of anaemia[5] but less so in malaria-endemic areas, where only 32% of childhood anaemia is iron responsive.[1]

World Health Organisation (WHO) anaemia prevention guidelines recommend two universal iron supplementation strategies for children, namely; 1) iron supplement drops or syrups (10-12.5mg of elemental iron) – given daily to children 6-24 months in areas where the prevalence of anaemia is 40% or higher; and 2) iron-containing multiple micronutrient powders (MNPs): 10-12.5mg of iron – given daily over six months in areas where the prevalence of anaemia is 20% or higher.[6–8] However, no recommendation is given regarding the preferred approach. MNPs are the most common form of iron supplementation, with at least 58 countries reported to have implemented MNPs home fortification by 2021.[9] The rationale for universal iron supplementation is an understanding that iron supplementation would reduce childhood anaemia and improve child development and other functional health outcomes. However, we recently showed that in a rural South Asian population, neither universal distribution of oral iron supplements nor MNPs meaningfully improved immediate or medium-term child cognitive, language, motor, and other developmental outcomes, even though anaemia and iron deficiency were reduced.[10] However, these data were generated

For peer review only - http://bmjopen.bmj.com/site/about/guidelines.xhtml

from a trial in a non-malaria endemic part of South Asia, where the determinants of anaemia and infection risk are likely very different.

Benefits of universal iron supplementation programmes

Universal iron supplementation programs have shown benefits in haematological outcomes. A meta-analysis[11] and a Cochrane review[12] showed improvements in anaemia with daily iron supplementation in children. However, the effects on growth and development are less clear. Few studies have investigated the impact on cognitive development. Only 8 of the 33 studies included in the meta-analysis by Pasricha et al. assessed cognitive development, and no significant beneficial effect was seen (mean difference 1.65 [95% CI -0.63, 3.94]), but data were sparse.[11] Subgroup analysis did suggest a benefit of iron supplementation on cognitive development in iron-deficient children. However, this data came from only two studies.[13,14] Only one study in the Cochrane review assessed child development, showing that children receiving MNPs were more likely to walk independently at 12 months of age than those receiving no intervention.[15] There is also little evidence for a beneficial effect on growth;[11,12,16] however, data were limited.[11] WHO has acknowledged the need for further research into the functional outcomes of iron supplementation programs.[7]

Risks of iron supplementation

Whilst the evidence for functional benefits of iron supplementation is scarce, there has been emerging evidence of potential harm, particularly regarding increased malaria risk in endemic areas. The seminal Pemba RCT in Tanzania was stopped early due to increased death, hospitalisation and malaria in iron replete children receiving iron compared with placebo and called for revisions of universal iron supplementation recommendations.[17] In another RCT in Tanzania, multi-nutrient supplementation (including iron) increased the number of malaria

For peer review only

For peer review only - http://bmjopen.bmj.com/site/about/guidelines.xhtml

cases by 41% in iron-deficient children.[18] However, a 2016 Cochrane review found iron supplementation safe in areas with adequate malaria prevention and management strategies.[19] In contrast, there was an increased risk in areas where this was not the case.[19] The current recommendation is for universal iron supplementation in malaria-endemic regions with a high prevalence of anaemia only "in conjunction with public health measures to prevent, diagnose and treat malaria."[7]

Iron deficiency protects against malaria

There is evidence to suggest that iron deficiency is protective against malaria. A study of preschool children in Malawi found that iron-deficient children were at lower risk of clinical malaria and parasitaemia than iron-replete children.[20] *In vitro* studies have also demonstrated that *Plasmodium falciparum* parasites are less effective at invading iron deficient red cells and prefer invading reticulocytes (which are increased in the recovery phase of iron deficiency anaemia when iron supplementation is given).[21] Parasite invasion and growth were reduced in laboratory growth and invasion studies of *P. falciparum* using red cells from anaemic children.[22] The protective effect imparted by iron deficiency in both in vitro studies was reversed when iron supplementation was given.[21,22]

Anaemia in malaria-endemic areas is less iron-responsive

Malaria is a significant cause of severe anaemia, hospitalisation, and death in endemic areas.[23–25] Several aetiologies, including malaria, were identified in a study of Malawian children with severe anaemia. However, iron deficiency was not a common contributor.[23] Similarly, in a study in children from The Gambia and Tanzania,[26] 61.2% of children were anaemic, but only 13% had iron deficiency anaemia.[26] In keeping with this, the anaemia in these areas is less iron-responsive.[11,27] Without clear evidence of the neurodevelopmental

For peer review only - http://bmjopen.bmj.com/site/about/guidelines.xhtml

benefits of iron supplementation, iron interventions are likely to be extremely expensive public health interventions in terms of cost per DALY averted, especially in sub-Saharan Africa.[28]

Malaria may be a risk factor for impaired cognitive development

Despite few studies, the majority with relatively small sample sizes and varying methods of assessing cognitive function, evidence suggests that severe and uncomplicated malaria may be independent risk factors for cognitive impairment.[29] In addition, intermittent preventive therapy in preschool and school-aged children, has improved cognitive performance.[30,31]

Intermittent preventive treatment to decrease malaria and its sequelae

Intermittent Preventive Treatment in Children (IPTc) involves the empiric treatment of malaria at regular intervals and is a potential strategy to decrease malaria rates and sequelae. A Cochrane review that included seven RCTs evaluated the effect of chemoprophylaxis and intermittent treatment for malaria in preschool children in areas with seasonal malaria and showed a reduction in clinical malaria and moderate to severe anaemia.[32] Furthermore, in a meta-analysis, iron supplementation reduced the rate of anaemia by 30% in malaria-endemic areas, compared with 60% in non-endemic regions.[11]

DP as chemoprevention

Recommended treatment for *P. falciparum* malaria is with artemisinin-based combination therapy (ACT). DP is an ACT and is among WHO's five recommended first-line treatments.[33] DP with its long half-life, given monthly, is an attractive option for IPTc. In an RCT in children in Uganda using this regimen, DP reduced clinical malaria by 58% (compared with 28% for trimethoprim-sulfamethoxazole and 7% for sulfadoxine-pyrimethamine) and parasitaemia by

For peer review only - http://bmjopen.bmj.com/site/about/guidelines.xhtml

76%. IPTc with DP also reduced moderate-to-severe anaemia by 47%.[34] The administration of DP-IPTc during MNPs/iron supplementation may improve the safety of these interventions. However, there are concerns that malaria prevention may delay the development of immunity and increase malaria risk once IPTc is ceased.[32]

Iron deficiency and malaria co-exist in Malawi

Despite important gains made worldwide in malaria control since 2010, worldwide cases increased in 2016. In Malawi, there were a total of 5.2 million presumed and confirmed malaria cases in 2016, 231,970 more cases than the year prior, resulting from a gradual increase in cases since 2014.[35] In addition, the prevalence of malaria parasitaemia in children under five years increased from 28% in 2012 to 33% in 2014. These increases come despite increases in insecticide-treated bed net coverage.[36]

Both malaria and iron deficiency anaemia remain important causes of morbidity and mortality in Malawi. In Malawian children under five years of age, malaria is the leading cause of death,[37] and the prevalence of anaemia is 63%.[38] Addressing both of these conditions is challenging. Current WHO recommendations for improving anaemia in malaria-endemic regions are difficult to interpret and implement in countries like Malawi, where malaria preventive measures and treatment are present but by no means universal.

Why this trial is needed

Several fundamental questions concerning the benefits and safety of iron-intervention programs in malaria-endemic regions remain unanswered. The IRMA trial will assess functional health outcomes from iron supplementation and identify the ideal mode of iron

For peer review only

For peer review only - http://bmjopen.bmj.com/site/about/guidelines.xhtml

delivery (MNPs versus iron syrup/drops). It will also allow evaluation of the effect of malaria chemoprevention alone on anaemia and child development.

OBJECTIVES

The primary objective is to determine a) if the efficacy of iron supplements or MNPs (containing iron) given with malaria chemoprevention is superior to malaria chemoprevention alone, or b) if the efficacy of malaria chemoprevention alone is superior to placebo, on child cognitive development (measured using locally adapted Bayley Scales of Infant and Toddler Development 3rd Edition (Bayley-III) Cognitive Composite Scores[39] at the end of the 6-month intervention period.

The specific objectives are:

1. Determine if the efficacy of a) iron (as supplements or MNPs) given with malaria chemoprevention is superior to malaria chemoprevention alone or b) if the efficacy of malaria chemoprevention alone is superior to placebo on;
    i. child cognitive development, child motor, language, behavioural development, temperament, and physical growth at the end of the six months intervention and a further six months post-intervention;
    ii. anaemia (and haemoglobin concentration), iron deficiency (and ferritin) and iron deficiency anaemia after six months of intervention and six months post intervention.
2. Compare the effects of iron given as supplements to MNPs on the above outcomes after six months of intervention and a further six-months post-intervention.

For peer review only - http://bmjopen.bmj.com/site/about/guidelines.xhtml

For peer review only

3. Determine the safety of iron (as supplements or MNPs) given with malaria chemoprevention on the risk of malaria and other morbidities versus malaria chemoprevention alone or placebo during and post-intervention.
4. Determine the efficacy of malaria chemoprevention alone compared to placebo on clinical malaria, parasitaemia and malaria-specific clinic visits, and all-cause sick visits during and after intervention.
5. Determine the safety of iron (as supplements or MNPs) given with malaria chemoprevention compared to placebo on infection risk in young children, during and post-intervention.

Study design

IRMA is a four-arm parallel-group, double-blinded, placebo-controlled, triple-dummy, individually randomised trial comparing: (a) No intervention, (b) Malaria chemoprevention only, (c) MNPs + Malaria chemoprevention and (d) Iron syrup + Malaria chemoprevention at the end of the six months intervention and at a further 6-months post-intervention. The trial design is summarised in **Figure 1**.

Study settings and participants

The trial is set in Chikwawa district, in Southern Malawi, a rural area where >70% of children 6-12 months are anaemic,[40] and where 33% of children under five years old have *Plasmodium falciparum* parasitaemia.[41] Four health facilities within the district are hosting the trial, namely: Chikwawa District Hospital, Mapelera Health Centre, Mfera Health Centre and Makhwira Health Centre. These are the primary health facilities where children attend routine under-five clinics and sick visits. Our target participants are all children aged six months residing in these health facilities' catchment area.

For peer review only

Eligibility criteria

The criteria to select participants considers participants' safety, the generalizability to national programs, and the feasibility of the trial. Children are randomly assigned to a study intervention group only if they meet all the inclusion and none of the exclusion criteria.

Inclusion criteria

1. Infant aged six months (+/- 14 days) at randomisation.
2. Availability of a legally acceptable guardian/representative and providing written consent on the participant's behalf.

Exclusion criteria

1. Severe anaemia (Hb<7g/dL).
2. Clinical signs of current infective illness (respiratory infection, diarrhoea, malaria); however, children will be screened after recovery and recruited if they meet the age eligibility criteria at the second screening.
3. Severe malnutrition (weight-for-length z-score<-3) or MUAC < 11.5 cm.
4. Established haemoglobinopathy diagnosis (e.g., sickle cell disease, beta thalassaemia major, HbE-beta thalassaemia).
5. Known congenital anomaly, developmental disorder, or severe developmental delay.
6. Children with physical or behavioural problems that will make it impossible to undertake trial assessments.
7. Children of multiple births, e.g., twins.
8. Children currently consuming iron supplements or MNPs, e.g., from the National MNPs programme.

For peer review only

For peer review only - http://bmjopen.bmj.com/site/about/guidelines.xhtml

9. Enrolment in another trial.

Trial Interventions

Participants are randomly assigned to receive one of the following interventions:

1) **No intervention (Placebo DP *plus* Placebo MNPs *plus* placebo iron syrup)**: Placebo DP (Guilin Pharmaceutical company, China)**:** three consecutive days every four weeks for six months *plus* Placebo MNPs (maltodextrin vehicle only; DSM Nutritional Products, South Africa) daily for six months *plus* placebo iron syrup (ACME Laboratories Ltd., Bangladesh) daily for six months. Malaria chemoprevention is not given routinely to children under two years of age in this setting. Thus, the placebo arm is the current standard of care in the country and no treatment is being withheld from the participants.
2) **Malaria chemoprevention only (Active DP *plus* placebo MNPs *plus* placebo iron syrup)**: Active DP (20mg dihydroartemisinin/160mg piperaquine; Guilin Pharmaceutical company, China) dosed by body weight (2/16mg/Kg) for three consecutive days every four weeks for six months *plus* placebo MNPs daily for six months, *plus* daily placebo iron syrup for six months.
3) **MNP + malaria chemopevention (Active DP *plus* active MNPs *plus* placebo iron syrup):** Active DP as above, *plus* daily active MNPs containing 10.0mg iron and 14 other micronutrients (**Supplementary Material 1**); DSM Nutritional Products, South Africa, *plus* placebo iron syrup daily for six months.
4) **Iron syrup + malaria chemoprevention (Active DP *plus* placebo MNPs *plus* active iron syrup):** Active DP as above, *plus* placebo MNPs, *plus* active iron syrup containing 10.0mg ferrous sulphate (ACME Laboratories Ltd., Bangladesh) daily for six months.

For peer review only - http://bmjopen.bmj.com/site/about/guidelines.xhtml

Randomisation, allocation concealment, and blinding

Participants are randomly allocated to one of the four study arms with 1:1:1:1 allocation via a computer-generated randomisation schedule of randomly permuted blocks stratified by sex and health facility/study sites (Chikwawa District Hospital, Mapelera health centre, Mfera health centre and Makhwira Health Centre) to achieve a balance between the intervention arms. An independent statistician from the University of Melbourne (Australia) generated the randomisation list.

An independent Trial Pharmacist pre-packed and pre-labelled participant packs containing all three investigational products based on the randomisation list. Each participating child who meets the inclusion criteria is sequentially allocated to the next available study number indicated on the randomisation list. The participant is then given a pre-labelled pre-packed Ziplock bag containing the study medication corresponding to the study number assigned to them. Hence, participants and their families and all study team members involved in collecting and analysing outcome data, including field workers, developmental assessors, laboratory scientists, statisticians, and investigators, are blinded to the treatment allocation until the database is locked and before unblinding.

Recruitment and Visits

Patient and Public Involvement

All levels of the local leadership (head men/women, sub-chiefs, chiefs, Community Health Action Groups (CHAGS), Health Surveillance Assistants (HSAs) and District Health Assembly (DHA)) are continuously engaged by the trial's Community Engagement Team (CET). CHAGs and HSAs were engaged to ensure that we reach the whole community via it's resident health "gate-keepers". Additionally, the CET conducted community sensitisation meetings to explain

For peer review only - http://bmjopen.bmj.com/site/about/guidelines.xhtml

the research objectives, what it means to participate, benefits for the individual or the community, and overall contribution to science. Finally, community members are regularly invited to contribute ideas that could help improve the success of the research thereby increasing the probability of success.

Screening and enrolment of participants

Study recruitment opened on 17 April 2020. Study activities are as detailed below (also detailed in **Supplementary Material 2**).

Pre-screening

We conducted a house-to-house enumeration (census) of all potential participants (children under six months of age) living in the target area and generated a study population database. New births are identified and reported by HSAs or registered at the health facilities' under-five clinics throughout the trial recruitment period and added to the database. Research Assistants visit all children approaching six months of age in the community. After taking written informed consent, the team reviews the child's medical history to determine potential eligibility. Eligible children are invited to visit a selected health facility for further screening.

Screening

On a pre-determined day, the child, accompanied by their parent(s) or legal guardian, visit the selected health centre. HemoCue Hb301+ (Angelholm, Sweden) is used to assess capillary Hb level to exclude severely anaemic children. Additionally, anthropometric measurements: weight and length (used to calculate weight-for-length z-scores[42]) and mid-upper arm circumference (MUAC) are performed to exclude severely malnourished children. Severely anaemic and severely malnourished children are referred to the routine health system for further investigation and treatment.

For peer review only - http://bmjopen.bmj.com/site/about/guidelines.xhtml

For peer review only

Baseline and Enrolment (at six months of age (+/- 14 days))

A second, more detailed consent form for participation in the study is signed, and a study identification number is assigned. Randomisation is performed after the following baseline evaluations (see **Supplementary Material 2**) are carried out and necessary information collected: demographic information, socioeconomic status (using the Malawi Demographic Health Survey (MDHS) standard of living scale), medical history, family care indicators[43]/index (FCI), feelings questionnaire,[44] cost survey (household out of pocket health care costs), food security questionnaire,[45] dietary diversity questionnaire,[46] developmental screening using Caregiver Reported Early Development Instrument (CREDI),[47] anthropometric measurements (weight, length, MUAC and head circumference), and anthropometric measurements for the mother (weight, height). A venous blood sample (3 mL), for haemoglobin testing, determination of iron indices (serum – for ferritin, CRP, and AGP), thick and thin malaria slides for malaria microscopy testing (to be conducted at the completion of the trial) and filter paper for plasmodium PCR, is collected from the child. The mother is given educational information regarding the use of study medications, administration of the first dose of DP and instructions on how to administer day two and day three DP treatments, administration of the first dose of iron syrup, use of MNPs and advice on future visits.

Subsequent study visits

Adherence assessment

Fortnightly 'home visits' by experienced research assistants to the participants' homes in the community are done to assess the following: 1) documentation of episodes of infection (rate, days affected), 2) recording any adverse events/effects, 3) documentation of details of

For peer review only

For peer review only - http://bmjopen.bmj.com/site/about/guidelines.xhtml

unplanned hospital and healthcare facility attendance for example to a doctor, traditional healer, clinic or hospital, 4) provision of educational information to participants on the correct use of the study interventions and encouraging adherence to study interventions, 5) adherence monitoring (measured from parental questioning along with assessment of residual MNPs and syrup) and 6) advice on future visits.

Monthly DP issuance

The research assistants issue the investigational products monthly during a home visit. The following activities (including data collection) are undertaken: issuance of the study interventions according to study protocol and adherence monitoring (measured from parental questioning along with assessment of residual MNPs and syrup). In addition, unused investigational products are collected.

Study midline visit (at 12 months of age +/- 14 days)

The participating child and their parent/ legal guardian attend the health facility at the end of the six months intervention for developmental assessments using locally adapted Bayley Scales of Infant and Toddler Development, Wolke's Behavioural Rating (WBR), and Child Temperament using the Bates' infant characteristics questionnaire. Anthropometric measurements are conducted on both the child and the mother. Venous blood (3 mL) for haemoglobin testing, determination of iron indices, malaria microscopy (to be conducted at the completion of the trial) and malaria filter paper for future PCR analyses is collected from the child.

Monthly malaria infection testing

Research assistants visit the homes of participants once per month after completion of the midline visit for assessment of episodes of infection (rate, number of days affected) in the

For peer review only - http://bmjopen.bmj.com/site/about/guidelines.xhtml
For peer review only

previous one month and documentation of unplanned hospital and/or healthcare facility attendance (details of any interim visit to doctor, clinic or hospital visits in the previous month) and record of healthcare utilisation.

Endline visit (at 18 months of age (-2 weeks or +6 weeks)

The procedures are similar to the midline visit described above and in **Supplementary Material 2.**

Unscheduled visits

Mothers or legal guardians are asked to attend the research site/clinic when their child is symptomatically unwell. They are managed according to national standard treatment guidelines by a trained healthcare provider. Blood samples for malaria rapid diagnostic test (RDT) and blood culture are taken if clinically indicated. The clinical diagnosis of the unscheduled visit is recorded in the participant's record and captured on the study's interim visit Case Report Form (CRF) and adverse event form. If the child requires inpatient care, they are referred to the Chikwawa district hospital.

For peer review only

Laboratory Procedures

A capillary blood sample is collected by finger prick from all age-eligible participants to measure the haemoglobin concentration by HemoCue (HemoCue 301+, Angelholm, Sweden) and *Plasmodium* infection by rapid diagnostic test (mRDTs); (SD Bioline Malaria Ag Pf/Pan®, Standard Diagnostics) at baseline. In addition, up to 3 mL venous blood sample are collected from all enrolled participants at baseline, midline, endline and unscheduled visits (if necessary)) for haemoglobin testing, determination of iron indices (serum – for ferritin, CRP, and AGP), thick and thin malaria slides for malaria microscopy testing and filter paper for

For peer review only - http://bmjopen.bmj.com/site/about/guidelines.xhtml

Plasmodium PCR. Families are encouraged to permit the collection of venous blood for venous haemoglobin and iron and inflammation biomarkers at all time points; however, if a family refuses venous blood collection or if collection was unsuccessful, data for the primary outcome and other non-laboratory secondary outcomes will still be collected.

Data Safety and Monitoring Committee (DSMC)

An independent DSMC comprised of international experts in clinical trials, child health, infectious diseases, epidemiology, and statistics was set up to review regularly, safety data of the ongoing trial. The DSMC may recommend to the sponsor and investigators whether to continue, modify, or terminate the trial on ethical grounds.

Outcomes

The primary outcome

Cognitive development: assessed by the differences in Bayley-III Cognitive Composite Score (CogCS) between intervention arms measured after six months intervention (midline) and 12 months post-intervention (endline). The Bayley-III is a validated index of child development that can be administered in ~0.5-1 hour and comprises cognitive, motor, and language development scales. Assessors are specially trained to administer the tests, and inter-rater reliability is established before starting data collection. In addition, we perform 10% inter-observer testing to monitor inter-rater reliability of the measure over the study period.

Secondary outcomes

Secondary outcomes include: motor and language development assessed by Bayley-III Motor (MotCS) and Language (LangCS) Composite Scores (+6m, +12m), behaviour assessed by WBR, temperament, growth assessed by weight-for-age z-score, length-for-age z-score,

For peer review only

For peer review only - http://bmjopen.bmj.com/site/about/guidelines.xhtml

weight-for-length z-score, head circumference z-score, anaemia (Hb<11g/dL), haemoglobin concentration, Iron Deficiency (Ferritin<12ng/uL adjusted for inflammation assessed by CRP or AGP), ferritin concentration, and Iron Deficiency Anaemia.

Safety will be assessed by prevalence of malaria parasitaemia and C-reactive protein (CRP) and inflammation (CRP>5mg/L) at the end of the intervention and a further 6 months post intervention; episodes of caregiver reported symptoms (e.g. fever, diarrhoea, vomiting, cough) determined by the biweekly and monthly morbidity questionnaire; episodes of unplanned clinic visits and hospitalisations and deaths.

Detection and reporting of Adverse Events and Serious Adverse Events

Non-serious Adverse Events (AEs) and serious adverse events (SAEs) are collected from the time consent is given until the participant completes the study (the final visit or withdrawal). These are identified through spontaneous reports by the participant, unplanned visits to the research site or any participating health centres, observation by the study staff, and standard questioning at each visit (biweekly during the intervention period and monthly during the postintervention period. All SAEs are reported to the sponsor and the appropriate ethics committee, whether or not they are considered causally related to the investigational products.

Sample size

The primary objective will be met if either i) MNPs+DP is superior to DP alone, or ii) Iron+DP alone is superior to DP alone or iii) DP alone is superior to placebo on the Bayley-III CogCS immediately after completion of the intervention (midline). A previous study in 2853 Bangladeshi children showed that a 0.2 standardised mean difference (SMD) in Bayley-II

For peer review only

For peer review only - http://bmjopen.bmj.com/site/about/guidelines.xhtml

438 Mental Development Index at seven months translates to a 0.7 SMD at 18 months and a 1.2
439 SMD at 64 months age.[48] Therefore, preventing a loss of at least 0.2 SMD may produce
440 magnified long-term developmental benefits as manifestations of brain dysfunction become
441 more evident. A total sample size of 2168 infants (542 per group) will provide 90% power to
442 detect an effect size of 0.25 SMD, assuming a 20% dropout and a two-sided alpha of 1.67%
443 (Bonferroni adjustment for three primary between-group comparisons).

444 **Statistical Analysis Plan**

445 A stand-alone statistical analysis plan will be detailed before breaking the blind. This plan will
446 detail all principles and precise methods of analysis, including primary and secondary
447 treatment effects of interest; alternative analyses if model assumptions do not hold; and
448 additional analyses. Participants will be analysed according to their randomised treatment
449 group for efficacy objectives and actual treatment group for safety objectives. The primary
450 outcome of Bayley-III CogCS will be analysed using a mixed-model repeated measures
451 (MMRM) analysis including in the model treatment group, time-point, and group by time-
452 point interaction. The stratification variables sex and site will be included in all models as main
453 effects. In addition, the CREDI variable (collected at baseline) will be included as a main effect
454 in the models for childhood development as it is considered prognostic for childhood
455 development outcomes. Secondary repeated continuous outcomes (e.g., growth outcomes)
456 will be analysed similarly to Bayley-III CogCS. The outcome may be appropriately transformed
457 before fitting the analysis model if considered skewed (e.g., log-transformation for ferritin).
458 Secondary repeated binary outcomes (e.g., haematologic outcomes) will be analysed using a
459 log-binomial model and account for the repeated measures with a random intercept for
460 participant. Safety (e.g., Aes, infections, hospital attendance) will be summarised during the

For peer review only - http://bmjopen.bmj.com/site/about/guidelines.xhtml

For peer review only

intervention and post-intervention periods separately and compared between the groups. Analysis of binary repeated outcome safety data (e.g., inflammation) and continuous repeated outcome safety data (e.g., CRP) will be analysed similarly to the secondary outcomes detailed above. Episode safety data (e.g., caregiver reported diarrhoea episodes) will be analysed using negative binomial regression models. Subgroup analyses (e.g., sex, site, iron deficiency) will be performed to explore the heterogeneity of the results independent of the study findings.

Data Management

Data collected from the subjects are recorded in digital Case Report Forms (CRFs) using Redcap software (Vanderbilt University, United States) on tablets. Relevant hard copy patient hospital files are scanned for reference and stored digitally, and securely. The trial is independently monitored by an external monitoring service. No interim analysis is planned.

ETHICS AND DISSEMINATION

The National Health Science Research Committee, Malawi, and the Walter and Eliza Hall Institute of Medical Research Human Research Ethics Committee, Australia, approved this study. The Malawian Pharmacy and Medicines Regulatory Authority (PMRA) also approved the trial. Additionally, the protocol is registered with the Australian and New Zealand Clinical Trials Registry (ACTRN12620000386932). Protocol modifications are reported to the ethics committees, regulatory authorities in Malawi, investigators, trial participants and trial registries. Written informed consent in the local language (see **Supplementary Material 3**) is obtained from each participant before conducting any study-related procedure. In the event where the participant is illiterate, the informed consent form is read out loudly to the potential participant in the presence of an impartial witness. The potential participant

For peer review only

For peer review only - http://bmjopen.bmj.com/site/about/guidelines.xhtml

thumbprints the consent form if they agree to participate in the study and the witness signs to indicate he/she witnessed the whole consenting process. The results will be presented and shared with the local community that hosted the research and to the international forum. We will publish in peer-reviewed scientific journals and report to relevant policymaking bodies like the Malawi Ministry of Health.

DISCUSSION

The IRMA trial will provide valuable evidence on the immediate and medium-term impacts of iron interventions (iron supplements and MNPs) and malaria prevention with DP on critical functional outcomes, most importantly, child cognitive development. These findings will complement high-quality data recently reported in South Asia to provide generalisable evidence for or against universal iron interventions across sub-Saharan Africa and other malaria-endemic settings.[10]

More than two-thirds of African children aged 6-59 months are anaemic. Preventing anaemia is considered important within the first 1000 days and aligns with WHO policy for children 6-23m of age,[7] especially because the central nervous system is rapidly developing and is highly vulnerable. The prevalence of iron deficiency in children across Africa has been shown to increase from birth to approximately one year, with a peak prevalence at 9-12 months of age, and then a decrease with increasing age. Iron interventions should thus target the critical under one year period.[49]

Recent clinical trials have indicated that the combination of Intermittent preventive treatment of malaria in infants (IPTi) and malaria vaccine (RTS<S/ AS01$_{E}$) provides much greater protection from malaria attacks in children compared to IPTi or RTS, S/AS01$_{E}$ given

For peer review only

For peer review only - http://bmjopen.bmj.com/site/about/guidelines.xhtml

alone.[50] Combining iron interventions with IPTi, would thus be optimal for preventing both malaria and anaemia in young children in this setting.

The study's strengths include the design with cognitive development as the primary functional outcome, the large sample size with the power to detect a small effect size, and the triple dummy design, which minimizes the risk of bias. The presumed benefit of cognitive development is the key rationale for iron preventive interventions in children. Limitations include routine six monthly interval malaria microscopy testing, which is not a reliable test for the prevalence of malaria parasitaemia compared to a monthly interval. However, the strong surveillance systems on clinical symptoms (biweekly home visits and phone calls) ensured that all clinical malaria cases were picked by ensuring that all children with fever and other symptoms could access hospital care and get a rapid malaria test done.

For peer review only

For peer review only - http://bmjopen.bmj.com/site/about/guidelines.xhtml

Author Contributions:

MNM drafted the initial study protocol. MNM and GM supervised the trial's implementation and wrote the protocol paper's first draft. CCN and MDK coordinated the implementation of the study. JDH, MV and LML planned for the measurement of the primary outcome. SB, RH, and ARDM wrote the statistical analysis plan. MNM and RA provided daily scientific input during the implementation of the trial. B-AB, KSP, and S-RP conceived the trial, sought funding, and provided senior scientific oversight in the conduct of the research. All authors read and approved the final manuscript.

Funding statement: This work is supported by the National Health and Medical Research Council (NHMRC), Australia, grant numbers GNT1141185 and GNT1159151. SP is funded by NHMRC GNT1158696 and GNT2009047.

Competing interests statement.

None to declare.

Acknowledgements:

We thank all the participants of the IRMA trial - including parents or legal guardians - especially during the challenging Covid-19 period. We thank the ethics and regulatory committees mentioned in the manuscript for their extensive review and approval of the study protocol and operational documents. We acknowledge the support from the Malawi government, the Chikwawa District Health Office, including the health management teams of Chikwawa District Hospital, Makhwira, Mapelera, and Mfera Health Centres. We thank the Shire River East-and-West bank communities for hosting the study and continuously bearing with the extensive study procedures, especially the conduct of the two censuses to determine

For peer review only

For peer review only - http://bmjopen.bmj.com/site/about/guidelines.xhtml

the extent of the source population. Finally, the study would not be possible without the support of the over 100 staff members (in Malawi and Australia) who contribute their time and expertise to ensure its success. We say thank you to all.

Data Statement

All relevant data are within the paper and its supporting information files.

Protocol version

IRMA Trial protocol version 4.0 dated July 25, 2022

Ethics and regulatory approvals

Ethical approval was granted by the National Health Sciences Research Committee (#19/01/2213) in Malawi and the Human Research Ethics Committee (WEHI: 19/012) in Melbourne, Australia. In addition, the Malawi Pharmacy and Medicines Regulatory Authority (PMRA) reviewed and approved the study and the purchase and importation of the investigational products to Malawi (clinical trial certificate number PMPB/CTRC/IV/06062019111).

Trial Sponsor

Training and Research Unit of Excellence (TRUE), 1 Kufa Road, Mandala. P.O. Box 30538, Chichiri, Blantyre 3, Malawi. Email: true@true.mw

Roles and responsibilities of sponsor and funder:

The sponsor and funder have no role in the study design. They will have no part or ultimate authority in the collection, management, analysis, and interpretation of data, report writing, and the decision to submit the report for publication.

For peer review only - http://bmjopen.bmj.com/site/about/guidelines.xhtml

Figure heading, caption and legend

Figure 1

Figure caption and legend: *IRMA Trial schema. Overall display of IRMA activities. MNP - Iron-containing multiple micronutrient powders; DP - Dihydroartemisinin-piperaquine; Intervention – issued monthly, from enrolment at age six months to 12 months of age. Primary endpoint – Bayley's assessment conducted at the trial midline; Cognitive assessments – Bayley Scales of Infant and Toddler Development 3rd Edition.*

For peer review only

For peer review only - http://bmjopen.bmj.com/site/about/guidelines.xhtml

References

[1] World Health Organization. The global prevalence of anaemia in 2011. Geneva, Switzerland. World Health Organization 2015. https://apps.who.int/iris/handle/10665/177094.

[2] Stevens GA, Paciorek CJ, Flores-Urrutia MC, Borghi E, Namaste S, Wirth JP, et al. National, regional, and global estimates of anaemia by severity in women and children for 2000-19: a pooled analysis of population-representative data. Lancet Glob Health 2022;10:e627–39. https://doi.org/10.1016/S2214-109X(22)00084-5.

[3] World Health Organization. Anaemia in women and children: WHO Global anaemia estimates, 2021 edition. Global estimates in women of reproductive age, by pregnancy status, and in children aged 5-59 months. Geneva, Switzerland. World Health Organization 2021. https://www.who.int/data/gho/data/themes/topics/anaemia_in_women_and_children.

[4] National Statistical Office (NSO) [Malawi], ICF. Malawi Demographic and Health Survey 2015-16,. Zomba, Malawi and Rockville, Maryland, USA. 2017.

[5] Institute for Health Metrics and Evaluation (IHME). Global Burden of Disease (GBD). 2022. https://www.healthdata.org/research-analysis/gbd.

[6] World Health Organization. WHO guideline: Use of multiple micronutrient powders for point-of-use fortification of foods consumed by infants and young children aged 6–23 months and children aged 2–12 years. Geneva, Switzerland. World Health Organization 2016:Licence: CC BY-NC-SA 3.0 IGO.

[7] World Health Organization. Guideline: Daily iron supplementation in infants and children. Geneva, Switzerland. World Health Organization 2016:b. https://www.who.int/publications/i/item/9789241549523.

[8] World Health Organization. Iron deficiency anaemia: assessment, prevention and control. A guide for programme managers. Geneva, Switzerland. World Health Organization 2001. https://www.who.int/publications/m/item/iron-children-6to23--archived-iron-deficiency-anaemia-assessment-prevention-and-control.

[9] UNICEF. Multiple micronutrient powder (MNP) supply and market update - July 2021. New York. UNICEF 2021. https://www.unicef.org/supply/reports/multiple-micronutrient-powder-mnp-supply-and-market-outlook.

[10] Pasricha S-R, Hasan MI, Braat S, Larson LM, Tipu SMM-U, Hossain SJ, et al. Benefits and Risks of Iron Interventions in Infants in Rural Bangladesh. N Engl J Med 2021;385:982–95. https://doi.org/10.1056/NEJMOA2034187.

[11] Pasricha SR, Hayes E, Kalumba K, Biggs BA. Effect of daily iron supplementation on health in children aged 4-23 months: a systematic review and meta-analysis of randomised controlled trials. Lancet Glob Health 2013;1. https://doi.org/10.1016/S2214-109X(13)70046-9.

[12] De-Regil LM, Suchdev PS, Vist GE, Walleser S, Peña-Rosas JP. Home fortification of foods with multiple micronutrient powders for health and nutrition in children under two years of age. Cochrane Database Syst Rev 2011;2011. https://doi.org/10.1002/14651858.CD008959.PUB2.

[13] Akman M, Cebeci D, Okur V, Angin H, Abali O, Akman A. The effects of iron deficiency on infants' developmental test performance. Acta Paediatr 2004;93:1391–6. https://doi.org/10.1111/J.1651-2227.2004.TB02941.X.

For peer review only - http://bmjopen.bmj.com/site/about/guidelines.xhtml

[14] Idjradinata P, Pollitt E. Reversal of developmental delays in iron-deficient anaemic infants treated with iron. Lancet 1993;341:1–4. https://doi.org/10.1016/0140-6736(93)92477-B.

[15] Adu-Afarwuah S, Lartey A, Brown KH, Zlotkin S, Briend A, Dewey KG. Home fortification of complementary foods with micronutrient supplements is well accepted and has positive effects on infant iron status in Ghana. Am J Clin Nutr 2008;87:929–38. https://doi.org/10.1093/AJCN/87.4.929.

[16] Somassè YE, Dramaix M, Traoré B, Ngabonziza I, Touré O, Konaté M, et al. The WHO recommendation of home fortification of foods with multiple-micronutrient powders in children under 2 years of age and its effectiveness on anaemia and weight: a pragmatic cluster-randomized controlled trial. Public Health Nutr 2018;21:1350–8. https://doi.org/10.1017/S1368980017003858.

[17] Sazawal S, Black RE, Ramsan M, Chwaya HM, Stoltzfus RJ, Dutta A, et al. Effects of routine prophylactic supplementation with iron and folic acid on admission to hospital and mortality in preschool children in a high malaria transmission setting: community-based, randomised, placebo-controlled trial. Lancet 2006;367:133–43. https://doi.org/10.1016/S0140-6736(06)67962-2.

[18] Veenemans J, Milligan P, Prentice AM, Schouten LRA, Inja N, van der Heijden AC, et al. Effect of supplementation with zinc and other micronutrients on malaria in Tanzanian children: a randomised trial. PLoS Med 2011;8. https://doi.org/10.1371/JOURNAL.PMED.1001125.

[19] Neuberger A, Okebe J, Yahav D, Paul M. Oral iron supplements for children in malaria-endemic areas. Cochrane Database Syst Rev 2016;2016. https://doi.org/10.1002/14651858.CD006589.PUB4.

[20] Jonker FAM, Calis JCJ, van Hensbroek MB, Phiri K, Geskus RB, Brabin BJ, et al. Iron status predicts malaria risk in Malawian preschool children. PLoS One 2012;7. https://doi.org/10.1371/JOURNAL.PONE.0042670.

[21] Clark MA, Goheen MM, Fulford A, Prentice AM, Elnagheeb MA, Patel J, et al. Host iron status and iron supplementation mediate susceptibility to erythrocytic stage Plasmodium falciparum. Nat Commun 2014;5. https://doi.org/10.1038/NCOMMS5446.

[22] Goheen MM, Wegmüller R, Bah A, Darboe B, Danso E, Affara M, et al. Anemia Offers Stronger Protection Than Sickle Cell Trait Against the Erythrocytic Stage of Falciparum Malaria and This Protection Is Reversed by Iron Supplementation. EBioMedicine 2016;14:123. https://doi.org/10.1016/J.EBIOM.2016.11.011.

[23] Calis JCJ, Phiri KS, Faragher EB, Brabin BJ, Bates I, Cuevas LE, et al. Severe anemia in Malawian children. Malawi Medical Journal 2016;28:99–107. https://doi.org/10.1056/NEJMoa072727.

[24] Newton CRJC, Warn PA, Winstanley PA, Peshu N, Snow RW, Pasvol G, et al. Severe anaemia in children living in a malaria endemic area of Kenya. Trop Med Int Health 1997;2:165–78. https://doi.org/10.1046/J.1365-3156.1997.D01-238.X.

[25] Njua-Yafi C, Achidi EA, Anchang-Kimbi JK, Apinjoh TO, Mugri RN, Chi HF, et al. Malaria, helminths, co-infection and anaemia in a cohort of children from Mutengene, south western Cameroon. Malar J 2016;15:69. https://doi.org/10.1186/S12936-016-1111-2.

[26] Pasricha SR, Atkinson SH, Armitage AE, Khandwala S, Veenemans J, Cox SE, et al. Expression of the iron hormone hepcidin distinguishes different types of anemia in

For peer review only

For peer review only - http://bmjopen.bmj.com/site/about/guidelines.xhtml

African children. Sci Transl Med 2014;6. https://doi.org/10.1126/SCITRANSLMED.3008249.
[27] Zlotkin S, Newton S, Aimone AM, Azindow I, Amenga-Etego S, Tchum K, et al. Effect of iron fortification on malaria incidence in infants and young children in Ghana: a randomized trial. JAMA 2013;310:938–47. https://doi.org/10.1001/JAMA.2013.277129.
[28] Pasricha SR, Gheorghe A, Sakr-Ashour F, Arcot A, Neufeld L, Murray-Kolb LE, et al. Net benefit and cost-effectiveness of universal iron-containing multiple micronutrient powders for young children in 78 countries: a microsimulation study. Lancet Glob Health 2020;8:e1071–80. https://doi.org/10.1016/S2214-109X(20)30240-0.
[29] Fernando SD, Rodrigo C, Rajapakse S. The "hidden" burden of malaria: cognitive impairment following infection. Malar J 2010;9. https://doi.org/10.1186/1475-2875-9-366.
[30] Clarke SE, Jukes MC, Njagi JK, Khasakhala L, Cundill B, Otido J, et al. Effect of intermittent preventive treatment of malaria on health and education in schoolchildren: a cluster-randomised, double-blind, placebo-controlled trial. Lancet 2008;372:127–38. https://doi.org/10.1016/S0140-6736(08)61034-X.
[31] Jukes MCH, Pinder M, Grigorenko EL, Smith HB, Walraven G, Bariau EM, et al. Long-Term Impact of Malaria Chemoprophylaxis on Cognitive Abilities and Educational Attainment: Follow-Up of a Controlled Trial. PLoS Clin Trials 2006;1:e19. https://doi.org/10.1371/JOURNAL.PCTR.0010019.
[32] Meremikwu MM, Donegan S, Sinclair D, Esu E, Oringanje C. Intermittent preventive treatment for malaria in children living in areas with seasonal transmission. Cochrane Database Syst Rev 2012;2012. https://doi.org/10.1002/14651858.CD003756.PUB4.
[33] World Health Organization. Guidelines for the treatment of malaria. World Health Organization 2015. https://apps.who.int/iris/handle/10665/162441.
[34] Bigira V, Kapisi J, Clark TD, Kinara S, Mwangwa F, Muhindo MK, et al. Protective efficacy and safety of three antimalarial regimens for the prevention of malaria in young Ugandan children: a randomized controlled trial. PLoS Med 2014;11. https://doi.org/10.1371/JOURNAL.PMED.1001689.
[35] World Health Organization. World malaria report 2017. Geneva, Switzerland. World Malaria Report 2017 2017:196.
[36] Zgambo M, Mbakaya BC, Kalembo FW. Prevalence and factors associated with malaria parasitaemia in children under the age of five years in Malawi: A comparison study of the 2012 and 2014 Malaria Indicator Surveys (MISs). PLoS One 2017;12. https://doi.org/10.1371/JOURNAL.PONE.0175537.
[37] World Health Organization. Malawi statistical profile. World Health Organization 2015. https://www.afro.who.int/countries/malawi.
[38] Whitson DT. Farta Child Survival Project Final Evaluation – CARE. 2007. https://careevaluations.org/evaluation/farta-final-evaluation/.
[39] Bayley N. Bayley Scales of Infant and Toddler Development, Third Edition (Bayley--III®) [Database record]. APA PsycTests 2015. https://psycnet.apa.org/doiLanding?doi=10.1037%2Ft14978-000.
[40] National Statistical Office ZM, ICF Macro CMU. 2010 Malawi Demographic and Health Survey (2010 MDHS) 2011. http://www.nsomalawi.mw/index.php?option=com_content&view=article&id=175&Itemid=67.

For peer review only - http://bmjopen.bmj.com/site/about/guidelines.xhtml

[41] National Malaria Control Programme - NMCP/Malawi, ICF International. Malawi Malaria Indicator Survey - MIS - 20142014. Lilongwe, Malawi: NMCP/Malawi and ICF International. 2015.

[42] WHO Multicentre Growth Reference Study Group. Assessment of differences in linear growth among populations in the WHO Multicentre Growth Reference Study. Acta Paediatr Suppl 2006;450:56–65. https://doi.org/10.1111/J.1651-2227.2006.TB02376.X.

[43] Hamadani JD, Tofail F, Hilaly A, Huda SN, Engle P, Grantham-McGregor SM. Use of Family Care Indicators and Their Relationship with Child Development in Bangladesh. J Health Popul Nutr 2010;28:23. https://doi.org/10.3329/JHPN.V28I1.4520.

[44] Radloff LS. The CES-D Scale: A Self-Report Depression Scale for Research in the General Population n.d.

[45] Coates J, Swindale A, Bilinsky P. Household Food Insecurity Access Scale (HFIAS) for Measurement of Food Access: Indicator Guide: Version 3. Washington, DC: FHI 360/FANTA 2007.

[46] Swindale Anne, Paula Bilinsky. Household Dietary Diversity Score (HDDS) for Measurement of Household Food Access: Indicator Guide. Washington, DC: FHI 360/FANTA 2006. https://www.fantaproject.org/monitoring-and-evaluation/household-dietary-diversity-score.

[47] McCoy DC, Sudfeld CR, Bellinger DC, Muhihi A, Ashery G, Weary TE, et al. Development and validation of an early childhood development scale for use in low-resourced settings. Popul Health Metr 2017;15. https://doi.org/10.1186/S12963-017-0122-8.

[48] Hamadani JD, Tofail F, Huda SN, Alam DS, Ridout DA, Attanasio O, et al. Cognitive Deficit and Poverty in the First 5 Years of Childhood in Bangladesh. Pediatrics 2014;134:e1001–8. https://doi.org/10.1542/PEDS.2014-0694.

[49] Muriuki JM, Mentzer AJ, Webb EL, Morovat A, Kimita W, Ndungu FM, et al. Estimating the burden of iron deficiency among African children. BMC Med 2020;18:31–31. https://doi.org/10.1186/S12916-020-1502-7.

[50] Greenwood B, Cairns M, Chaponda M, Chico RM, Dicko A, Ouedraogo JB, et al. Combining malaria vaccination with chemoprevention: a promising new approach to malaria control. Malar J 2021;20:1–7. https://doi.org/10.1186/S12936-021-03888-8/METRICS.

For peer review only

For peer review only - http://bmjopen.bmj.com/site/about/guidelines.xhtml

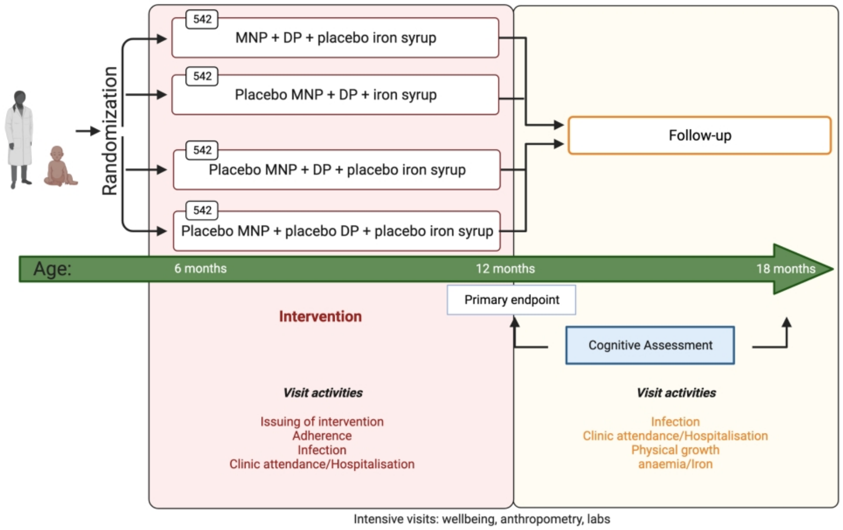

Figure 1: IRMA study design

645x516mm (118 x 118 DPI)

Supplementary material 1: Multiple micronutrient powders (MNPs) contents

	**Micronutrient name**	**Quantity**
1.	Niacinamide	7.02 - 8.58 mg/g
2.	Riboflavin	0.653 - 0.798 mg/g
3.	Pyridoxine	0.585 – 0.715 mg/g
4.	Thiamine	0.675 – 0.825 mg/g
5.	Vitamin C	32.4 – 39.6 mg/g
6.	Vitamin A	522 – 667 µgRE/g
7.	Vitamin E (as Tocopherol Eq.)	5.04 – 6.16 mgTE/g
8.	Vitamin D3	6.75 – 8.63 µgD/g
9.	Vitamin B12	1.09 – 1.40 µg/g
10.	Folic Acid	109 – 134 µg/g
11.	Selenium	17.0 – 24.5 µg/g
12.	Iodine	9.00 -129.6 µg/g
13.	Iron	10.00 -12.10 mg/g
14.	Zinc	4.10 – 0.739 mg/g
15.	Copper	0.605 – 0.739 mg/g

For peer review only

Supplementary material 2: Main study activities per visit

		Intervention period (Active follow-up)													Post intervention period (Passive follow up)					
	**Month**	**M1**			**M2**		**M3**		**M4**		**M5**		**M6**		**M7**	**M8**	**M9**	**M10**	**M11**	**M12[‡]**
**Planned activity**	**Day** 0	D 1[#]	D 14	D 28	D 42	D 56	D 70	D 84	D 98	D 112	D 126	D 140	D 154	D 168[§]						
**Location**	**H/HF**	**HF**	**H**	**H**	**H**	**H**	**H**	**H**	**H**	**H**	**H**	**H**	**H**	**HF**	**H**	**H**	**H**	**H**	**H**	**HF**
Pre-screening/ screening, & informed consent	**X**	**X**																		
Randomization		**X**																		
Demographic, clinical, CREDI and SES assessments		**X**																		
Family Care Index (FCI)		**X**												**X**						**X**
Bayley-III Cognitive Composite Score (CogCS)														**X**						**X**
Bayley-III Motor (MotCS) and Language (LangCS) Composite Scores														**X**						**X**
Behaviour: Wolke's Behaviour Ratings (WBR)														**X**						**X**
Food security & dietary diversity questionnaires		**X**												**X**						**X**
Anthropometry[1] - (mother and child)		**X**												**X**						**X**
**Laboratory procedures**																				
Haemoglobin		**X**												**X**						**X**
Malaria microscopy		**X**												**X**						**X**

For peer review only

Serum iron marker tests[2]		X												X						X
Serum for inflammatory marker tests (CRP/AGP)[3]		X												X						X
**Active follow-up:**																				
Compliance assessment		X	X	X	X	X	X	X	X	X	X	X	X	X						
MNPs/Placebo MNPs[4](distribute, instruct)		X		X		X		X		X		X								
DP/Placebo DP[5] (distribute, instruct & observe use/intake)		X		X		X		X		X		X								
Iron syrup/Placebo iron syrup[6]		X		X		X		X		X		X								
Provide educational information		X	X	X	X	X	X	X	X	X	X	X	X	X						
Assess infections[7]			X	X	X	X	X	X	X	X	X	X	X	X	X	X	X	X	X	X
Adverse Events monitoring			X	X	X	X	X	X	X	X	X	X	X	X	X	X	X	X	X	X
Health care utilization monitoring		X	X	X	X	X	X	X	X	X	X	X	X	X	X	X	X	X	X	X

# - Baseline; § - Midline visit at day 168. Primary outcome was assessed at day 168 ± 14 days as per protocol)**; ‡ - Endline; M –** Month; **D –** Day; **H – Home** (visit at the Participant's Home)**; HF –** Visit at the Health Facility; **X** – scheduled activity. **CREDI -** Caregiver Reported Early Development Instrument**; SES -** Social Economic Status; [1]**Anthropometric assessments** (mother and child): height/length, weight; [2]**Serum iron marker tests**: Serum ferritin; [3]**Inflammation markers**: CRP (C-Reactive Protein) or AGP (alpha 1 acid glycoprotein); [4]**MNPs** containing 10.0mg iron and 14 other micronutrients, daily for 6 months; **Placebo MNPs**: (maltodextrin), daily for 6 months; [5]**DP/Placebo DP** (20mg dihydroartemisinin/ 160mg piperaquine in suspension) dosed by body weight, three consecutive days every 4 weeks for 6 months; [6]**Iron syrup/Placebo iron syrup:** 10mg daily for six months; [7]**Infections**(assessment): (rate and number of days affected) fever/respiratory/diarrhoea.

NB: Families were encouraged to permit collection of venous blood for venous haemoglobin and iron and inflammation biomarkers at all timepoints; however, if a family refused venous blood collection or if collection was unsuccessful, data for the primary outcome and other non-laboratory secondary outcomes was still collected.

For peer review only

IRMA Study

Training and Research Unit of Excellence (TRUE), Department of Public Health, College of Medicine, Blantyre, Malawi

University of Melbourne, Victoria, Australia

1. INFORMED CONSENT – SCREENING

Protocol No. 2213	Version No 4.0	Date: July 25, 2022

Protocol Title: Benefits and safety of IRon supplementation with MAlaria chemoprevention to children in Malawi (IRMA) - A randomised controlled trial

Investigator's name: Prof Kamija Phiri and Dr Sant-Rayn Pasricha

Organization: Training and Research Unit of Excellence (TRUE), Department of Public Health, College of Medicine, Blantyre, Malawi in collaboration with The Walter and Eliza Hall Institute, Melbourne, Australia

Purpose of the research: We intend to assess if a program of iron supplementation or Multiple Micronutrient Powder (MNP), with antimalarials, have an impact on child development, both immediately following intervention and after 6 months further follow up.

Background:
As you may know, anaemia and malaria are a major public health problem among pre-school children in Malawi. However, we do not yet know whether preventing or treating anaemia by giving iron supplements or Micronutrients to all children benefits child health. In this study we want to see the effect of malaria prevention and iron interventions on children's cognitive capacity both in short and long run. Our main objective of this consent form is to check if your child is eligible to participate in the study.

Why are you invited to participate in the study?
We are inviting your child to participate in this study, because your child is within the age of 6 months +/-14 days and you are living in the study area in Chikwawa.

Methods and procedures
If you agree, we will measure your child's haemoglobin level by taking one or two drops of blood from her/his finger tip. If your child Hb level is ≥7.0g/dL, and there are no other reasons for ineligibility, we will administer our screening questionnaire. We will then invite your child to participate in our study. You will be requested to come to a local test centre in a scheduled date to enrol in the study and sign a separate consent form for study's procedure. If your child's Hb level found to be <7.0g/dl, we will refer him/her to the local health centre for appropriate management.

Risk and benefits
There is no risk involved participating in this screening procedure. Blood sample will be collected from fingertip following all aseptic precautions by an expert technician. Only minimum momentary pricking pain might be experienced by your child. However,

For peer review only - http://bmjopen.bmj.com/site/about/guidelines.xhtml

our expert technician will do the job with much care and precautions. We will inform you of your child's haemoglobin level and provide necessary advice if your child is found severely anaemic.

Privacy, anonymity and confidentiality
All information collected will remain strictly confidential

Future use of information
The information collected from you will help us decide whether your child is eligible to participate in the study. It will not be used in future.

Right not to participate and withdraw
Your participation is completely voluntary. You have the right to refuse to participate in this screening process or to refuse to participate in the main trial or to withdraw from the study at any time. Your decision will not affect getting any services from the health facilities.

Principle of compensation
There is no monetary compensation for your participation.

Answering your questions/ Contact persons
We would be obliged to provide you information related to the condition(s) of your child and would be happy to answer your questions about the study. You would be able to freely communicate with any investigator of this study (contact address provided below).

You can ask any questions you have about the study or about your rights. If you have other questions later, you can contact our study teams on the following numbers:

Chikwawa Boma: 0995 835 215 or 0883 536 917

Mfera: 0990 676 012 or 0881 861 069

Mapelere: 0990 676 014 or 0881 861 169

Makwhira: 0994 952 510 or 0883 536 925

If you want to know more about your rights as a research participant you can contact the NHSRC Director, at the Ministry of Health on the following details, Phone: 0999 397 913. Email: cmitambo@gmail.com or mohdoccentre@gmail.com

If you agree to our proposal of enrolling you and your child in our study, please indicate that by putting your signature or your left thumb impression at the specified space below.

Thank you for your cooperation

For peer review only - http://bmjopen.bmj.com/site/about/guidelines.xhtml

IRMA Study

Name of Parent/ Guardian

Signature or right thumb impression of Parent/ Guardian/ Attendant | Date

Name of witness (*If Parent/ Guardian cannot read and write*)

Signature of witness | Date

Name of Investigator or study staff

Signature of Investigator or study staff | Date

For peer review only

For peer review only - http://bmjopen.bmj.com/site/about/guidelines.xhtml

IRMA Study

Training and Research Unit of Excellence (TRUE), Department of Public Health, College of Medicine, Blantyre, Malawi

University of Melbourne, Victoria, Australia

1. Kalata Yopempha Chilolezo

Protocol No. 2213	Version No. 4.0	Date: July 25, 2022

Mutu wa kafukufuku: kufufuza ubwino ndi chitetezo cha mankhwala okhala ndi ironi komanso mankhwala oteteza ku malungo kwa ana muno m'Malawi

Oyang'anira kafukufuku: Prof Kamija Phiri ndi Dr Sant-Rayn Pasricha

Bungwe: Training and Research Unit of Excellence (TRUE), Department of Public Health, College of Medicine, Blantyre, Malawi in collaboration with University of Melbourne, Victoria, Australia

Cholinga cha kafukufuku

Tikuchita kafukufuku ndi cholinga chokuti tifufuze ngati mankhwala okhala ndi ayironi angathandize pa kakulidwe ka mwana potsatira kupereka mankhwalawa kwa miyezi isanu ndi umodzi motsatizana ndi kulondoloza kwa miyezi ina isanu ndi umodzi yowonjezera.

Zokhudzana ndi kafukufukuyi

Monga mukudziwa kuchepa kapena kusowa kwa magazi ndi vuto lalikulu makamaka kwa ana achichepere amene sanayambe sukulu kuno ku Malawi. Ngakhale zili chonchi, sitikudziwa ngati kuteteza mwana ku vuto limenelo losowa magazi popereka mankhwala okhala ndi ayironi kungathandize umoyo wa mwanayu. Mu kafukufukuyi tikufuna tiwone zotsatira zopereka mankhwalawa kwa ana makamaka pa kakulidwe kawo mu kaganizidwe ndi makhalidwe. Pakadali pano cholinga chathu pakucheza kwathu ndi inu chenicheni ndi chakuti tione ngati mwana wanu angakhale oyenera kutenga nawo mbali mu kafukufukuyu.

Kodi nchifukwa chiyani tasankha mwana wanu kuti atenge nawo mbali mukafukufuku?

Takuitanani kuti mutenge nawo mbali mukafukufukuyi makamaka polingana ndi kuti mwana wanu ali ndi miyezi isanu ndi theka kapena sanaposere miyezi isanu ndi umodzi kuonjezerapo theka, komanso mukukhala dera lino la Chikwawa lomwe tikufuna tichitiremo kafukufuku ameneyu.

Zochitika zokhudzana ndi kafukufukuyi.

Ngati mwagwirizana nazo kuti mwana wanu atenge nawo mbali, tiyeza kuchuluka kwa magazi a mwana wanu potenga dontho la magazi kuchokera pa chala chake. Ngati taona kuti magazi ake ndiokwanira ndipo kuti palibenso choletsa chilichonse, tikupemphani kuti tikufunseni mafunso angapo. Kenako tidzakupemphani kuti mwana wanu atenge nawo mbali mukafukufukuyi. Mudzafunsidwa kuti mudzabwere ku

For peer review only - http://bmjopen.bmj.com/site/about/guidelines.xhtml

IRMA Study

chipatala pa tsiku lomwe tidzakuwuzani kuti mwana wanu adzalowe nawo mukafukufukuyi ndipo kuti mudzasayinanso pepala lolowera mukafukufukuyi. Ngati magazi a mwana wanu ali ochepa tidzakutumizani kuchipatala kuti akamuthandize moyenelera.

Ubwino ndi zoopsya za kafukufuku
Palibe zoopsya zilizonse pa kufufuza ngati mwana wanu angakhale oyenera kutenga nawo mbali mukafukufukuyi. Dontho la magazi lidzatengedwa pa chala cha mwana wanu ndipo adzatenge dontho la magazili ndi adokotala odziwa bwino ntchito yawo. Mwana wanu adzamva kupweteka pa kanthawi komwe adzatengedwa magaziwo basi. Koma adokotalawo adzawonetsetsa kuti agwira ntchito yawo mwaukadaulo komanso mopanda kupweteka mwana wanu. Tidzakuwuzani zotsatira za kuchuluka kwa magazi a mwana wanu ndipo tikuwuzaninso zomwe mungachite ngati titamupeza kuti mwana wanu ali ndi magazi osakwanira.

Chitetezo cha zokhuza mwana wanu
Zonse zimene mutiuze komanso zotsatira za zimene tiyeze kwa mwana wanu palibe uyo adzadziwe ndipo ofufuza adzaonetsetsa kuti zatetezedwa koposa moti palibenso amene angazitenge komanso angadzadziwe.

Tsogolo la zinthu zomwe tiyese komanso zomwe mutiuze
Zomwe mutiuze apa zitithandiza kuti tichite chiganizo ngati mwana wanu ali oyenera kutenga nawo mbali mukafukufukuyi kapena ayi. Ndipo izi sizidzagwiritsidwanso ntchito ina mtsogolomu kupatula imeneyi basi.

Ufulu otenga nawo mbali kapena kusiya kafukufukuyi
Dziwani kuti kutenga mbali mukafukufukuyi ndi kosakakamiza ayi. Muli ndi ufulu kukana kuti mwana wanu asatenge nawo mbali. Pamene mwasankha kuti mwana wanu akhale m'modzi mwa ana amene angatenge nawo mbali mukafukufukuyi, mulinso ndi ufulu kutuluka mukafukufuku pamene mwafuna. Chiganizo chanu sichisokoneza chisamaliro chomwe mwana wanu kapena inu mungafune kulandira kuchipatala china chilichonse.

Zolipira
Palibe ndalama ina iliyonse yomwe mulandire potenga nawo mbali pamene tikufufuza ngati mwana wanu ali woyenera kutenga nawo mbali mukafukufukuyi.

Ngati muli ndi mafunso komanso amene angayankhe
Ife tikuyenera kukuwuzani chilichonse chokhudzana ndi umoyo wa mwana wanu ndipo tidzakhala okondwa kuyankha funso lina lililonse lomwe mungakhale nalo lokhudzana ndi kafukufukuyi. Muli ololedwanso kuyankhula ndi omwe akuchita kafukufukuyi ngati mungakondwe kutero, manambala awo alembedwa m'musimu.

Mukhonza kufunsa funso lililonse lokhudzana ndi kafukufukuyi komanso ufulu wanu. Ngati mungakhale ndi mafunso ena mwina nthawi ina mukhonza kufunsa pa manambala awa:

Chikwawa Boma: 0995 835 215 or 0883 536 917

For peer review only - http://bmjopen.bmj.com/site/about/guidelines.xhtml

IRMA Study

Mfera: 0990 676 012 or 0881 861 069

Mapelera: 0990 676 014 or 0881 861 169

Makwhira: 0994 952 510 or 0883 536 925

Ngati mukufuna kudziwa zambiri zokhudza ufulu wanu otenga nawo mbali mukafukufukukuyi, mukhonza kuyimba foni ku bungwe lowona za kafukufuku ku unduna wa za umoyo la National Health Science Research Commitee (NHSRC) polemba kalata ku: cmitambo@gmail.com kapena mohdoccentre@gmail.com. Komanaso mukhonza kuyimba foni ku 0999 397 913.

Ngati mwagwirizana ndi kupempha kwathu kuti inu ndi mwana wanu achite nawo kafukufukuyi chonde onetsani ichi pakusayinira kapena kudinda chala chanu chachikulu chakumanzere m'musimu.

Zikomo chifukwa cha nthawi yanu

____
Dzina la kholo kapena womuyang'anira mwana

Saini la kholo kapena womuyang'anira mwana | Tsiku

Dzina la mboni (*ngati kholo kapena womuyang'anira mwana sadziwa kulemba kapena kuwerenga*)

Saini la mboni | Tsiku

Dzina la woyang'anira kafukufuku

Saini ya woyang'anira kafukufuku | Tsiku

For peer review only - http://bmjopen.bmj.com/site/about/guidelines.xhtml

Training and Research Unit of Excellence (TRUE), Department of Public Health, College of Medicine, Malawi

Walter and Eliza Hall Institute (WEHI), University of Melbourne, Victoria, Australia

2. INFORMED CONSENT FORM – ENROLMENT

Protocol No. 2213	Version No. 4.0	Date: July 25, 2022

Project title: Benefits and safety of IRon supplementation with MAlaria chemoprevention to children in Malawi (IRMA) - A randomised controlled trial

Principal Investigators: Prof Kamija Phiri and Dr Sant-Rayn Pasricha
Organization: Training and Research Unit of Excellence (TRUE), Department of Public Health, College of Medicine, Blantyre, Malawi in collaboration with Walter and Eliza Hall Institute of Medical Research, Australia

This is an invitation for the child in your care to take part in this research project. Ask questions about anything that you don't understand or want to know more about. Before deciding whether or not the child can take part, you might want to talk about it with a relative, friend or other person.

Purpose of the research
As you may know many children in Malawi and elsewhere in the world are anaemic. Many organisations including the Government of Malawi recommend giving all children iron supplementation or multiple micronutrients to prevent anaemia, and these are both currently made and available in Malawi. However, we do not yet know whether giving iron supplements or Multiple Micronutrient Powders are a good idea for improving overall child health, and which one of these is better. In this project, we are planning to see the effect of these treatments on development, growth and health of young children. We also will find out whether giving routine antimalarials to children helps their growth and development. The results of this study will help governments and health policymakers decide if all children should be given these interventions in the future.

Why you are invited to participate in the study?
We have selected Chikwawa district for this study based on available national data on anaemia. Your child lives in the selected area, and has fulfilled all the study eligibility criteria. Hence, we are inviting you to allow your child to take part in this study.

Methods and procedures
What does participation in this research involve?
You will bring your child to our local clinic 3 times; 1) at beginning (today), 2) after 6 months i.e. at the end of receiving the treatment and 3) after 12 months from the beginning of the study i.e. for final tests. At the 1st occasion, a trained tester will take details about socio-economic status of your family, type of toys and stimulation activities your child receives at home, your child's temperament and your feelings towards your life and she will also test your child's brain development by playing with

For peer review only - http://bmjopen.bmj.com/site/about/guidelines.xhtml

him/her with some toys. She will also measure yours and your child's height/length, weight, Mid Upper Arm Circumference and child's head circumference. At the end of the interview, a trained technician will collect 3 ml of venous blood (a little less than 1 teaspoon) from your child. On the following 2 clinic visits, all the measurements will be repeated except the demographic and socio-economic status questions. Together, these procedures will take about 2 to 3 hours. We will collect a stool sample from all enrolled children at the at the end of the treatments, and at the final visit.

We would also like to invite you for a long-term follow-up beyond 12 months in future, which depends on availability of funds and would like to seek your consent for preserving and using the left-over samples for future research, and in contacting you for further information in future.

Home visit
When your child is enrolled, one of our research assistants will visit your child every 2 weeks for six months (when s/he is receiving the medicines) and every month for the next 6 months. She/he will record previous weeks' dose taken, bring back the unused quantities and supply new doses for the following 2 weeks. She/he will also collect information on child's morbidity (fever/infection/any other illness), visit to a doctor or hospitalization occurred in previous 2 weeks during active intervention period.

What does the child have to do?
Your child will be participating in a double-blind randomised controlled research project. We wish to compare antimalarial with Dihydroartemisinin-Piperaquine (DP), with or without iron supplements or micronutrient powders, and placebo which is an inert substance. Your child has a one in four chance of receiving either DP, DP with iron supplements (as iron syrup), DP with multiple micronutrient powders, or placebo. However double-blind means that neither you nor the study doctors and investigators will know which of the treatments the child is receiving. After the initial measurements, we will assign your child to one of the four treatments. Your child will be provided with two formulations: a bottle of syrup, and a box of sachets containing powders. During the study, your child will be asked to take 1.3 ml of syrup and one sachet of powders every day for six months. The drops can be given with a graduated dropper or syringe. The powders should be sprinkled on your child's food once it has cooled down a little. Syrup may contain iron or placebo, and sachet may contain multiple micronutrient powder or placebo powder. 1.3 ml of Iron syrup will contain 10mg elemental iron and each MNP sachet will contain 10mg iron, as well as a complement of other nutrients. Once a month, we will also provide Dihydroartemisinin-piperaquine (DP), (or its placebo) which is given over three days. Dihydroartemisinin-piperaquine (DP), is one of the artemisinin containing combination therapies (ACTs). ACTs are now the standard for treatment of *P. falciparum* malaria in both adults and children. A placebo syrup or sachet is a medication with no active ingredients. It looks like the real thing but is not. Once your child is assigned to a group, s/he will be receiving this agent for 6 months.

If your child ever becomes sick or unwell, you can either take them to the clinic, doctor or hospital, as convenient. We will ask about any illness your child may have during our weekly visits.

Right not to participate and withdraw

For peer review only - http://bmjopen.bmj.com/site/about/guidelines.xhtml

Does the child have to take part in this research project?
Participation in this research project is voluntary. If you do not wish for the child to take part, they do not have to. If you decide that they can take part and later change your mind, you are free to withdraw the child from the project at any stage. If you do decide that the child can take part, you will be given this Participant Information and Consent Form to sign and you will be given a copy to keep. However, your child's participation in this study would be greatly valuable.

What if I withdraw the child from this research project?
If you do withdraw the child during the research project, the study doctor and relevant study staff will not collect additional personal information, although personal information already collected will be retained to ensure that the results of the research project can be measured properly. You should be aware that data collected by the sponsor up to the time of withdrawal will form part of the research project results. However, withdrawing from the study will involve no penalty and your service from the health facilities will not be withheld, nor will it hamper relationship with those treating him/her, or their relationship with the institutions (College of Medicine and Walter and Eliza Hall Institute).

What are the alternatives to participation?
If you wish, you may decline involvement in the research project and seek attention for anaemia and nutritional status from elsewhere.

Risk and benefits
What are the possible risks and disadvantages of taking part?
Iron supplements and micronutrient powders have been already given to many thousands or millions of children, and are considered safe. However, we will be looking for possible side effects. Many side effects go away shortly after treatment ends. If your child has any of these side effects, or you are worried about them, you can easily talk with us. However, sometimes side effects can be serious, long lasting or permanent. If a severe side effect or reaction occurs, we may need to stop the child's treatment. The child's researcher will discuss the best way of managing any side effects with you. Usually there is no need to stop the supplements.

Side Effect	How often is it likely to occur?	How severe might it be?	How long might it last?
Stomach aches, nausea, constipation or diarrhoea	Perhaps up to 1 in 4 children.	Mild	A few days or the duration of the treatment; but it is usually tolerable
Infection (e.g., diarrhoea)	As often as in children not receiving iron, or perhaps slightly more commonly	About as often as in children not receiving iron, or perhaps slightly more severe.	A few days.
Darkening of stools – this is not a harmful effect at all, and will not harm the child	Common	This is not severe.	For the duration of treatment.

For peer review only - http://bmjopen.bmj.com/site/about/guidelines.xhtml

During collection of blood samples slight pricking pain, swelling or bluish colour on the pricking point might be experienced but these resolve quickly. Our experienced technician will ensure complete hygienic precautions and minimum distress.

What are the possible benefits of taking part?
We cannot guarantee or promise that the child will receive any benefits from this research; however, possible benefits may include treatment of anaemia if your child received iron, with improved energy levels, strength and wellbeing in your child, improved mental development, and improved growth. We will share your child's Hb level with you. If your child is severely anaemic or suffers from significant developmental disability diagnosed during brain development tests, we will refer your child to the appropriate health care centres during the study period. Your child's involvement will help us understand how anaemia should best be treated, and so it will have an impact on how we treat children all over the world. If you are invited to come to the research facility, you will be compensated for cost of travel.

Privacy, anonymity and confidentiality
What will happen to information about the child?
By signing the consent form you consent to the study doctor and relevant research staff collecting and using personal information about the child for the research project. Any information obtained in connection with this research project or in any publication and/or presentation, information will be provided in such a way that the child cannot be identified. The data will be kept securely at Training and Research Unit of Excellence and will be shared with The University of Melbourne in Australia through a password protected file. Only researchers on this project will have access to the data. The data will be used for this project and for related projects in the future. In line with data storage requirements for studies involving children, we will retain the data for 25 years and then erase it. The child's information will only be used for the purpose of this research project and it will only be disclosed with your permission, except as required by law.

We will seek to find out the diagnosis of any visit your child makes to a health centre or hospital during the study. By signing the consent form, you agree to the study team accessing health records if they are relevant to the child's participation in this research project.

Information obtained during the research project including your child's health records if accessed as above, are subject to inspection (for the purpose of verifying the procedures and the data) authorised representatives of the Sponsor, Walter and Eliza Hall Institute of Medical Research, the College of Medicine, or as required by law. By signing the Consent Form, you authorise release of, or access to, this confidential information to the relevant study personnel and regulatory authorities as noted above.

Future use of information
What will happen to the child's test samples?
You will be asked to provide consent for the collection of the child's blood during the research project. Collection of blood is the most essential part of involvement in this project.

Samples will be analysed for iron levels in the blood, so we can see if the interventions have shown an effect. We will also store leftover samples in the College of Medicine,

For peer review only - http://bmjopen.bmj.com/site/about/guidelines.xhtml

Blantyre Campus, for 5 years from the end date of the study. Samples will be stored in such a way that identity of your child is kept confidential by use of a code. We may measure other markers in the blood samples that relate to the current project (your child's health, nutrition, growth and development) in your child's sample in the future. If samples cannot be tested in Malawi, we may send them overseas for testing, after which they will be destroyed.

Genetic testing:
We will collect samples for future testing of DNA. We will not be testing for genetic diseases, and so the results will not have implications for your child's or the rest of your family's health or diagnosing a genetic disease in the future. Instead, we anticipate testing for genetic differences which may be linked with anaemia, nutrition, development, health and growth, or infection status. Samples and results of genetic testing will not be released to anyone other than the researchers or their colleagues helping with analysis of the samples. If samples cannot be tested in Malawi, we may send them overseas for testing, after which they will be destroyed.

What if new information arises during this research project?
Sometimes during the course of a research project, new information becomes available about the treatment that is being studied. If this happens, the study doctor will tell you about it and discuss with you whether you want your child to continue in the research project. If you decide to withdraw your child from the study, the study doctor will make arrangements for their regular health care to continue. If you decide that the participant can continue in the research project, you will be asked to sign an updated consent form.

Can the child have other treatments during this research project?
Whilst the child is participating in this research project, we discourage you from giving your child other iron formulations because it may mean your child is getting a double dose, and it will make the results of the study difficult to interpret. Please tell us if your child takes or is going to take iron.

Could this research project be stopped unexpectedly?
Yes. This research project may be stopped unexpectedly for a variety of reasons such as lack of funds or unacceptable side effects.

What happens when the research project ends?
At the end of the study, we will test your child for anaemia. If your child is anaemic, we will refer your child to the local health centre for treatment. Once we complete the study and have prepared the final reports, we will provide information on the results of the project to local health workers and facilities.

Principle of compensation
Complaints and Compensation
If you have any complaints or you want to know more about your rights as a research participant you can contact the NHSRC Director at the Ministry of Health in Lilongwe, Phone: +265 999 397 913 Email: cmitambo@gmail.com or mohdoccentre@gmail.com.

For peer review only - http://bmjopen.bmj.com/site/about/guidelines.xhtml

If your child suffers any injuries or complications as a result of this research project, you should contact the study team as soon as possible and you will be assisted with arranging appropriate medical treatment.

Compensation

You will be reimbursed, in Malawi Kwacha, the equivalent of 10 USD for each scheduled health facility visit for the time taken to participate in the study, inclusive of any travel costs.

Answering your questions/ Contact persons

Who is organising and funding the research?

This research project is being conducted by Professor Kamija Phiri of the College of Medicine and Dr Sant-Rayn Pasricha of Walter and Eliza Hall Institute of Medical Research.

Who has reviewed the research project?

The ethical aspects of this research project have been approved by the National Health Sciences Research Committee (NHSRC) at the Ministry of Health and Health and Research Ethic Committee (HREC) of the Walter and Eliza Hall Institute of Medical Research, Melbourne.

Further information and who to contact

If you want any further information concerning this project or if the participant has any medical problems which may be related to their involvement in the project (for example, any side effects), you can ask us today or during our regular visits or subsequent visits. If you have other questions later, you can contact: Prof Kamija Phiri, Principal Investigator, College of Medicine, Blantyre, 0999 957 048 or Dr Sant-Rayn Pasricha, Principal Investigator from Walter and Eliza Hall Institute of Medical Research Tel: +61393452618

For matters relating to research at the site at which the child is participating, the details of the local site complaints persons are:

Complaints contact numbers

Chikwawa Boma: 0995 835 215 or 0883 536 917

Mfera: 0990 676 012 or 0881 861 069

Mapelera: 0990 676 014 or 0881 861 169

Makwhira: 0994 952 510 or 0883 536 925

If you have any complaints about any aspect of the project, the way it is being conducted or any questions about being a research participant in general, then you may contact:

Reviewing HREC name	**National Health Sciences Research Committee**
HREC Executive Officer	**Dr Collins Mitambo**
Telephone	*0999 397 913*
Email	**mohdoccentre@gmail.com or- cmitambo@gmail.com**

For peer review only - http://bmjopen.bmj.com/site/about/guidelines.xhtml

IRMA Study

Declaration by Parent/Guardian

By signing the consent form you are agreeing that:

- You have read the consent or someone has read it to you in Chichewa.
- You understand the purposes, procedures and risks of the research described in the project.
- You give permission for the child's doctors, other health professionals, hospitals or laboratories outside this hospital to release information to the College of Medicine and The Walter and Eliza Hall Institute of Medical Research concerning the child's disease and treatment for the purposes of this project. You understand that such information will remain confidential.
- You have had an opportunity to ask questions and you are satisfied with the answers you have received.
- You freely agree to the child participating in this research project as described and understand that you are free to withdraw them at any time during the research project without affecting their future health care.
- You understand that you will be given a signed copy of this document to keep.

____
Name of Parent/ Guardian/

__________________________ ______________
Signature or right thumb impression of Parent/ Guardian/ Attendant — Date

____
Name of Witness (*If Parent/ Guardian cannot read and write*)

__________________________ ______________
Signature of Witness — Date

____
Name of Investigator or study staff

__________________________ ______________
Signature of Investigator or study staff — Date

For peer review only

For peer review only - http://bmjopen.bmj.com/site/about/guidelines.xhtml

IRMA Study

Training and Research Unit of Excellence (TRUE), Department of Public Health, College of Medicine, Malawi

University of Melbourne, Victoria, Australia

2. KALATA YOPEMPHA CHILOLEZO

Protocol No. 2213	Version No. 4.0	Date: July 25, 2022

Mutu wa kafukufuku: Kufufuza ubwino ndi chitetezo cha mankhwala okhala ndi ironi komanso mankhwala oteteza ku malungo kwa ana m'Malawi**.**

Oyang'anira kafukufuku: Professor Kamija Phiri ndi Dr Sant-Rayn Pasricha
Bungwe: Training and Research Unit of Excellence (TRUE), Department of Public Health, College of Medicine, Blantyre, Malawi mogwirizana ndi University of Melbourne, Australia

Takuitanani kuti tikupempheni kuti mwana wanu atenge nawo mbali mu kafukufuku amene tikuchita. Muli ololedwa kufunsa mafunso okhudza chilichonse chomwe mukufuna mutadziwa kapena chomwe simukumvetsa pakukambirana kwathu. Musanapange chiganizo chokuti mwana wanu atenge nawo mbali mukafukufuku kapena ayi, mutha kudziwitsa kapena kuuza wachibale, mnzanu kapena munthu wina aliyense za nkhaniyi.

Cholinga cha kafukufuku
Monga mukudziwa, ana ambiri m'Malawi muno ndi kwina konse m'dziko lapansi, amakhala ndi vuto lakuchepekedwa magazi. Mabungwe, kuphatikizapo boma la Malawi amalimbikitsa kuti ana onse alandire iron oonjezera kapena michere yosiyana yosakaniza yoonjezera popewa matenda akuchepa kwa magazi. Iron ndi michere yi zikupangidwa ndi kupezeka kuno ku Malawi. Ngakhale zili chomwechi, pakadali pano sizikudziwika bwinobwino ngati kupereka iron oonjezera kapena michere yosakaniza ndi njira zabwino zopititsa patsogolo umoyo wa ana. Komanso sizikudziwika bwinobwino kuti kodi njira yabwino kwambiri pa njira ziwiri zimenezi ndi iti pothandizira kakulidwe ka ana. Mu kafukufukuyu, tikufuna kufufuza zotsatira za njirazi makamaka pa makulidwe komanso umoyo wa ana. Zotsatira za kafukufukuyu zidzathandiza boma komanso ogwira ntchito za umoyo kupanga chiganizo ngati kuli koyenera kupereka njirazi kwa ana onse mtsogolomu.

N'chifukwa chiyani mwasankhidwa kutenga nawo mbali mu kafukufuku?
Boma la Chikwawa lasankhidwa ndi akuluakulu a Training and Research Unit of Excellence (TRUE) mogwirizana ndi University of Melbourne ku Australia potengera zomwe tikudziwa kale zokhudzana ndi kukula kwa vuto lakuchepa magazi kuno ku Malawi. Mwana wanu amakhala mu dera lomwe lasankhidwali ndipo ali ndi zonse zomuyenereza kutenga nawo mbali mu kafukufukuyu. Pa chifukwa ichi, tikukupemphani kuti mwana wanu atenge nawo mbali mu kafukufukuyu.

Njira zomwe zidzagwiritsidwe ntchito kapena kutsatidwa
Kodi muyenera kuchita chiyani pamene mwatenga mbali mu kafukufukuyu

For peer review only - http://bmjopen.bmj.com/site/about/guidelines.xhtml

Mudzayenera kubwera ndi mwana wanu ku chipatala kuno katatu; 1) tsiku loyamba (lero), 2) patatha miyezi isanu ndi umodzi (6) kutanthauza kuti ku mapeto kolandira mankhwala, 3) patatha miyezi khumi ndi iwiri chiyambireni kafukufukuyu

Pa tsiku loyamba, a kafukufuku adzakufunsani mafunso angapo makamaka okhudzana ndi pakhomo panu, zinthu zomwe mwana wanu amaseweretsa pakhomo, makhalidwe ake atsiku ndi tsiku komanso mmene inu mumaonera za umoyo wanu. Adzayesanso mmene mwana wanu akukulira m'maganizidwe ake ndipo adzachita izi posewera naye. Adzayesanso msinkhu wanu ndi wa mwana, kulemera komanso kanenepedwe ka mkono komanso mutu wa mwana wanu. Pamapeto pa mafunso, adzakupemphani kuti atengeko magazi a mu mtsempha wa pa mkono okwanira 3ml (osakwanira pa supuni yaing'ono). Pa nthawi ya chiwiri yomwe mudzabwerenso, patatha miyezi isanu ndi umodzi (6), zonse zomwe munayesedwa zidzayesedwanso kupatula mafunso okhudza inu ndi za mwana wanu. Kuchita zimenezi zidzatenga maola awiri kapena atatu kuti timalize. Tidzatenganso chimbudzi cha ana onse omwe adzalowe mu kafukufukuyu pa nthawi imene akubwera ku chipatala kuno komaliza.

Tikufuna kukuitananinso mu kalondolondo yemwe adzachitike patatha miyezi khumi ndi iwiri. Dziwani kuti kalondolondo ameneyu adzatengera mmene za chuma zidzakhalire. Komanso tikufuna kukupemphani ngati mungatilore kusunga zonse zotsala kuti tidzathe kugwiritsanso ntchito mukafukufuku, komanso kulumikizana nanu pa nkhani ya kafukufuku mtsogolomu.

Kuyendera makomo

Pamene mwana wanu walowa mu kafukufukuyu, m'modzi mwa othandizira mu kafukufuku adzayendera mwana wanu masabata awiri aliwonse kwa miyezi isanu ndi umodzi (6) pamene akulandira mankhwala komanso mwezi uliwonse kwa miyezi isanu ndi umodzi yotsatira. Adzalemba mu mabuku awo mankhwala amene mwana wamwa mu mmasabata awiri a mmbuyomu, kutenga mankhwala omwe sanagwiritsidwe ntchito ndi kupereka mankhwala oti mwana wanu amwe mu masabata otsatira. Adzakufunsinanso mafunso a umoyo wa mwana wanu monga ngati mwana wanu anatentha thupi, kapena munamutengera ku chipatala kukaonana ndi adokotala kapena kugonekedwa mu chipatala mu masabata awiri angothawo.

Kodi mwana ayenera kuchita chiyani mukafukufukuyi?

Mu kafukufukuyu, inu komanso opereka makhwala sadzadziwa kuti mwana wanu, potenga nawo mbali, ali gulu liti ndipo akulandira mankhwala anji pa magulu anayi amankhwala omwe akhazikitsidwa. Magulu anayi amankhwalawa ndi 1) makhwala oteteza ku malungo ndi ironi wa madzi, 2) mankhwala oteteza ku malungo ndi iron wa ufa, 3) mankhwala oteteza ku malungo ndi 4) mankhwala ena ongofanizira. Mwana wanu adzakhala mugulu limodzi la magulu mwamagulu anayi amenewa. Iye adzapatsidwa mankhwala a mitundu itatu; botolo la mankhwala a madzi, kaphukusi ka timasacheti a mankhwala a ufa ndi mankhwala a malungo. Nthawi ya kafukufuku, adzidzamwa supuni imodzi yothilira shuga ya mankhwala a madzi ndi kaphukusi kamodzi ka mankhwala a ufa tsiku lililonse kwa miyezi isanu ndi umodzi. Mutha kupereka ma dontho a mankhwala a madzi pogwiritsa ntchito kachubu kolembedwa milingo. Mankhwala a ufa atha kuperekedwa pothira mankhwalawo ku chakudya cha

For peer review only - http://bmjopen.bmj.com/site/about/guidelines.xhtml

mwana chomwe chazizira pang'ono. Mankhwala a madzi atha kukhala ndi iron kapena izo zongofanizira, ndipo mankhwala a ufa atha kukhala ndi michere kapena mankhwala ongofanizira a ufa. Mulingo wa mankhwala a madzi wa 1.3 mililita yomwe muzampatsa mwana wanu ndiwolingana ndi mamiligalamuzi 10 a iron ndipo sacheti imodzi ya mankhwala a ufa ndiyolingana ndi mamiligalamu 10 a iron, mamiligalamu 0.3 a vitamin A, mamiligalamu 30 a vitamin C, mamiligalamu 0.16 a foliki asidi ndi mamiligalamu 5 a zinki). Mankhwala omwe adzakhale opanda chilichonse nkati mwake adzaoneka ngati mankhwala ena amene mwana aliyense adzalandire. Mankhwala ofanizira sangagwire ntchito ina iliyonse mnthupi ngakhale amaoneka ngati mankhwala eni eni. Pamene mwana wanu waikidwa mu gulu, adzalandira mankhwala ngati amenewa kwa miyezi isanu ndi umodzi (6).

Ngati mwana wanu wadwala, mumutengere ku chipatala kuti akalandire thandizo ndipo tidzafunsa ngati mwana anadwala pa nthawi imene tizidzayendera makomo masabata awiri aliwonse.

Ufulu otenga nawo mbali komanso kutuluka mu kafukufuku

Kodi mwana akuyenera kutenga mbali mu kafukufukuyu?

Kutenga nawo mbali mu kafukufukuyu ndikosakakamiza kwa wina aliyense. Ngati simukufuna kuti mwana wanu atenge nawo mbali, muli ololedwa kutero. Ngati mwavomera kuti mwana wanu atenge nawo mbali mu kafukufukuyu kenako mwasintha maganizo pakati pa kafukufukuyu, muli ololedwa kumutulutsa mwana wanu nthawi ina iliyonse imene mwaganiza kutero. Ngati mwavomera kuti mwana wanu atenge mbali, mudzapatsidwa kalata ya mtengambali komanso kalata yopempha chilolezo yomwe mudzasainira ndipo mudzapatsidwa kalata imodzi kuti musunge.

Chingachitike ndi chiyani nditamutulutsa mwana mu kafukufukuyu?

Ngati mutaganiza zomutulutsa mwana wanu pakati pa kafukufuku, a dotolo ndi ena onse ochita kafukufukuyu sadzatenganso chilichonse chokhudza inu kapena mwana wanu. Dziwani kuti zonse zomwe tinatolera kale zidzasungidwa ndipo zidzakhala nawo mbali imodzi ya ma lipoti a kafukufukuyi. Dziwaninso kuti, kutuluka mukafukufukuyi simudzalandira chilango china chilichonse ndipo sizidzakhudza chithandizo chomwe mumalandira ku chipatala kapena kuononga ubale wanu ndi madotolo anu, kapenanso ubale wawo ndi masukulu a ukachenjede omwe akuchita kafukufukuyu.

Kodi pali njira inanso yotengera nawo mbali mu kafukufukuyu?

Dziwani kuti monga tinanenela muja kuti kutenga nawo mbali mukafukufukuyi sizokakamiza. Ngati mwasankha kusatenga nawo mbali mu kafukufukuyu mutha kukapeza thandizo la vuto la kuperewera kwa magazi ndi za thanzi kwina kulikonse komwe inu mungafune.

Zoopsya zomwe zingachitike

Kodi zoopsya zomwe zingachitike potenga mbali mukafufufukuyu ndi chiyani?

Iron oonjezera komanso ufa wa michere zakhala zikupatsidwa kwa ana miyandamiyanda ndipo ndi osaopsya. Ngakhale zili chonchi, tikhala tikufufuza ngati pali zina zoopsya zomwe zingabwere chifukwa cha mankhwalawa. Ngati zingachitike zina zoopsya sizitenga nthawi yambiri zisanathe kuchokera pa nthawi yomwe

For peer review only - http://bmjopen.bmj.com/site/about/guidelines.xhtml

mankhwala asiya kuperekedwa. Ngati mwana wanu akuonetsa zizindikiro zosakhala bwino kapena mukudandaula za iye, chonde tiuzeni. Ngakhale zili chonchi, zina zotsatira mankhwalawa zitha kukhala zoopsya, zokhala nthawi yaitali zisanathe komanso zokhalitsa mpaka kale. Ngati zotsatira zoopsya zaoneka, tidzasiya kumupatsa mwana mankhwalawa. Amene akuchita kafukufuku pa mwanayu, adzayenera kupereka upangiri oyenera wothana ndi vutolo ndi inu. Koma nthawi zambiri, palibe icho choopsya chomwe chinaoneka kwa ana omwe akulandira iron komanso michere imene tatchula ija.

Zotsatira zosayembekezeleka	Zimachitika pafupipafupi?	Ndi zoopsya motani	Zingakhale nthawi yaitali bwanji zisanathe?
Kupweteka kwa m'mimba, chilungulira, kudzimbidwa kapena kutsekula m'mimba	Mwina m'modzi mwa ana anayi	Ndizosaopsya kwambiri	Masiku ochepa kapena nthawi yomwe akulandira chithandizo; koma zopililika
Matenda (monga kutsekula m'mimba)	Izi zifanana ndi m'mene zingachitikire mwa ana amene sakulandira iron kapena zitha kuchitikachitika	Ngati mene zingachitikire mwa ana amene sakulandira iron kapena zitha kuchitika moopsa pang'ono	Masiku ochepa
Kuda kwa chimbudzi – izi sizotsatira zoopsa ndipo sizoopsa kwa mwana	Zimachitikachitika	Sizoopsya ndipo musadandaule nazo	Nthawi yomwe akulandira mankhwala.

Nthawi yotenga magazi munthu atha kumva kupweteka kobaya pang'ono, kutupa kapena kusintha mtundu pa malo omwe abaidwa. Madotolo athu odziwa bwino ntchito adzaonetsetsa kuti pali ukhondo komanso kuchepetsa ululu.

Kodi phindu lotenga nawo mbali mu kafukufukuyu ndi lotani?

Sitinganene mwandithendithe kuti mwana adzapeza phindu lililonse mu kafukufukuyu; ngakhale zili chonchi, zina mwa izo zomwe mwana angapindule nazo ndizo; kuthandizidwa ku vuto lakuchepa kwa magazi ngati mwana wanu walandira iron, kukhala wa mphamvu ndi nyonga, kukhala ndi umoyo wabwino, kukula mu nzeru ndi msinkhu. Tidzakupatsani zotsatira za magazi a mwana makamaka kuchuluka kwa iron. Ngati vuto lochepa kwa magazi lili lalikulu kapena mwana wanu sakukula bwino, tidzamutumiza mwana wanu ku chipatala choyenelera kuti akalandire thandizo pa nthawi ya kafukufukuyu. Kutenga nawo mbali kwa mwana wanu kudzatithandiza kuti tipeze njira za bwino zothana ndi vuto lakuchepa kwa magazi ndipo zidzathandiza mmene tingathandizire ana ena onse dziko lonse lapansi. Dziwaninso kuti pamene mwabwera kuchipatala pa nthawi yakafukufukuyi tidzakubwezerani ndalama yanu yoyendera yolingana ndi 10 USD.

For peer review only - http://bmjopen.bmj.com/site/about/guidelines.xhtml

IRMA Study

Kusunga chinsinsi

Kodi uthenga wa mwana omwe mudzatenge udzagwiritsidwa ntchito motani?
Pamene mwasaina kalata yopereka chilolezo, ndiye kuti mukupereka chilolezo kwa dotolo komanso onse amene akuthandizira mu kafukufukuyu kutenga ndi kugwiritsa ntchito uthenga okhudza mwana wanu mu kafukufukuyu basi. Uthenga uliwonse omwe udazatengedwe okhudza mwana wanu mogwirizana ndi kafukufukuyu, pamene tikusindikiza, kapena kugawidwa kwa ena, udzaperekedwa mu njira yokuti palibe amene adzaziwe dzina la mwana wanu kapena kulondoloza mwana wanu. Uthengawo udzasungidwa bwino ku bungwe lomwe likuyendetsa za kafukufukuyi komanso udzagawidwa kwa a kadaulo a sukulu ya ukachenjede a ku Australia kudzera mu uthenga wa pa internet omwe udzakhale wotetezedwa. Uthengawu udzagwiritsidwa ntchito mu kafukufukuyu komanso kafukufuku wina ofanana naye kutsogoloko. Molingana ndi ndondomeko ya kasungidwe ka uthenga wa kafukufuku okhudza ana, tidzasunga uthengawu kwa zaka 25 ndipo kenaka udzafutidwa. Uthenga wa mwana udzagwiritsidwa ntchito pa zolinga za kafukufukuyu ndipo sudzaulutsidwa pokhapokha ngati inu mwavomereza ndi kupereka chilolezo, kupatula pamene uthengawu ukufunidwa molingana ndi lamulo.

Pa nthawi imene kafukufuku akuchitika, tidzafuna kudziwa nthenda kapena vuto linalililonse lomwe mwana angapezeke nalo pamene wapita ku chipatala kukapimidwa. Pamene mwasaina kalata ya chilolezo ndiye kuti mukugwirizana ndi zokuti onse amene akuthandizira mu kafukufukuyu akhoza kuona chiphaos cha umoyo cha mwana kuti afufuze ngati umoyo wa mwanawo ukumuyenereza kutenga nawo mbali mu kafukufukuyu.

Uthenga omwe udzatengedwa nthawi yakafukufuku kuphatikizapo omwe udzatengetengedwe mu chiphaso cha umoyo wa mwana zidzafufuzidwa mokwana ndi cholinga chofuna kuona ngati pali mgwirizano pakati pa njira zomwe zidzatsatidwe mu kafukufukuyu ndi uthenga omwe watengedwa. Izi zidzachitidwa ndi akuluakulu a kafukufuku amene ali ololedwa kutero. Pamene mwasaina kalata ya chilolezo, ndiye kuti mwagwirizana ndi zakuti mudzalola kuonetsa kapena kupereka uthenga oyenera kwa onse amene akugwira ntchito mu kafukufuku.

Kugwiritsa ntchito uthenga mtsogolo
Kodi magazi kapena chimbuzi chomwe chidzatengedwa zidzagwira ntchito yanji?
Mukuyenera kudzapereka chilolezo pa nthawi ya kafukufuku kuti mwana wanu adzatengedwe magazi. Kutenga magazi ndi mbali imodzi yofunika kwambiri mu kafukufukuyu.

Magazi amene adzatengedwa adzaunikidwa kuti tione kuchuluka kwa iron ndi cholinga chakuti, pa mapeto a kafukufukuyu tidzaone ngati mankhwala omwe mwana amalandira agwira ntchito kapena ayi. Tidzasunganso magazi otsala ku sukulu ya ukachenjede yomwe ikutsogolera kafukufukuyu ku Blantyre kwa zaka zisanu kuchokera tsiku lomaliza la kafukufuku. Ma sampulo a magazi adzasungidwa kuti wina aliyense asadzazindikire mwana wanu pogwiritsa ntchito nambala ya chinsinsi.

Tidzatha kuyesanso zina mu sampulo ya magazi otsala mu kafukufuku wina amene angafanane ndi amene tikupangayi mtsogolomu.

Kuyesa ma jinzi

Tidzatenganso magazi kuti tikayese DNA wa mwana wanu koma izi zidzachitika mtsogolomu. Komabe, sitidzayesa matenda ochokera ku mtundu, kotero zotsatira za kuyesa kumeneku sikudzabweretsa nkhawa iliyonse kwa mwana komanso banja lonu ndipo palibenso kuthekera kulikonse koyesa matenda a ku kumtundu mu sampulo ya magazi imeneyi. M'malo mwake, tikuyembekezera kudzayesa magaziwa kuti tidziwe ngati pali mgwirizano ulionse pakati pa zotengera za ku mtundu ndi vuto la kusowa kwa magazi, nthanzi, makulidwe, kapena matenda. Magazi ndi zotsatira zake, sizidzaperekedwa kwa wina aliyense kupatula amene akugwira ntchito mu kafukufukuyu. Ngati zipangizo zoyezela zidzavute kwathu kuno, ndiye kuti magaziwa adzatumizidwa ku maiko a kunja ndipo pamapeto ake tidzakawataya moyenelera.

Nanga ngati patapezeka uthenga wina pamene kafukufukuyu akuchitika?
Nthawi zina, pakati pa kafukufuku, pamatha kubwera uthenga wina wa chilendo okhudza mankhwala amene akufufuzidwa. Ngati izi zitachitika, dokotala akuyenera kukufotokozerani ndipo muli ndi ufulu osankha kupitiliza kapena kumutulutsa mwana wanu mu kafukufukuyu. Ngati mwaganiza zomutulutsa mwana wanu, a dotolo amene akutenga mbali mu kafukufuku adzapanga chikonzero chakuti mwana wanu apitirize kulandira thandizo linalonse la ku chipatala moyenera. Mgati mudzaganiza zopitiliza kutenga nawo mbali, mudzayenera kusaina kalata ina yachilolezo yokonzedwa molingana ndi kusinthako.

Kodi mwana akhonza kulandira mankhwala ena a iron mu nthawi ya yakafukufukuyu?
Pa nthawi imene mwana akutenga nawo mbali mu kafukufukuyu, timalimbikitsa kuti mwana asalandirenso iron wina kupatula yekhayo amene ife tikupereka chifukwa ngati mutero, ndiye kuti mwana wanu akulandira iron kawiri zimenene zitha kukhala zoopsa kwa mwana wanu komanso zingasokoneze zotsatira za kafukufuku. Chonde, tidziwitseni ngati mwana akulandira iron wina kupatula amene tikupatseniyi.

Kodi kafukufukuyu akhonza kuimitsidwa mwadzidzidzi?
Eya. Kafukufukuyu akhonza kuimitsidwa mosayembekezereka pa zifukwa zosiyanasiyana monga kusowa kwa thandizo la ndalama loyendetsera kafukufuku, kapena zizindikiro zoopsya zomwe zingabwere chifukwa cholandira mankhwala.

Chidzachitike ndi chiyani pamene kafukufuku wafika pamapeto?
Pa mapeto a kafukufukuyu tidzayesa mwana wanu kuti tione ngati ali ndi vuto lakuchepa kwa magazi kapena ayi. Ngati mwana wanu adzapezeke ndi vutoli, tidzamutumiza ku chipatala kuti akalandire thandizo. Tikadzamaliza kafukufuku ndi kulemba lipoti lomaliza, tidzapereka zotsatira za kafukufukuyu kwa alangizi a za umoyo ndi m'zipatala.

Madandaulo ndi chipukuta misonzi
Ngati muli ndi dandaulo lokhudza kafukufukuyu kapena mukufuna kudziwa zambiri za kafukufukuyi mukhonza kudziwitsa Mkulu woyang'anira bungwe lomwe limaona za

For peer review only - http://bmjopen.bmj.com/site/about/guidelines.xhtml

madandaulo a anthu omwe akutenga nawo mbali mu kafukufuku la National Health Sciences Research Committee (NHSRC) limene lili ku unduna wa za umoyo wa boma la Malawi ku Lilongwe, poyimba foni pa: 0999 397 913 komanso uthenga wodzera pa intaneti polembela ku: cmitambo@gmail.com kapena mohdoccentre@gmail.com.

Ngati amene akutenga nawo mbali wavulala kapena wapezeka ndi vuto lomwe ladza kamba ka kafukufukuyu, mukuyenera kudziwitsa ogwira ntchito mu kafukufukuyu mwa msanga, ndipo iwo adzakuthandizani pokonza chithandizo choyenera cha mankhwala.

Chipukuta misonzi
Mudzabwezeredwa ndalama m'makwacha yofanana mphamvu ndi 10 USD ya nthawi imene mwagwiritsa ntchito potenga nawo gawo mu kafukufukuyu kuphatikiza ndalama ya mayendedwe pa nthawi yomwe mwapemphedwa kubwera kuchipatala.

Kuyankha mafunso/ anthu omwe mungalumikizane nawo
Kodi amene akonza ndi kupereka thandizo la ndalama mu kafukufukuyu ndi ndani? Amene akuchita kafukufukuyu ndi nkulu wa kafukufuku a Kamija Phiri a ku sukulu ya ukachenjede ya madotolo kuno ku Malawi ndi a Sant-Rayn Pasricha a ku sukulu ya ukachenjede ya Melbourne ku Australia.

Amene apereka chilolezo chochitira kafukufukuyu ndi ndani?
Amene apereka chilolezo chochitira kafukufukuyu ndi mabungwe amene amaona za ufulu wa anthu amene akutenga nawo mbali mu kafukufuku. Mabungwewa ndi la ku Malawi ndipo lina ndi la ku Australia.

Ngati mukufuna kudziwa zambiri ndi amene mungalumikizane nawo
Ngati mukufuna kudziwa zambiri kapena amene watenga nawo mbali mu kafukufukuyu wakumana ndi vuto lomwe ladza kamba ka kafukufukuyu (mwa chitsanzo, zizindikiro zoopsya zomwe zadza chifukwa cholandira mankhwala athu), mutha kufunsa pompano kapena nthawi iliyonse yomwe tidzakuyenderani. Koma ngati mungakhale ndi mafunso nthawi ina iliyonse, mutha kuimba lamya ndi kuyankhulana ndi mkulu wa kafukufukuyu amene akupezeka ku Blantyre, Professor Kamija Phiri pa nambala iyi: 0999 957 048 kapena Dr. Sant-Rayn Pasricha, mkulu wa kafukufuku ku chokera ku Australia, Tel: +61393452618

Pa nkhani zokhudza kafukufuku pa malo kapena chipatala chimene mwana akutengerapo mbali, mutha kupereka madandaulo anu kwa munthu wa chipatalapo kudzera pamanambala awa:

Chikwawa Boma:	0995 835 215 or 0883 536 917
Mfera:	0990 676 012 or 0881 861 069
Mapelera:	0990 676 014 or 0881 861 169
Makwhira:	0994 952 510 or 0883 536 925

For peer review only - http://bmjopen.bmj.com/site/about/guidelines.xhtml

Ngati muli ndi madandaulo ena okhudzana ndi kafukufukuyu, mmene akuyendetsedwera, kapena mafunso ena alionse okhudzana ndi otenga mbali, mukhonza kulumikizana ndi:

Reviewing HREC name	**National Health Sciences Research Committee**
HREC Executive Officer	**Dr Collins Mitambo**
Telephone	*0999 397 913*
Email	**cmitambo@gmail.com** or **mohdoccentre@gmail.com**

Kuvomereza kwa oyang'anira mwana

Ngati mupange chisankho chotenga nawo mbali mukafukufukuyu, mudzapemphedwa kuti musayine kalatayi, ndipo kalata ina idzasungidwa ndiife ina ndi inu ngati umboni wakuti mwavomera kutenga nawo mukafukufukuyu. Posayinira kalata ya chilolezoyi mukuvomereza kuti:

- Mwawerenga kapena kuwerengeredwa kalata yopempha chilolezoyi mu Chichewa.
- Mwamvetsetsa cholinga, ndondomeko komanso zovuta zina za kafukufukuyu.
- Mukupereka chilolezo kwa ma dotolo komanso onse a zaumoyo kupereka uthenga uliwonse okhudza umoyo wa mwana wanu ku College of Medicine ndinso University of Melbourne *ndi cholinga chofuna kuthandiza nawo mu kafukufukuyu ndipo muli ndi chitsimikizo kuti uthenga wa umoyo wa mwanawu udzasungidwa mwa chinsinsi.*
- Munapatsidwa mwayi ofunsa mafunso ndipo mwakhutira ndi mayankho omwe mwapatsidwa.
- Mukuvomera mwa ufulu kuti mwana wanu atenge nawo mbali mu kafukufukuyu molingana ndi mmene mwafotokozeredwa ndipo mukudziwa kuti muli ndi ufulu osiya kutenga nawo mbali mu kafukufukuyu nthawi inailiyonse ndipo izi sizidzakhudzana ndi kulandira chithandizo ku chipatala mtsogolomo.
- Mukudziwanso kuti mukuyenera kusunga kalata imodzi yopempha chilolezo yomwe ya sainidwa.

____
Dzina la kholo kapena womuyang'anira mwana

______________________________ ________________
Saini la kholo kapena womuyang'anira mwana Tsiku

____
Dzina la mboni (*ngati kholo kapena womuyang'anira mwana sadziwa kulemba kapena kuwerenga*)

______________________________ ________________
Saini la mboni Tsiku

For peer review only - http://bmjopen.bmj.com/site/about/guidelines.xhtml

Dzina la woyang'anira kafukufuku

Saini ya woyang'anira kafukufuku

Tsiku

For peer review only

For peer review only - http://bmjopen.bmj.com/site/about/guidelines.xhtml

Reporting checklist for protocol of a clinical trial.

Based on the SPIRIT guidelines.

Instructions to authors

Complete this checklist by entering the page numbers from your manuscript where readers will find each of the items listed below.

Your article may not currently address all the items on the checklist. Please modify your text to include the missing information. If you are certain that an item does not apply, please write "n/a" and provide a short explanation.

Upload your completed checklist as an extra file when you submit to a journal.

In your methods section, say that you used the SPIRITreporting guidelines, and cite them as:

Chan A-W, Tetzlaff JM, Gøtzsche PC, Altman DG, Mann H, Berlin J, Dickersin K, Hróbjartsson A, Schulz KF, Parulekar WR, Krleža-Jerić K, Laupacis A, Moher D. SPIRIT 2013 Explanation and Elaboration: Guidance for protocols of clinical trials. BMJ. 2013;346:e7586

		Reporting Item	Page Number
**Administrative information**			
Title	#1	Descriptive title identifying the study design, population, interventions, and, if applicable, trial acronym	1
Trial registration	#2a	Trial identifier and registry name. If not yet registered, name of intended registry	4
Trial registration: data set	#2b	All items from the World Health Organization Trial Registration Data Set	4
Protocol version	#3	Date and version identifier	31
Funding	#4	Sources and types of financial, material, and other support	31
Roles and responsibilities: contributorship	#5a	Names, affiliations, and roles of protocol contributors	30

For peer review only

Roles and responsibilities: sponsor contact information	#5b	Name and contact information for the trial sponsor	31
Roles and responsibilities: sponsor and funder	#5c	Role of study sponsor and funders, if any, in study design; collection, management, analysis, and interpretation of data; writing of the report; and the decision to submit the report for publication, including whether they will have ultimate authority over any of these activities	31-32
Roles and responsibilities: committees	#5d	Composition, roles, and responsibilities of the coordinating centre, steering committee, endpoint adjudication committee, data management team, and other individuals or groups overseeing the trial, if applicable (see Item 21a for data monitoring committee)	22-23
**Introduction**			
Background and rationale	#6a	Description of research question and justification for undertaking the trial, including summary of relevant studies (published and unpublished) examining benefits and harms for each intervention	6-10
Background and rationale: choice of comparators	#6b	Explanation for choice of comparators	14
Objectives	#7	Specific objectives or hypotheses	10-12
Trial design	#8	Description of trial design including type of trial (eg, parallel group, crossover, factorial, single group), allocation ratio, and framework (eg, superiority, equivalence, non-inferiority, exploratory)	12
**Methods: Participants, interventions, and outcomes**			
Study setting	#9	Description of study settings (eg, community clinic, academic hospital) and list of countries where data will be collected. Reference to where list of study sites can be obtained	12
Eligibility criteria	#10	Inclusion and exclusion criteria for participants. If applicable, eligibility criteria for study centres and individuals who will	12-13

For peer review only

		perform the interventions (eg, surgeons, psychotherapists)	
Interventions: description	#11a	Interventions for each group with sufficient detail to allow replication, including how and when they will be administered	14
Interventions: modifications	#11b	Criteria for discontinuing or modifying allocated interventions for a given trial participant (eg, drug dose change in response to harms, participant request, or improving / worsening disease)	14
Interventions: adherance	#11c	Strategies to improve adherence to intervention protocols, and any procedures for monitoring adherence (eg, drug tablet return; laboratory tests)	20
Interventions: concomitant care	#11d	Relevant concomitant care and interventions that are permitted or prohibited during the trial	12-13
Outcomes	#12	Primary, secondary, and other outcomes, including the specific measurement variable (eg, systolic blood pressure), analysis metric (eg, change from baseline, final value, time to event), method of aggregation (eg, median, proportion), and time point for each outcome. Explanation of the clinical relevance of chosen efficacy and harm outcomes is strongly recommended	23
Participant timeline	#13	Time schedule of enrolment, interventions (including any run-ins and washouts), assessments, and visits for participants. A schematic diagram is highly recommended (see Figure)	15-22
Sample size	#14	Estimated number of participants needed to achieve study objectives and how it was determined, including clinical and statistical assumptions supporting any sample size calculations	24
Recruitment	#15	Strategies for achieving adequate participant enrolment to reach target sample size	15
**Methods: Assignment of interventions (for controlled trials)**			
Allocation: sequence generation	#16a	Method of generating the allocation sequence (eg, computer-generated random numbers), and list of any factors for stratification. To reduce predictability of a random sequence, details of any planned restriction (eg, blocking) should be provided in a separate document that is unavailable to those who enrol participants or assign interventions	14-15

For peer review only

Allocation concealment mechanism	#16b	Mechanism of implementing the allocation sequence (eg, central telephone; sequentially numbered, opaque, sealed envelopes), describing any steps to conceal the sequence until interventions are assigned	14-15
Allocation: implementation	#16c	Who will generate the allocation sequence, who will enrol participants, and who will assign participants to interventions	14-15
Blinding (masking)	#17a	Who will be blinded after assignment to interventions (eg, trial participants, care providers, outcome assessors, data analysts), and how	14-15
Blinding (masking): emergency unblinding	#17b	If blinded, circumstances under which unblinding is permissible, and procedure for revealing a participant's allocated intervention during the trial	14-15
**Methods: Data collection, management, and analysis**			
Data collection plan	#18a	Plans for assessment and collection of outcome, baseline, and other trial data, including any related processes to promote data quality (eg, duplicate measurements, training of assessors) and a description of study instruments (eg, questionnaires, laboratory tests) along with their reliability and validity, if known. Reference to where data collection forms can be found, if not in the protocol	15-22
Data collection plan: retention	#18b	Plans to promote participant retention and complete follow-up, including list of any outcome data to be collected for participants who discontinue or deviate from intervention protocols	15-22
Data management	#19	Plans for data entry, coding, security, and storage, including any related processes to promote data quality (eg, double data entry; range checks for data values). Reference to where details of data management procedures can be found, if not in the protocol	24-25
Statistics: outcomes	#20a	Statistical methods for analysing primary and secondary outcomes. Reference to where other details of the statistical analysis plan can be found, if not in the protocol	24
Statistics: additional analyses	#20b	Methods for any additional analyses (eg, subgroup and adjusted analyses)	24

For peer review only

Statistics: analysis population and missing data	#20c	Definition of analysis population relating to protocol non-adherence (eg, as randomised analysis), and any statistical methods to handle missing data (eg, multiple imputation)	24
**Methods: Monitoring**			
Data monitoring: formal committee	#21a	Composition of data monitoring committee (DMC); summary of its role and reporting structure; statement of whether it is independent from the sponsor and competing interests; and reference to where further details about its charter can be found, if not in the protocol. Alternatively, an explanation of why a DMC is not needed	22-23
Data monitoring: interim analysis	#21b	Description of any interim analyses and stopping guidelines, including who will have access to these interim results and make the final decision to terminate the trial	25
Harms	#22	Plans for collecting, assessing, reporting, and managing solicited and spontaneously reported adverse events and other unintended effects of trial interventions or trial conduct	23-24
Auditing	#23	Frequency and procedures for auditing trial conduct, if any, and whether the process will be independent from investigators and the sponsor	25
**Ethics and dissemination**			
Research ethics approval	#24	Plans for seeking research ethics committee / institutional review board (REC / IRB) approval	26
Protocol amendments	#25	Plans for communicating important protocol modifications (eg, changes to eligibility criteria, outcomes, analyses) to relevant parties (eg, investigators, REC / IRBs, trial participants, trial registries, journals, regulators)	26
Consent or assent	#26a	Who will obtain informed consent or assent from potential trial participants or authorised surrogates, and how (see Item 32)	19
Consent or assent: ancillary studies	#26b	Additional consent provisions for collection and use of participant data and biological specimens in ancillary studies, if applicable	26
Confidentiality	#27	How personal information about potential and enrolled participants will be collected, shared, and maintained in order to protect confidentiality before, during, and after the trial	26

For peer review only

Declaration of interests	#28	Financial and other competing interests for principal investigators for the overall trial and each study site	30
Data access	#29	Statement of who will have access to the final trial dataset, and disclosure of contractual agreements that limit such access for investigators	25
Ancillary and post trial care	#30	Provisions, if any, for ancillary and post-trial care, and for compensation to those who suffer harm from trial participation	
Dissemination policy: trial results	#31a	Plans for investigators and sponsor to communicate trial results to participants, healthcare professionals, the public, and other relevant groups (eg, via publication, reporting in results databases, or other data sharing arrangements), including any publication restrictions	22
Dissemination policy: authorship	#31b	Authorship eligibility guidelines and any intended use of professional writers	30
Dissemination policy: reproducible research	#31c	Plans, if any, for granting public access to the full protocol, participant-level dataset, and statistical code	31
**Appendices**			
Informed consent materials	#32	Model consent form and other related documentation given to participants and authorised surrogates	26
Biological specimens	#33	Plans for collection, laboratory evaluation, and storage of biological specimens for genetic or molecular analysis in the current trial and for future use in ancillary studies, if applicable	22

None The SPIRIT Explanation and Elaboration paper is distributed under the terms of the Creative Commons Attribution License CC-BY-NC. This checklist can be completed online using https://www.goodreports.org/, a tool made by the EQUATOR Network in collaboration with Penelope.ai

For peer review only
